# Systematic literature review of real-world evidence on overall survival in cancer patients before and after the approval of anti-PD-(L)1 therapy

**DOI:** 10.3389/fonc.2025.1615795

**Published:** 2025-08-04

**Authors:** Dweeti Nayak, Katherine G. Akers, Andrew M. Frederickson, Yves P.V. Mbous, Raquel Aguiar-Ibáñez

**Affiliations:** ^1^ Precision AQ, New York, NY, United States; ^2^ Merck & Co., Inc., Rahway, NJ, United States; ^3^ Merck Canada Inc., Kirkland, QC, Canada

**Keywords:** anti-PD-1, anti-PD-L1, immune checkpoint inhibitors, non-small cell lung cancer, renal cell carcinoma, melanoma, overall survival, real-world evidence

## Abstract

**Background:**

The development and regulatory approval of anti-programmed death (ligand) 1 (anti-PD-(L)1) agents, based on positive clinical trial results, has dramatically changed clinical practice and treatment paths in oncology. However, the effectiveness of anti-PD-(L)1 therapy in real-world settings is not well understood. Therefore, it is important to summarize real-world evidence on the overall survival (OS) of patients with specific tumor types prior to and following the regulatory approval of anti-PD-(L)1 therapy.

**Methods:**

A systematic literature review including observational studies worldwide reporting the OS of patients receiving conventional first-line pharmacological therapy for advanced/metastatic non-small cell lung cancer (NSCLC), renal cell carcinoma (RCC), or melanoma in the anti-PD-(L)1 pre-approval era and similar patients receiving first-line anti-PD-(L)1 therapy in the post-approval era was conducted. For each tumor type, studies were selected from a pre-approval era, defined as a period beginning 5 years before the first approval of an anti-PD-(L)1 agent and ending the year before its approval for first-line therapy, and a post-approval era, defined as a period beginning the year that an anti-PD-(L)1 agent was approved for first-line therapy and ending in 2023. Relevant studies were identified through MEDLINE and Embase searches. Study selection, data extraction, and quality assessment were conducted by two independent reviewers. Median OS (mOS) was summarized within each tumor type and descriptively compared across the pre- and post-approval eras.

**Results:**

A total of 86, 44, and 35 studies evaluating first-line treatments for advanced/metastatic NSCLC, RCC, and melanoma, respectively, were included. Post-approval mOS in patients treated with anti-PD-(L)1 therapy tended to be numerically longer than pre-approval mOS in patients treated with conventional therapy within certain patient and treatment categories. For example, pre-approval mOS ranged from 6.9 to 18.4 months (n=18 treatment groups), and post-approval mOS ranged from 10.6 to 46.2 months in NSCLC patients with PD-L1 tumor expression ≥50% who received anti-PD-(L)1 monotherapy (n=33; with mOS not reached for n=3). In RCC patients classified as high-risk, pre-approval mOS ranged from 2 to 10.3 months (n=7), and post-approval mOS ranged from 7.8 to 24.3 months (n=4). Also, in melanoma patients with any BRAF mutation, pre-approval mOS was 14.2 months (n=1), and post-approval mOS ranged from 15.9 to 51.2 months (n=6; with mOS not reached for n=3).

**Conclusion:**

A survival benefit in real-world practice was observed for patients with advanced/metastatic NSCLC, RCC, or melanoma receiving first-line anti-PD-(L)1 therapy after its regulatory approval when compared with patients treated with conventional care before anti-PD-(L)1 therapy approval. This supports the use of anti-PD-(L)1 therapy as a standard of care in many countries.

## 1 Introduction

The development and regulatory approval of immune checkpoint inhibitors (ICIs) has dramatically changed the way cancer is treated in clinical practice (1, 2). ICIs are immunotherapy agents that inhibit T cell immune checkpoints leveraged by tumor cells to evade recognition and destruction by the immune system, thereby restoring the body’s ability to effectively attack tumors (1, 2). After the first regulatory approval of an ICI, the anti-cytotoxic T lymphocyte antigen 4 (anti-CTLA-4) agent ipilimumab, several anti-programmed death (ligand) 1 (anti-PD-(L)1) agents—pembrolizumab, nivolumab, atezolizumab, avelumab, durvalumab, and cemiplimab—have been approved for the treatment of patients with different types of cancer, including advanced/metastatic non-small cell lung cancer (NSCLC), renal cell carcinoma (RCC), and melanoma (2). For these tumor types, anti-PD-L1 therapy is approved as monotherapy or in combination with other immunotherapy agents or chemotherapy in first-line and/or subsequent therapy settings.

The regulatory approval of cancer treatments is largely based on the findings of randomized controlled trials (RCTs), which tightly control potential sources of variability to improve the validity and reliability of the efficacy and safety measures of investigated treatments. However, treatment efficacy as measured in RCTs may not directly translate to real-world settings due to differences between patients enrolled in RCTs versus the more heterogeneous populations seen in less controlled, clinical practice environments, including differences in demographics, functional status, comorbid conditions, adherence and concomitant (3). For example, patients enrolled in RCTs tend to be younger, have better performance status, and have fewer comorbid conditions than patients treated in real-world settings (4, 5). Thus, real-world evidence, including data from observational studies, has the potential to provide additional evidence of the benefits of anti-PD-(L)1 therapy on patient outcomes. For example, previous observational studies (6–8) and evidence summaries (9–12) describe the clinical outcomes of patients with certain cancer types, including advanced/metastatic NSCLC and melanoma, in real-world settings before and after the regulatory approval of ICIs in general. However, no previous evidence summaries have identified and summarized real-world data on outcomes in similar populations of cancer patients before versus after the approval of anti-PD-(L)1 therapy, in particular.

To demonstrate the value and improvements in outcomes that anti-PD-(L)1 therapy has brought to advanced/metastatic cancer patients in clinical practice, there is a need to understand available real-world evidence on the overall survival (OS) of patients with specific tumor types who would currently be eligible for treatment with an anti-PD-(L)1 agent but were treated with conventional care before the regulatory approval of anti-PD-(L)1 therapy and of patients treated with anti-PD-(L)1 agents after their approval by performing a systematic literature review (SLR).

## 2 Methods

An SLR was conducted following the Cochrane Handbook for Systematic Reviews (13) and the National Institute for Health Care and Excellence guidelines manual (14) and is reported according to the Preferred Reporting Items for Systematic Reviews and Meta-analyses (PRISMA) (15).

### 2.1 Inclusion and exclusion criteria

Study eligibility criteria were defined in terms of population, interventions, comparisons, outcome, study design and time (PICOTS) (**Table 1**).

**Table 1:** PICOTS study selection criteria.

Criteria	Inclusion criteria
Population	• NSCLC: Adult (≥18 years) patients with NSCLC meeting one or more of the following indications: o Patients with locally advanced/stage III (but not candidates for surgical resection or definitive chemoradiation), metastatic, or recurrent NSCLC with no EGFR or ALK aberrations who received no prior systemic therapy o Patients with metastatic non-squamous NSCLC with no EGFR or ALK aberrations who received no prior systemic therapy o Patients with metastatic squamous NSCLC who received no prior systemic therapy • RCC: Adult (≥18 years) patients with advanced RCC who received no prior systemic therapy • Melanoma: Adult and pediatric (≥12 years) patients with advanced, unresectable, or metastatic melanoma (with or without BRAF V600 mutation) who received no prior systemic therapy
Interventions	Pre-approval era: Any non-ICI pharmacological treatment
	Post-approval era: Any of the following anti-PD-(L)1 agents delivered alone or in combination with other pharmacological treatments • NSCLC: atezolizumab, cemiplimab, durvalumab, nivolumab, or pembrolizumab • RCC: avelumab, nivolumab, or pembrolizumab • Melanoma: atezolizumab, nivolumab, or pembrolizumab
Comparators	No restrictions
Outcomes	Overall survival (OS)
Time (start of patient data collection)	Pre-approval era: • NSCLC: 2010-2015 • RCC: 2010-2017 • Melanoma: 2009-2013
	Post-approval era: • NSCLC: 2016-2023 • RCC: 2018-2023 • Melanoma: 2014-2023
Study design	Observational studies
Other	English language

ALK, anaplastic lymphoma kinase; EGFR, epidermal growth factor receptor; ICI, immune checkpoint inhibitor; NSCLC, non-small cell lung cancer; PD-(L)1, programmed death (ligand)-1; RCC, renal cell carcinoma.

#### 2.1.1 Population

Eligible studies included patients receiving first-line therapy for advanced/metastatic NSCLC, RCC, or melanoma who met one or more current indications for treatment with a United States (US) Food and Drug Administration (FDA)- or European Medicines Agency (EMA)-approved anti-PD-(L)1 agent [i.e., atezolizumab (16, 17), avelumab (18, 19), cemiplimab (20, 21), durvalumab (22, 23), pembrolizumab (24, 25), or nivolumab (26, 27)]. Of note, as study selection was guided by stringent definitions of both pre- and post-approval eras and the populations of patients eligible for treatment with an anti-PD-(L)1 agent, FDA and EMA approval dates and first-line indications were used, respectively, to develop these definitions. However, no geographical criteria for study selection were imposed.

#### 2.1.2 Interventions/comparators

For the pre-approval era, eligible studies evaluated conventional care, defined as any non-ICI pharmacological treatment as appropriate for each tumor type. For the post-approval era, eligible studies evaluated anti-PD-(L)1 agents approved by the FDA or EMA for each tumor type. Although FDA and EMA labels note specific monotherapy or combination therapy regimens for certain tumor types, studies evaluating any approved anti-PD-(L)1 agent delivered alone or in combination with other pharmacological agents were eligible for inclusion to ensure that a sufficiently large evidence base was captured. For both eras, no eligibility restrictions were placed on the presence or identity of comparator treatments.

#### 2.1.3 Outcomes

Eligible studies reported median OS (mOS) or OS rates at specific timepoints (i.e., landmark OS rates).

#### 2.1.4 Time

The post-approval era for each tumor type was defined as the period from the year of first anti-PD-(L)1 agent approval by the FDA or EMA for first-line therapy to the search date. The pre-approval era for each tumor type was defined as a time period beginning 5 years preceding the first FDA or EMA approval of an anti-PD-(L)1 agent for any indication (inclusive of accelerated approval) and ending the year before an anti-PD-(L)1 agent was approved for first-line therapy. The duration of this pre-approval period was selected to ensure the inclusion of a sufficiently sized evidence base while keeping a manageable scope of the SLR. Studies were categorized into pre- versus post-approval eras based on the start date of patient data collection.

#### 2.1.5 Study design

Eligible studies were observational studies employing any type of design (e.g., retrospective, prospective, or ambispective cohort or case-control studies).

### 2.2 Search strategy

Relevant studies were identified by searching Excerpta Medica database (Embase) and Medical Literature Analysis and Retrieval System Online (MEDLINE) database through the OVID portal. Separate searches were conducted for the pre- and post-approval eras for each tumor type. To specifically retrieve observational studies, search strategies employed the observational study filter from the Scottish Intercollegiate Guidelines Network (http://www.sign.ac.uk/methodology/filters.html) and filtered out irrelevant publication types, such as conference abstracts, case reports, editorials, letters, notes, and historical articles. To identify studies in the pre-approval era for each tumor type, the search strategies retrieved studies published up to 2 years after the pre-approval era ended to account for publication delays. These searches were executed on July 10, 2023 with predefined search strategies (**Supplementary Tables S1**-**S4** for advanced/metastatic melanoma, **Supplementary Tables S10**-**S13** for advanced/metastatic NSCLC, and **Supplementary Tables S20**-**S23** for advanced RCC).

### 2.3 Study selection and data extraction

Title/abstract and full-text screening against the PICOTS study selection criteria and data extraction from included studies were performed by two independent reviewers, and any discrepancies between reviewers were resolved through discussion or by involving a third reviewer. Data were extracted on study characteristics (e.g., study design, country/region, patient eligibility criteria), treatment characteristics (e.g., agents/regimens evaluated, doses), patient characteristics (e.g., age, sex, race/ethnicity, Eastern Cooperative Oncology Group [ECOG] performance status, biomarker status), and outcomes (e.g., mOS).

### 2.4 Quality assessment

The quality of included studies was assessed using the Newcastle-Ottawa Scale for Cohort Studies, which evaluates studies’ selection of study groups, comparability of study groups, and ascertainment of the outcome of interest (28). Studies with scores of 7-9 were considered good quality, 4-6 fair quality, and 0-3 poor quality. The quality assessment was performed by two independent reviewers, and any discrepancies between reviewers were resolved through discussion or by involving a third reviewer.

### 2.5 Synthesis methods

The range of mOS values was reported separately for treatment groups in the pre- and post-approval eras within each tumor type. Wherever possible, the range of mOS values as categorized by certain patient and/or treatment characteristics as appropriate for each tumor type was reported. These factors included BRAF mutation status and PD-L1 expression for melanoma; anti-PD-(L)1 therapy regimen (i.e., monotherapy or combination therapy), PD-L1 expression, and tumor histology for NSCLC; and International mRCC Database Consortium (IMDC)/Memorial Sloan Kettering Cancer Center (MSKCC) risk classification and PD-L1 expression for RCC. For RCC, IMDC risk classification was preferred when both IMDC and MSKCC risk classifications were reported, whereas MSKCC risk classification was used when IMDC classification was not reported.

## 3 Results

### 3.1 Advanced/metastatic melanoma

#### 3.1.1 Study selection

**Figure 1:**
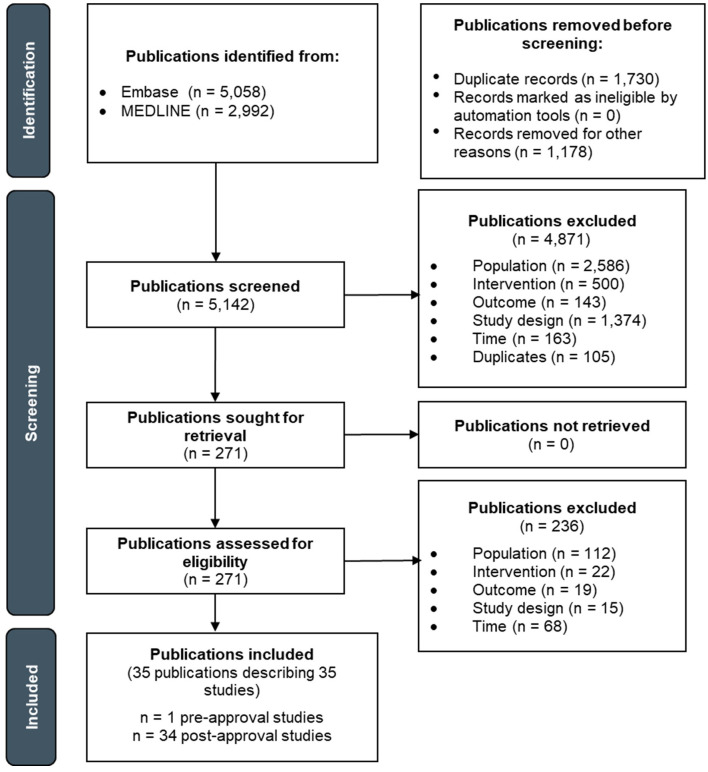
PRISMA flow diagram depicting the study selection process for advanced/metastatic melanoma.

The pre-approval era literature search yielded 4,514 publications, of which 2,812 titles/abstracts and 37 full-texts were screened (**Figure 1**). Of the publications that underwent full-text screening, 36 were excluded. The post-approval era literature search yielded 3,536 publications, of which 2,330 titles/abstracts and 234 full-texts were screened. Of the publications that underwent full-text screening, 200 were excluded. Thus, a total of 35 publications describing 35 advanced/metastatic melanoma studies were included: one study in the pre-approval era and 34 studies in the post-approval era. The study selection processes for the pre- and post-approval eras are separately depicted in **Supplementary Figures S1** and **S2**, respectively.

#### 3.1.2 Study characteristics

The one study in the pre-approval era was a retrospective cohort study conducted in France that included 50 patients whose tumors had BRAF V600E mutations (29). This study evaluated the BRAF kinase inhibitor vemurafenib.

Of the 34 studies in the post-approval era, 27 were retrospective cohort studies (30–55), six were prospective cohort studies (56–61), and one was described as an ambispective cohort study (62). These studies were conducted in European countries (n=17), the US (n = 7), Canada (n = 3), Australia (n = 3), the United Kingdom (UK) (n = 2), Canada (n = 3), Australia (n = 3), Japan (n = 1), and New Zealand (n = 1). Most studies (n = 29) did not have eligibility criteria related to BRAF mutation status. Overall, study sample size ranged from 29 to 2,322 patients. The studies evaluated anti-PD-(L)1 agents as monotherapy (n = 27), anti-PD-(L)1 agents in combination with anti-CTLA-4 (i.e., ipilimumab) or anti-RANKL (i.e., denosumab) agents (n = 14), or a mixture of treatment regimens including anti-PD-(L)1 agents as monotherapy or in combination with anti-CTLA-4 agents (n = 1).

Study characteristics for each study are presented in **Supplementary Table S5**.

#### 3.1.3 Patient characteristics

In the pre-approval era study, median patient age was 58 years, and 58% of patients were male. Most (86%) patients had an ECOG performance status of 0 or 1. Patient race/ethnicity was not reported. All patients had tumors with BRAF V600E mutations.

Across studies in the post-approval era, median/mean patient age ranged from 56 to 73 years, and the proportion of male patients ranged from 49% to 100%. The proportion of patients with an ECOG performance status of 0 or 1 ranged from 58.1% to 100%. Eight studies reported patient race/ethnicity, all of which predominantly included White patients. Regarding BRAF mutation status, the proportion of patients with V600E mutations ranged from 9.8 to 83.5%, V600K mutations ranged from 6.7% to 13.8%, and any type of V600 mutation or unspecified BRAF mutation ranged from 0% to 100%.

Patient characteristics for each study are presented in **Supplementary Table S6**.

#### 3.1.4 Outcomes

To summarize reported mOS across studies, treatment groups were categorized by BRAF mutation status. Treatment groups were not categorized by PD-L1 expression due to the sparseness of reported data.

**Figure 2:**
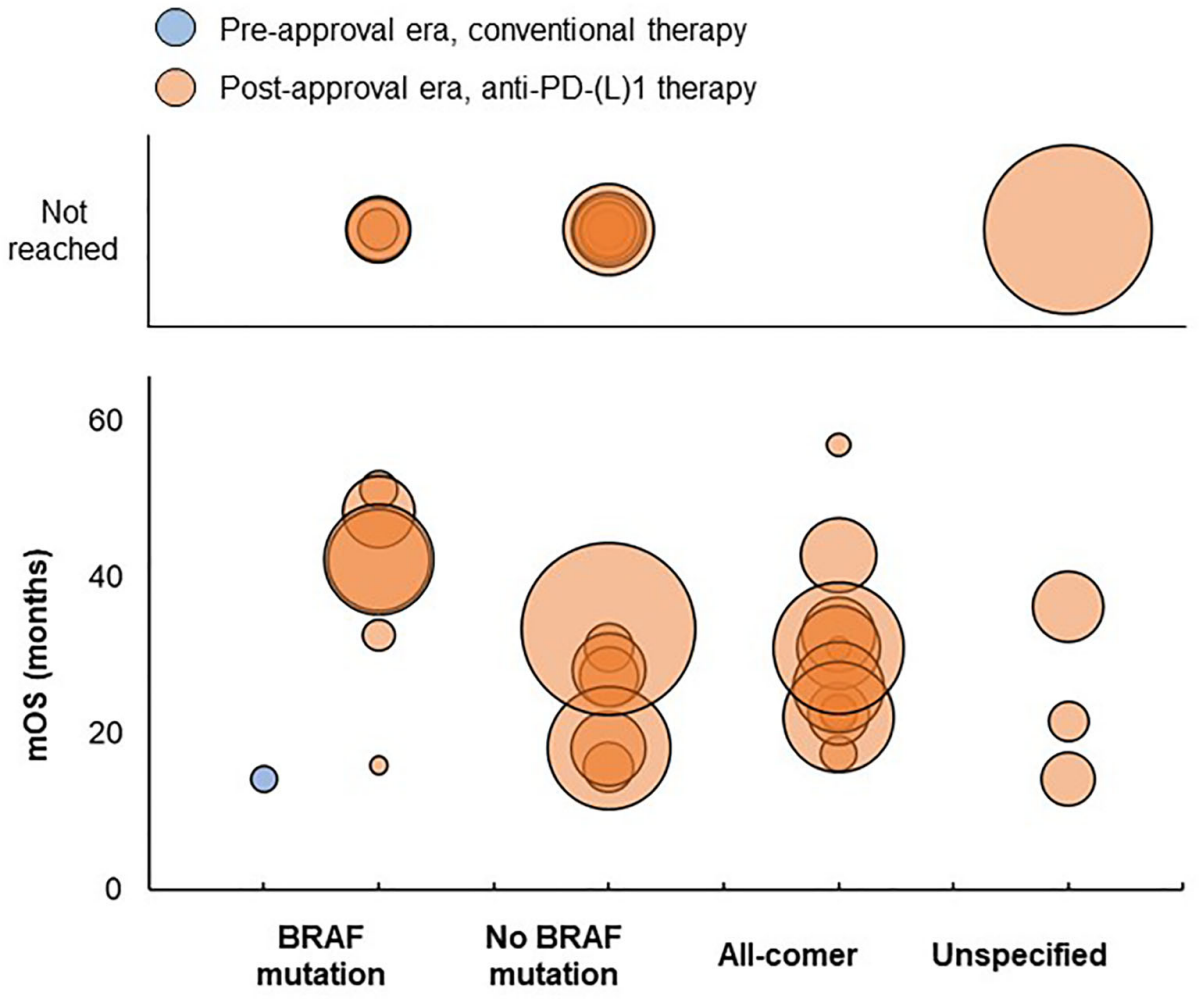
Distribution of mOS values in advanced/metastatic melanoma studies. Bubble size reflects the sample size of each treatment group, which ranged from 7 to 1,174 patients. BRAF, B-Raf proto-oncogene; mOS, median overall survival; PD-(L)1, programmed death (ligand)-1.

Considering patients with any type of BRAF mutation, mOS was 14.2 months for a treatment group receiving first-line conventional therapy in the pre-approval era and ranged from 15.9 to 51.2 months for treatment groups receiving first-line anti-PD-(L)1 therapy in the post-approval era (**Figure 2**). mOS was not reached for three treatment groups in the post-approval era; median follow-up duration was 11.3 and 23.2 months for two treatment groups, respectively, and was not reported for one treatment group.

No pre-approval era studies reported mOS for treatment groups of patients without BRAF mutations, all-comer patients, or patients with unspecified BRAF mutation status. Post-approval era studies reported an mOS ranging from 15.7 to 33.4 months for treatment groups of patients without BRAF mutations, from 17.4 to 57 months for treatment groups of all-comer patients, and from 14.2 to 36.2 months for treatment groups of patients with unspecified BRAF mutation status. mOS was not reached for six treatment groups without BRAF mutations in the post-approval era; median follow-up duration was 12.1, 14.5, and 16.5 months for three treatment groups, respectively, and was not reported for three treatment groups. mOS was not reached for one treatment group of patients with unspecified BRAF mutation status in the post-approval era, which had a median follow-up duration of 25 months.

A summary and detailed information on median follow-up durations, mOS, and landmark OS rates for each study are presented in **Supplementary Tables S7** and **S8**.

#### 3.1.5 Study quality

All studies were judged to be of fair quality in both the pre- and post-approval eras, largely due to the absence of a non-exposed cohort and some concerns regarding the representativeness of the exposed cohort and length of follow-up (**Supplementary Table S9**).

### 3.2 Advanced/metastatic NSCLC

#### 3.2.1 Study selection

**Figure 3:**
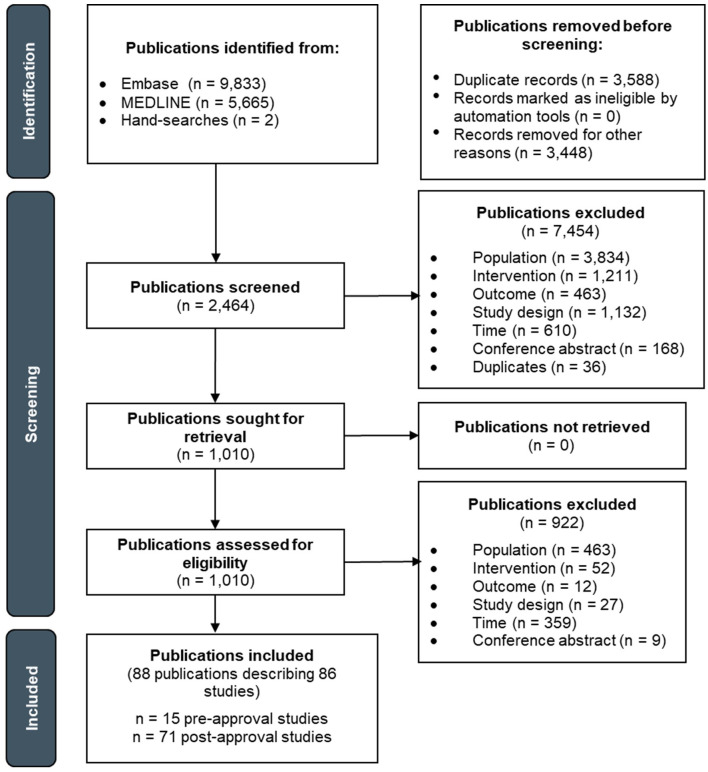
PRISMA flow diagram depicting the study selection process for advanced/metastatic NSCLC.

The pre-approval era literature search yielded 10,797 publications, of which 5,583 titles/abstracts and 495 full-texts were screened (**Figure 3**). Of the publications that underwent full-text screening, 480 were excluded. The post-approval era literature search yielded 4,703 publications, of which 2,881 titles/abstracts and 515 full-texts were screened. Of the publications that underwent full-text screening, 442 were excluded. Thus, a total of 88 publications describing 86 advanced/metastatic NSCLC studies were included: 15 studies in the pre-approval era and 71 studies in the post-approval era. The study selection processes for the pre- and post-approval eras are separately depicted in **Supplementary Figures S3** and **S4**, respectively.

#### 3.2.2 Study characteristics

Of the 15 studies in the pre-approval era, 13 were retrospective cohort studies (63–75), and two were prospective cohort studies (76, 77). These studies were conducted in European countries (n = 6), China (n = 3), the US (n = 2), Saudi Arabia (n = 1), Japan (n = 1), India (n = 1), or multiple countries around the world (n = 1). Overall study sample size ranged from 20 to 2,014 patients. The studies evaluated chemotherapy regimens containing platinum, taxanes, or other chemotherapeutic agents (n = 7); vascular endothelial growth factor (VEGF)-targeting therapy (i.e., bevacizumab) in combination with chemotherapy (n = 4); epidermal growth factor receptor (EGFR)-targeting therapy (i.e., icotinib or erlotinib) (n = 3); or a mixture of treatment regimens including chemotherapy and/or unnamed tyrosine kinase inhibitors (TKIs) (n = 3).

Of the 71 studies in the post-approval era, 68 were retrospective cohort studies (78–147), and three were prospective cohort studies (148–150). These studies were conducted in European countries (n = 26), Japan (n = 13), the US (n = 11), China/Taiwan (n = 9), Israel (n = 4), the UK and European countries (n = 3), Australia (n = 2), Canada (n = 1), Singapore (n = 1), or multiple countries across North and South America (n = 1). Overall study sample size ranged from 30 to 7,312 patients. The studies evaluated anti-PD-(L)1 agents as monotherapy (n = 52), anti-PD-(L)1 agents in combination with chemotherapy and/or anti-VEGF therapy (n = 21), or a mixture of treatment regimens including anti-PD-(L)1 agents as monotherapy or in combination with chemotherapy (n = 6).

Study characteristics for each study are presented in **Supplementary Table S14**.

#### 3.2.3 Patient characteristics

Across studies in the pre-approval era, median/mean patient age ranged from 60.2 to 71 years, and the proportion of male patients ranged from 37.5% to 90%. The proportion of patients with an ECOG performance status of 0 or 1 ranged from 28% to 100%. Two studies reported patient race/ethnicity: one predominantly included White patients, and one was a global study in which patients recruited in Italy, Spain, Germany, Australia, and Brazil were predominantly White and patients recruited in the Republic of Korea and Taiwan were predominantly Asian. Among studies that reported smoking status, the proportion of patients who were current or former smokers ranged from 32.7% to 97%. Seven studies included both patients with squamous and non-squamous tumors, four exclusively included patients with non-squamous tumors, two exclusively included patients with squamous tumors, and two did not report on patients’ tumor histology. The proportion of patients with EGFR or anaplastic lymphoma kinase (ALK) aberrations ranged from 0% to 100%. No studies reported on PD-L1 expression.

Across studies in the post-approval era, median/mean patient age ranged from 59.8 to 74 years, and the proportion of male patients ranged from 35.3% to 93.9%. The proportion of patients with an ECOG performance status of 0 or 1 ranged from 46% to 100%. Thirteen studies reported patient race/ethnicity: 12 predominantly included White patients, and one predominantly included Hispanic patients. Among studies that reported smoking status, the proportion of patients who were current or former smokers ranged from 50% to 99%. Fifty-six studies included both patients with squamous and non-squamous tumors, 11 exclusively included patients with non-squamous tumors, one exclusively included patients with squamous tumors, and three did not report on patients’ tumor histology. The proportion of patients with EGFR or ALK aberrations ranged from 0% to 23.3%. Several studies reported on PD-L1 expression; the proportion of patients with a PD-L1 tumor proportion score (TPS) ≥1% ranged from 20.6% to 100%, TPS 1-49% ranged from 0% to 67.3%, and TPS ≥50% ranged from 11.8% to 100%.

Patient characteristics for each study are presented in **Supplementary Tables S15** and **S16**.

#### 3.2.4 Outcomes

To summarize reported mOS across studies, treatment groups receiving anti-PD-(L)1 monotherapy in the post-approval era were categorized by tumor PD-L1 expression. However, as PD-L1 expression was not reported for treatment groups in the pre-approval era (because PD-L1 expression was not of relevance to treatment decisions at that time), pre-approval treatment groups were not categorized by this factor. All treatment groups had to consist of ≥80% of patients with no tumor EGFR/ALK aberrations to be included in the mOS summary to align as closely as possible with current FDA/EMA-approved indications for anti-PD-(L)1 therapy.

**Figure 4:**
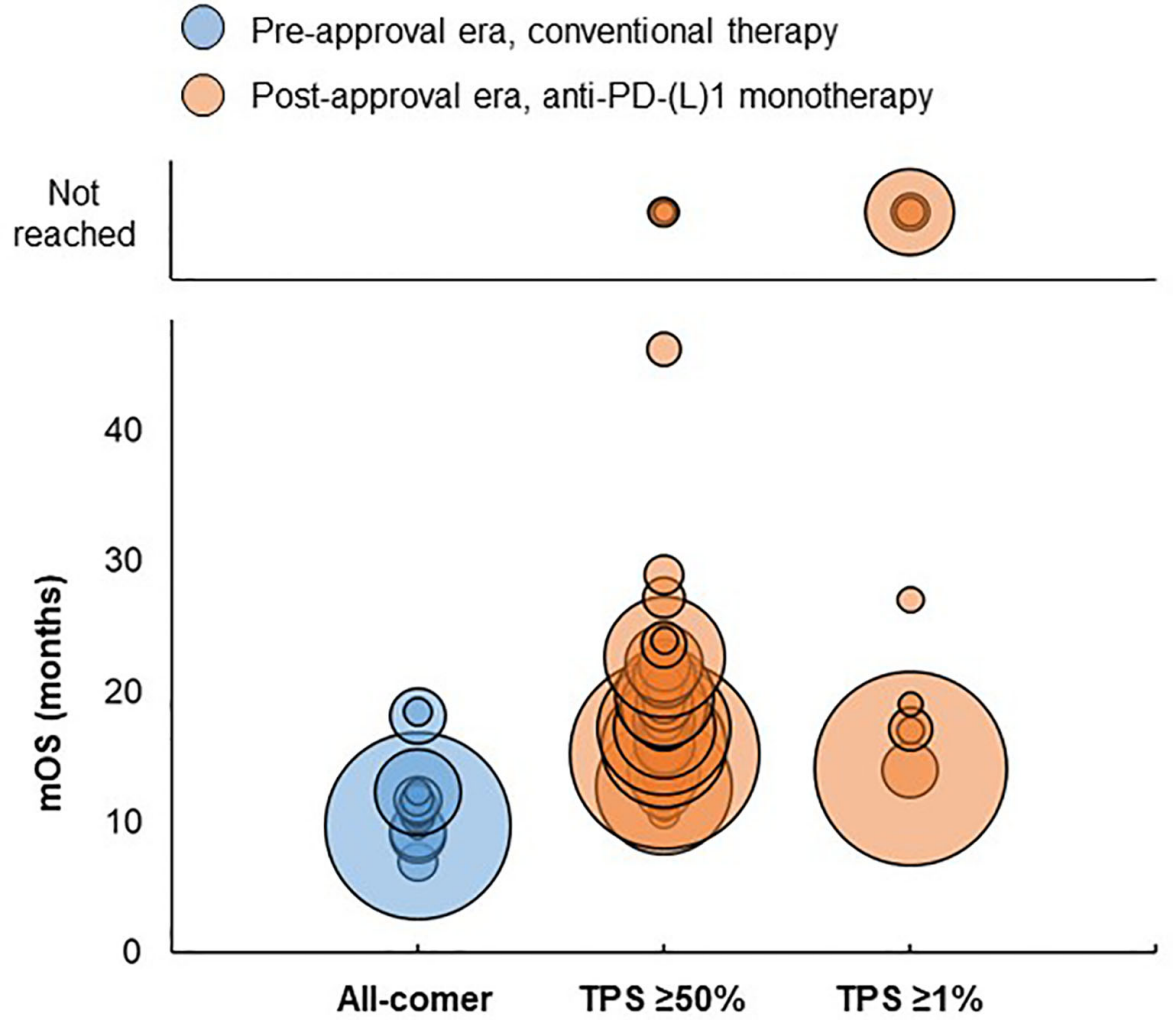
Distribution of mOS values by PD-L1 expression in advanced/metastatic NSCLC studies. Bubble size reflects the sample size of each treatment group, which ranged from 5 to 2,166 patients. TPS, PD-L1 tumor proportion score.

In the pre-approval era, mOS ranged from 6.9 to 18.4 months across treatment groups of all-comer patients receiving first-line conventional therapy (**Figure 4**). In the post-approval era, mOS ranged from 10.6 to 46.2 months for treatment groups with PD-L1 TPS ≥50% and from 14 to 27 months for treatment groups with PD-L1 TPS ≥1% receiving first-line anti-PD-(L)1 monotherapy. mOS was not reached three treatment groups with PD-L1 TPS ≥50%; median follow-up durations were 19.9 and 26.5 months for two treatment groups, respectively, and was not reported for one treatment group. mOS was not reached for three treatment groups with PD-L1 TPS ≥1%, which had median follow-up durations of 11.3, 12.5, and 14.5 months, respectively.

Next, treatment groups receiving conventional therapy in the pre-approval era and anti-PD(L)1 combination therapy in the post-approval era were categorized by tumor histology. For all categories except the ‘100% squamous’ category, treatment groups had to consist of ≥80% of patients with no tumor EGFR/ALK aberrations to be included in the mOS summary to align as closely as possible with current FDA/EMA-approved indications for anti-PD-(L)1 therapy.

**Figure 5:**
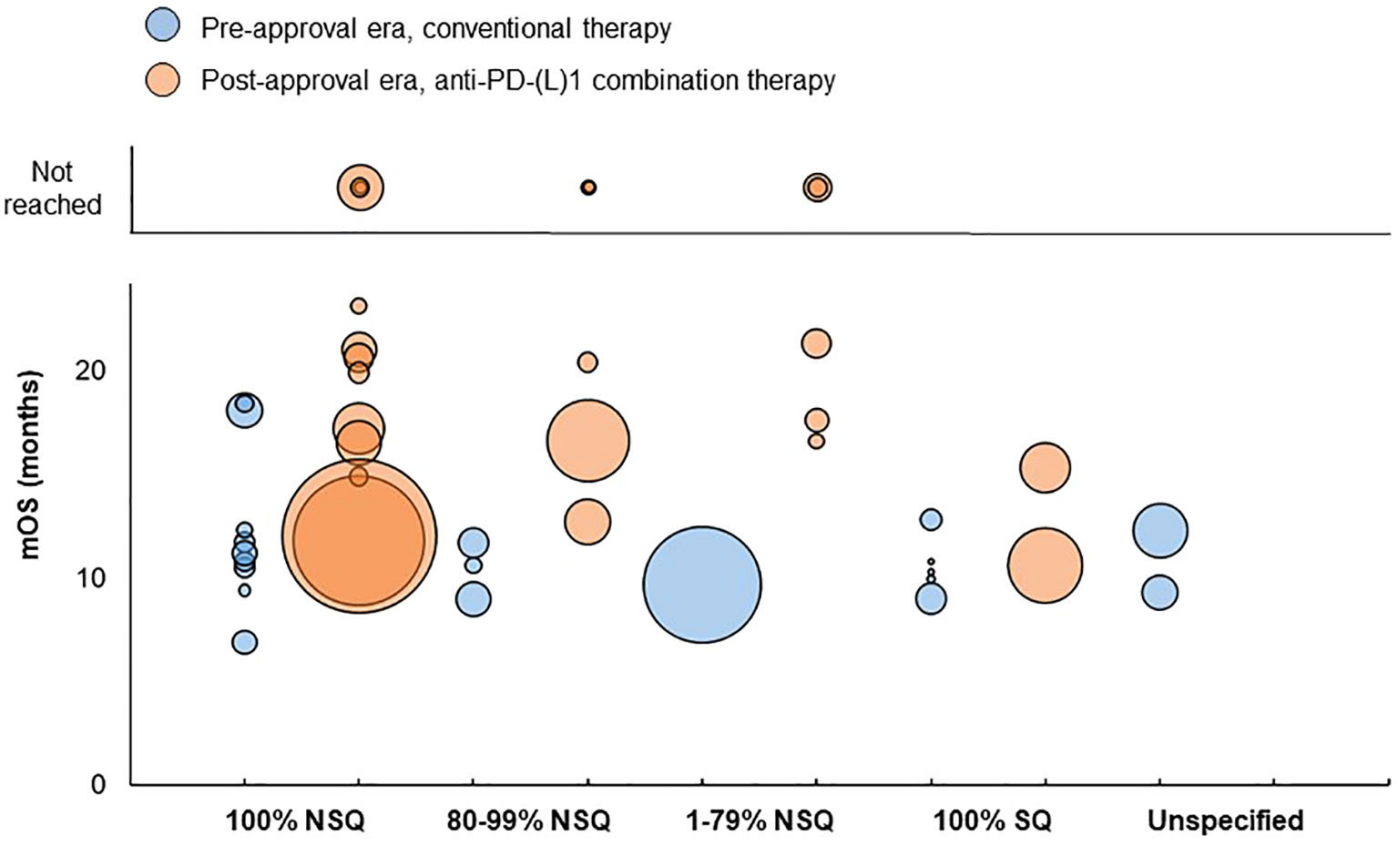
Distribution of mOS values by tumor histology in advanced/metastatic NSCLC studies. Bubble size reflects the sample size of each treatment group, which ranged from 5 to 2,488 patients. Sample size was not reported for two ‘100% SQ’ treatment groups in the pre-approval era, which had a mOS of 17 months and 17.6 months respectively; these treatment groups are not reflected in the chart. mOS, median overall survival; NSQ, non-squamous; PD-L1, programmed death (ligand)-1; SQ, squamous.

For treatment groups consisting of 100% non-squamous patients, mOS ranged from 6.9 to 18.4 months in the pre-approval era and from 11.8 to 23.1 months in the post-approval era (**Figure 5**). mOS was not reached for three treatment groups in the post-approval era, which had median follow-up durations of 5.5, 8, and 10.3 months, respectively.

For treatment groups consisting of 80-99% non-squamous patients, mOS ranged from 9 to 11.7 months in the pre-approval era and from 12.7 to 20.4 months in the post-approval era. mOS was not reached for two treatment groups in the post-approval era, both of which had median follow-up durations of 14.8 months.

For treatment groups consisting of 1-79% non-squamous patients, mOS was 9.67 months in the pre-approval era and ranged from 16.6 to 21.3 months in the post-approval era. mOS was not reached for two treatment groups in the post-approval era, which had median follow-up durations of 8.9 and 17.13 months, respectively.

For treatment groups consisting of 100% squamous patients, mOS ranged from 9 to 12.8 months in the pre-approval era and from 10.6 to 15.3 in the post-approval era.

For treatment groups consisting of patients with unspecified tumor histology, mOS ranged from 9.3 to 12.3 months in the pre-approval era and was not reported in the post-approval era.

A summary and detailed information on median follow-up durations, mOS, and landmark OS rates are provided in **Supplementary Tables S17** and **S18**.

#### 3.2.5 Study quality

All studies were judged to be of fair quality in both the pre- and post-approval eras, largely due to the absence of a non-exposed cohort and some concerns regarding the representativeness of the exposed cohort, ascertainment of exposure, assessment of outcome, and length of follow-up (**Supplementary Table S19**).

### 3.3 Advanced RCC

#### 3.3.1 Study selection

**Figure 6:**
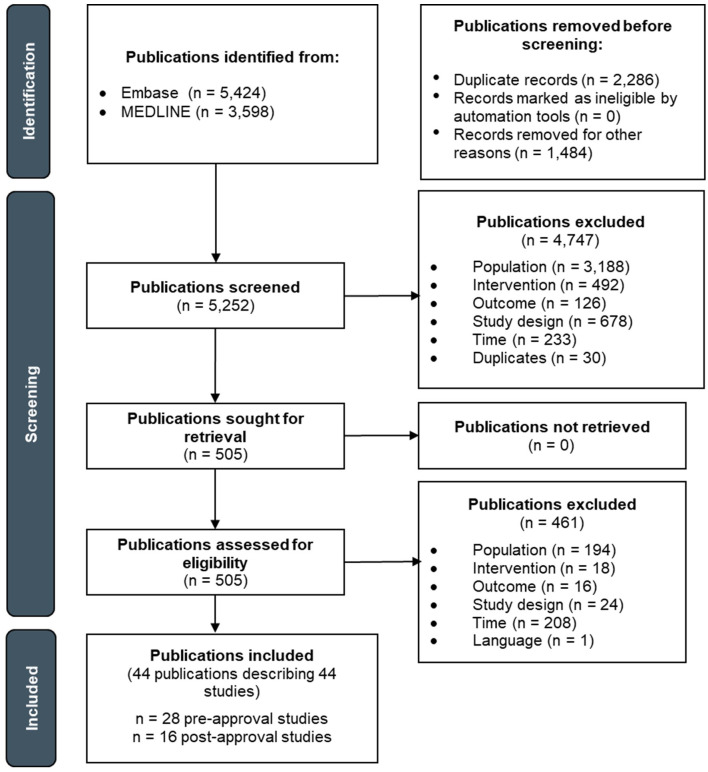
PRISMA flow diagram depicting the study selection process for advanced RCC.

The pre-approval era literature search yielded 7,517 publications, of which 4,323 titles/abstracts and 310 full-texts were screened (**Figure 6**). Of the publications that underwent full-text screening, 282 were excluded. The post-approval era literature search yielded 1,505 publications, of which 929 titles/abstracts and 195 full-texts were screened. Of the publications that underwent full-text screening, 179 were excluded. Thus, a total of 44 publications describing 44 advanced RCC studies were included: 28 studies in the pre-approval era and 16 studies in the post-approval era. The study selection processes for the pre- and post-approval eras are separately depicted in **Supplementary Figures S5** and **S6**, respectively.

#### 3.3.2 Study characteristics

Of the 28 studies in the pre-approval era, 21 were retrospective cohort studies (151–171), and seven were prospective cohort studies (172–178). These studies were conducted in European countries (n = 9), India (n = 5), Egypt (n = 3), China (n = 2), Japan (n = 2), the Republic of Korea (n = 2), Australia (n = 1), Brazil (n = 1), Turkey (n = 1), multiple countries across North and South America (n = 1), or multiple countries around the world (n = 1). Overall study sample size ranged from 15 to 657 patients. The studies evaluated multi-targeted receptor tyrosine kinase (RTK) inhibitor (i.e., sunitinib, pazopanib, sorafenib) monotherapy (n = 27), multi-targeted RTK inhibitor (i.e., sorafenib) in combination with interferon therapy (n = 1), or interferon monotherapy (n = 1).

Of the 16 studies in the post-approval era, 13 were retrospective cohort studies (179–191), and three were prospective cohort studies (192–194). These studies were conducted in Japan (n = 9), the Republic of Korea (n = 3), the US (n = 3), or Italy (n = 1). Overall study sample size ranged from 35 to 1,538 patients. The studies evaluated nivolumab in combination with an anti-CTLA-4 agent (i.e., ipilimumab) (n = 10), pembrolizumab in combination with a TKI (i.e., axitinib) (n = 5), or mixtures of treatment regimens including anti-PD-(L)1 agents in combination with anti-CTLA-4 agents or TKIs (n = 3).

Study characteristics for each study are presented in **Supplementary Table S24**.

#### 3.3.3 Patient characteristics

Across studies in the pre-approval era, median/mean patient age ranged from 49 to 69 years, and the proportion of male patients ranged from 22.2% to 83.6%. The proportion of patients with an ECOG performance status of 0 or 1 ranged from 35% to 100%. Three studies reported patient race/ethnicity; one study included exclusively Asian patients, one study included mostly White patients, and one study included mostly Hispanic patients. Considering IMDC/MSKCC risk classification, 3% to 56% of patients were classified as poor risk, 93% of patients were classified as poor/intermediate risk, 29% to 100% of patients were classified as intermediate risk, and 3.7% to 65% of patients were classified as favorable risk across studies.

Across studies in the post-approval era, median/mean patient age ranged from 58 to 71 years, and the proportion of male patients ranged from 68.8% to 84.8%. The proportion of patients with an ECOG performance status of 0 or 1 ranged from 50.9% to 88.4%. Three studies reported patient race/ethnicity, all of which included mostly White patients. Considering IMDC/MSKCC risk classification, 21.4% to 100% of patients were classified as poor risk, 85.7% to 93.6% of patients were classified as poor/intermediate risk, 0% to 69.4% of patients were classified as intermediate risk, 5.3% to 11.8% of patients were classified as favorable/intermediate risk, and 0% to 37.9% of patients were classified as favorable risk across studies.

Patient characteristics for each study are presented in **Supplementary Tables S25** and **S26**.

#### 3.3.4 Outcomes

To summarize reported mOS across studies, treatment groups were categorized by IMDC/MSKCC risk classification. Treatment groups were not categorized by PD-L1 expression due to the sparseness of reported data.

**Figure 7:**
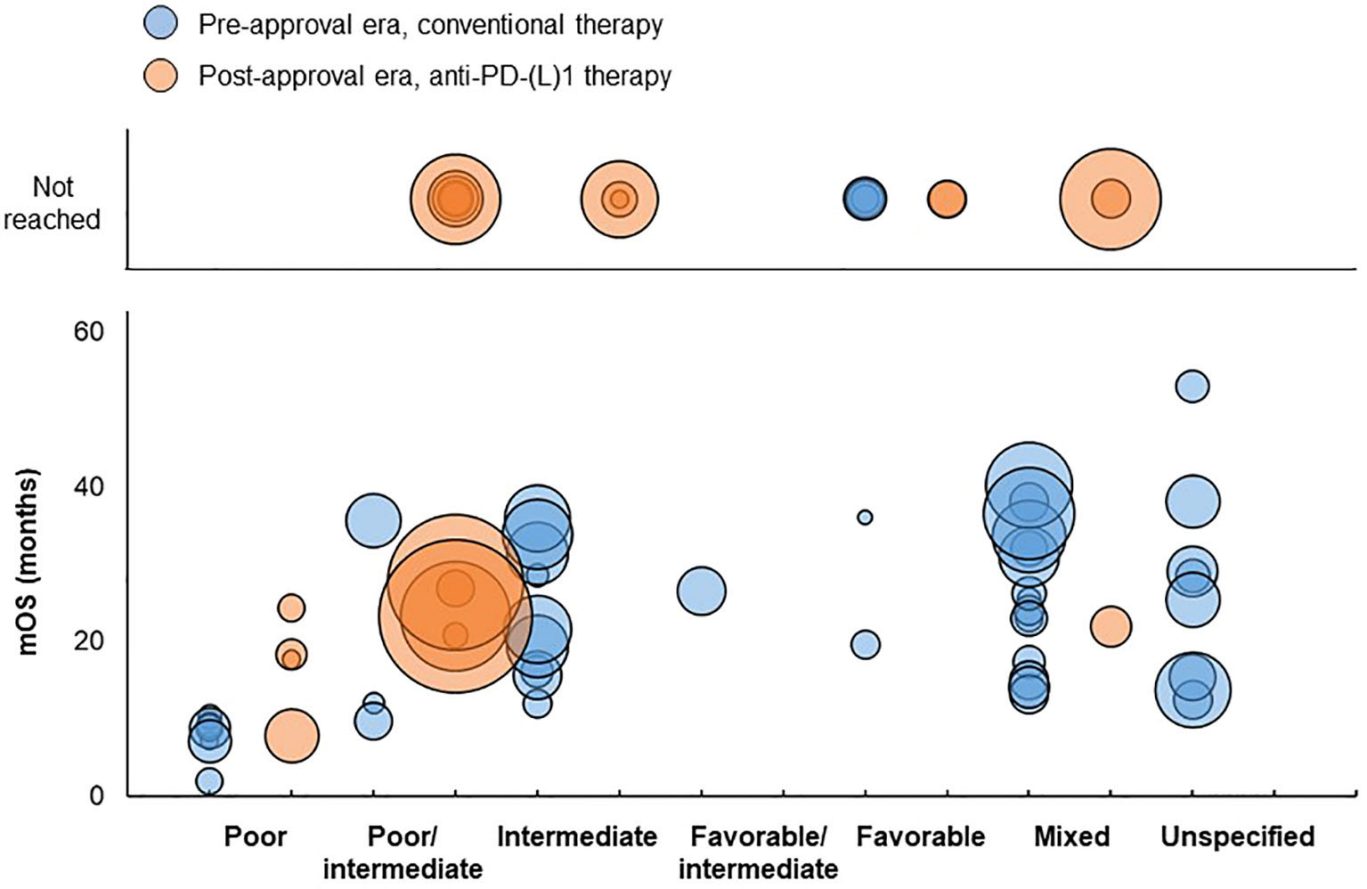
Distribution of mOS values in advanced RCC studies. Bubble size reflects the sample size of each treatment group, which ranged from 7 to 817 patients. Inter, intermediate; mOS, median overall survival; PD-(L)1, programmed death (ligand)-1.

For patients classified as having poor risk, mOS ranged from 2 to 10.3 months for treatment groups receiving first-line conventional therapy in the pre-approval era and from 7.8 to 24.3 months for treatment groups receiving first-line anti-PD-(L)1 therapy in the post-approval era (**Figure 7**).

For patients classified as having poor/intermediate risk, mOS ranged from 9.7 to 35.6 months in the pre-approval era and from 20.8 to 27.6 months in the post-approval era. mOS was not reached for five additional treatment groups in the post-approval era, which had median follow-up durations of 7.2, 12, 12.4, 13.8, and 16.1 months, respectively.

For patients classified as having intermediate risk, mOS ranged from 12 to 35.9 months in the pre-approval era and was not reached in all three treatment groups in the post-approval era, which had median follow-up durations of 8.96, 12.2, and 24 months, respectively.

For patients classified as having favorable/intermediate risk, mOS was 26.5 months in the pre-approval era and was not reported in the post-approval era.

For patients classified as having favorable risk, mOS ranged from 19.6 to 36 months in the pre-approval era. mOS was not reached for an additional four treatment groups in the pre-approval era; two of these treatment groups had median follow-up durations of 23 and 46.1 months, respectively, and two treatment groups did not report median follow-up duration. mOS was not reached for either treatment group in the post-approval era, both of which had median follow-up durations of 20 months.

For treatment groups consisting of patients with mixed risk classifications, mOS ranged from 13.2 to 40.2 months in the pre-approval era and was 21.9 months in the post-approval era. mOS was not reached for two treatment groups in the post-approval era, which had median follow-up durations of 7 and 9.67 months, respectively.

For treatment groups consisting of patients with unspecified risk classification, mOS ranged from 12.5 to 52.97 months in the pre-approval era and was not reported in the post-approval era.

A summary and detailed information on median follow-up durations, mOS, and landmark OS rates are provided in **Supplementary Tables S27** and **S28**.

#### 3.3.5 Study quality

Most studies were judged to be of fair quality in both the pre- and post-approval eras, largely due to the absence of a non-exposed cohort and some concerns regarding the representativeness of the exposed cohort, demonstration that the outcome of interest was not present at the start of the study, length of follow-up, and adequacy of follow-up of cohorts (**Supplementary Table S29**).

## 4 Discussion

The development and regulatory approval of ICIs, including anti-PD-(L)1 agents, has dramatically changed the way cancer is treated in clinical practice. However, as the regulatory approval of cancer treatments is largely based on the findings of RCTs, it is unclear whether treatment efficacy as measured in these tightly controlled trials translates to real-world settings. Therefore, the objective of this SLR was to understand the real-world OS of patients with advanced/metastatic melanoma, NSCLC, or RCC who would currently be eligible, based on their clinical presentation, for treatment with an anti-PD-(L)1 agent but were treated with conventional care before the regulatory approval of anti-PD-(L)1 therapy and of patients treated with anti-PD-(L)1 agents after their approval.

The results of this SLR show that mOS values in the post-approval era tended to be longer than those in the pre-approval era for patients with advanced/metastatic NSCLC in the first-line setting, including NSCLC patients with PD-L1-positive tumors who received anti-PD-(L)1 monotherapy and NSCLC patients with non-squamous cell carcinoma who received anti-PD-(L)1 combination therapy. Notably, the ranges in mOS values for PD-L(1)-treated NSCLC patients observed in this SLR are similar to those reported in previous SLRs and meta-analyses of real-world studies evaluating ICI therapies in NSCLC patients (9–11, 195), including a meta-analysis demonstrating similar efficacy/effectiveness of ICIs in terms of mOS between RCTs and real-world studies (12). In addition, mOS tended to be longer in the post-approval era than the pre-approval era for advanced/metastatic melanoma patients with BRAF mutations and high-risk advanced RCC patients.

Conclusions drawn from this study may be limited by some considerations. First, there were imbalances in the numbers of pre- versus post-approval studies for each tumor type. This was especially pronounced for advanced/metastatic melanoma, for which only one pre-approval study was identified. As this study evaluated a targeted agent (i.e., vemurafenib) in patients with BRAF V600E mutations, the OS of patients in this study may not be representative of the OS of general advanced/metastatic melanoma patients who received conventional therapy before the approval of PD-(L)1 therapy. Second, while the aim of this study was to provide a global view of real-world OS before and after the approval of PD-(L)1 therapy, there were some imbalances in the geographical locations of studies in the pre- and post-approval eras for certain tumor types. Third, as measurements of OS are influenced by all therapies received by patients throughout their disease course, the mOS values reported by observational studies may have been impacted by other therapies received by patients (including subsequent lines of therapy) that were not a focus of or described by the studies. Fourth, most included studies were only of fair quality due to their lack of detailed reporting on study methodology, leading to concerns about the potential existence of bias in patient selection and outcome assessment. Fifth, mOS was reported as ‘not reached’ for some treatment groups. Thus, there is uncertainty as to whether this reflects the prolonged survival of patients or an insufficient study follow-up duration, as many studies did not report follow-up durations. Notably, mOS tended to be more frequently reported as ‘not reached’ in post-approval era studies, which may have served to artificially truncate the upper bound of the range in mOS values and thereby conceal a potential survival benefit in the post-approval era. Sixth, there was substantial heterogeneity among included studies in study objectives, patient characteristics, and evaluated treatment regimens, which may have contributed to between-study variation in OS. Future research involving a formal quantitative analysis such as a meta-regression to control for potential sources of variance in OS outcomes and determine the statistical significance of differences in pre- vs. post-approval OS could help overcome this limitation. Finally, this SLR was limited by the availability of published data, with an inherent potential risk of publication bias.

Despite these limitations, this SLR also has strengths that maximize its comprehensiveness and rigor. It involved highly sensitive searches of the peer-reviewed literature, the review process was guided by pre-defined eligibility criteria established in a protocol, and data quality was ensured through the involvement of two reviewers in the study selection and data extraction phases. Moreover, rather than focusing on ICIs in general, the present study investigated the survival of similar populations of cancer patients before versus after the approval of anti-PD-(L)1 therapies in particular. Thus, this SLR generated a large, unique evidence base encompassing real-world evidence on the treatment of advanced/metastatic melanoma, NSCLC, and RCC that enriches our understanding of the degree of value and improvements in outcomes that anti-PD-(L)1 therapy has brought to cancer patients in actual clinical practice.

## 5 Conclusion

In conclusion, a survival benefit in real-world practice was observed for patients with advanced/metastatic NSCLC, RCC, or melanoma receiving first-line anti-PD-(L)1 therapy after its regulatory approval when compared with patients treated with conventional care before anti-PD-(L)1 therapy approval. This supports the use of anti-PD-(L)1 therapy as a standard of care in many countries.

## Data Availability

The original contributions presented in the study are included in the article/**Supplementary Material**. Further inquiries can be directed to the corresponding author.
