# Supplementary material for: Systematic literature review of real-world evidence on overall survival in cancer patients before and after the approval of anti-PD-(L)1 therapy
Source: Front Oncol. 2025 Aug 4;15:1615795. doi: 10.3389/fonc.2025.1615795 (PMC12358273; doi:10.3389/fonc.2025.1615795)
Supplement: Supplementary file 1 [file DataSheet1.docx]

Supplementary Material

# Advanced/Metastatic Melanoma

## Literature Search Strategies

Table S1: Advanced/metastatic melanoma, pre-approval era: Embase search strategy

*Database: Embase <1974 to 2023 July 7>; Search date: July 10, 2023*

| **Line** | **Search terms** | **Hits** |
| --- | --- | --- |
| 1 | exp skin tumor/ or exp skin neoplasms/ or exp melanoma/ | 393585 |
| 2 | (((skin) adj3 (neoplasm$ or cancer$ or tumo?r* or carcinoma$ or adenocarcinoma$ or sarcoma$)) or melanoma).mp. | 330250 |
| 3 | or/1-2 | 450268 |
| 4 | exp metastasis/ or exp neoplasm metastasis/ or (advance$ or metasta$ or recurr$ or unresect$ or non-resect$ or disseminated or stage 3 or stage III* or stage 4 or stage IV* or spread$ or migration$ or progress$ or invasive or aggressive or "not operable" or untreatable or "not treatable" or secondary or incurable or "not curable").mp. | 7409869 |
| 5 | 3 and 4 | 215285 |
| 6 | exp overall survival/ or exp survival rate/ or exp life expectancy/ or exp survival analysis/ or (surviv* or OS or mortality or death* or die* or life expectancy).mp. | 6478513 |
| 7 | Clinical study/ | 163725 |
| 8 | Case control study/ | 207756 |
| 9 | Family study/ | 25786 |
| 10 | Longitudinal study/ | 196031 |
| 11 | Retrospective study/ | 1490609 |
| 12 | Prospective study/ | 886615 |
| 13 | Randomized controlled trials/ | 264163 |
| 14 | 12 not 13 | 875476 |
| 15 | Prospective study/ | 886615 |
| 16 | Cohort analysis/ | 1056494 |
| 17 | (Cohort adj (study or studies)).mp. | 485180 |
| 18 | (Case control adj (study or studies)).tw. | 170347 |
| 19 | (follow up adj (study or studies)).tw. | 74216 |
| 20 | (observational adj (study or studies)).tw. | 257702 |
| 21 | (epidemiologic$ adj (study or studies)).tw. | 123233 |
| 22 | (cross sectional adj (study or studies)).tw. | 343578 |
| 23 | or/7-11, 14-22 | 3974056 |
| 24 | case study/ | 99618 |
| 25 | case report/ or case report.tw. | 2963471 |
| 26 | conference abstract/ or conference paper/ or conference review/ | 2711315 |
| 27 | editorial/ | 751065 |
| 28 | letter/ | 1234492 |
| 29 | note/ | 886897 |
| 30 | review/ or short survey/ | 3514301 |
| 31 | or/24-30 | 11437910 |
| 32 | 23 not 31 | 3058593 |
| 33 | 5 and 6 and 32 | 12163 |
| 34 | limit 33 to yr=2009 - 2015 | 2690 |
| 35 | limit 34 to english | 2588 |

Table S2: Advanced/metastatic melanoma, pre-approval era: MEDLINE search strategy

*Database: Ovid MEDLINE(R) and Epub Ahead of Print, In-Process, In-Data-Review & Other Non-Indexed Citations and Daily 1946 to July 06, 2023; Search date: July 10, 2023*

| **Line** | **Search terms** | **Hits** |
| --- | --- | --- |
| 1 | exp skin tumor/ or exp skin neoplasms/ or exp melanoma/ | 208384 |
| 2 | (((skin) adj3 (neoplasm$ or cancer$ or tumo?r* or carcinoma$ or adenocarcinoma$ or sarcoma$)) or melanoma).mp. | 259840 |
| 3 | or/1-2 | 265555 |
| 4 | exp metastasis/ or exp neoplasm metastasis/ or (advance$ or metasta$ or recurr$ or unresect$ or non-resect$ or disseminated or stage 3 or stage III* or stage 4 or stage IV* or spread$ or migration$ or progress$ or invasive or aggressive or "not operable" or untreatable or "not treatable" or secondary or incurable or "not curable").mp. | 5285610 |
| 5 | 3 and 4 | 111940 |
| 6 | exp overall survival/ or exp survival rate/ or exp life expectancy/ or exp survival analysis/ or (surviv* or OS or mortality or death* or die* or life expectancy).mp. | 4656980 |
| 7 | Epidemiologic studies/ | 9353 |
| 8 | Exp case control studies/ | 1428358 |
| 9 | Exp cohort studies/ | 2498788 |
| 10 | Case control.tw. | 154046 |
| 11 | (cohort adj (study or studies)).tw. | 316754 |
| 12 | Cohort analy$.tw. | 11810 |
| 13 | (Follow up adj (study or studies)).tw. | 56299 |
| 14 | (observational adj (study or studies)).tw. | 161394 |
| 15 | Longitudinal.tw. | 322757 |
| 16 | Retrospective.tw. | 745567 |
| 17 | Cross sectional.tw. | 513409 |
| 18 | Cross-sectional studies/ | 471331 |
| 19 | or/7-18 | 3796175 |
| 20 | case reports/ or case report.tw. | 2404681 |
| 21 | editorial/ | 656410 |
| 22 | historical article/ | 369352 |
| 23 | letter/ | 1222167 |
| 24 | review/ or systematic review/ or meta-analysis/ | 3343956 |
| 25 | or/20-24 | 7556164 |
| 26 | 19 not 25 | 3427154 |
| 27 | 5 and 6 and 26 | 8924 |
| 28 | limit 27 to yr=2009 - 2015 | 2019 |
| 29 | limit 28 to english | 1926 |

Table S3: Advanced/metastatic melanoma, post-approval era: Embase search strategy

*Database: Embase <1974 to 2023 July 7>; Search date: July 10, 2023*

| **Line** | **Search terms** | **Hits** |
| --- | --- | --- |
| 1 | exp skin tumor/ or exp skin neoplasms/ or exp melanoma/ | 393585 |
| 2 | (((skin) adj3 (neoplasm$ or cancer$ or tumo?r* or carcinoma$ or adenocarcinoma$ or sarcoma$)) or melanoma).mp. | 330250 |
| 3 | or/1-2 | 450268 |
| 4 | exp metastasis/ or exp neoplasm metastasis/ or (advance$ or metasta$ or recurr$ or unresect$ or non-resect$ or disseminated or stage 3 or stage III* or stage 4 or stage IV* or spread$ or migration$ or progress$ or invasive or aggressive or "not operable" or untreatable or "not treatable" or secondary or incurable or "not curable").mp. | 7409869 |
| 5 | 3 and 4 | 215285 |
| 6 | exp atezolizumab/ or (atezolizumab or MPDL-3280A or MPDL3280A or RG7446 or RG-7446 or tecentriq).mp. | 15638 |
| 7 | exp nivolumab/ or (nivolumab or opdivo or ONO-4538 or BMS-936558 or MDX1106).mp. | 38765 |
| 8 | exp pembrolizumab/ or (pembrolizumab or MK-3475 or SCH-900475 or lambrolizumab or keytruda).mp. | 37947 |
| 9 | exp immune checkpoint inhibitors/ or (checkpoint or ICI* or (PD1 or PD-1 or PDL1 or PD-L1) and (inhibit*)).mp. | 141080 |
| 10 | or/6-9 | 173820 |
| 11 | exp overall survival/ or exp survival rate/ or exp life expectancy/ or exp survival analysis/ or (surviv* or OS or mortality or death* or die* or life expectancy).mp. | 6478513 |
| 12 | Clinical study/ | 163725 |
| 13 | Case control study/ | 207756 |
| 14 | Family study/ | 25786 |
| 15 | Longitudinal study/ | 196031 |
| 16 | Retrospective study/ | 1490609 |
| 17 | Prospective study/ | 886615 |
| 18 | Randomized controlled trials/ | 264163 |
| 19 | 19 not 20 | 1336794 |
| 20 | Prospective study/ | 886615 |
| 21 | Cohort analysis/ | 1056494 |
| 22 | (Cohort adj (study or studies)).mp. | 485180 |
| 23 | (Case control adj (study or studies)).tw. | 170347 |
| 24 | (follow up adj (study or studies)).tw. | 74216 |
| 25 | (observational adj (study or studies)).tw. | 257702 |
| 26 | (epidemiologic$ adj (study or studies)).tw. | 123233 |
| 27 | (cross sectional adj (study or studies)).tw. | 343578 |
| 28 | or/12-16, 19-27 | 4987971 |
| 29 | case study/ | 99618 |
| 30 | case report/ or case report.tw. | 2963471 |
| 31 | conference abstract/ or conference paper/ or conference review/ | 2711315 |
| 32 | editorial/ | 751065 |
| 33 | letter/ | 1234492 |
| 34 | note/ | 886897 |
| 35 | review/ or short survey/ | 3514301 |
| 36 | or/29-35 | 11437910 |
| 37 | 28 not 36 | 3807857 |
| 38 | 5 and 10 and 11 and 37 | 2504 |
| 39 | limit 38 to yr=2014 - current | 2484 |
| 40 | limit 39 to english | 2470 |

Table S4: Advanced/metastatic melanoma, post-approval era: MEDLINE search strategy

*Database: Embase <1974 to 2023 July 7>; Search date: July 10, 2023*

| **Line** | **Search terms** | **Hits** |
| --- | --- | --- |
| 1 | exp skin tumor/ or exp skin neoplasms/ or exp melanoma/ | 208384 |
| 2 | (((skin) adj3 (neoplasm$ or cancer$ or tumo?r* or carcinoma$ or adenocarcinoma$ or sarcoma$)) or melanoma).mp. | 259840 |
| 3 | or/1-2 | 265555 |
| 4 | exp metastasis/ or exp neoplasm metastasis/ or (advance$ or metasta$ or recurr$ or unresect$ or non-resect$ or disseminated or stage 3 or stage III* or stage 4 or stage IV* or spread$ or migration$ or progress$ or invasive or aggressive or "not operable" or untreatable or "not treatable" or secondary or incurable or "not curable").mp. | 5285610 |
| 5 | 3 and 4 | 111940 |
| 6 | exp atezolizumab/ or (atezolizumab or MPDL-3280A or MPDL3280A or RG7446 or RG-7446 or tecentriq).mp. | 3055 |
| 7 | exp nivolumab/ or (nivolumab or opdivo or ONO-4538 or BMS-936558 or MDX1106).mp. | 9609 |
| 8 | exp pembrolizumab/ or (pembrolizumab or MK-3475 or SCH-900475 or lambrolizumab or keytruda).mp. | 8916 |
| 9 | exp immune checkpoint inhibitors/ or (checkpoint or ICI* or (PD1 or PD-1 or PDL1 or PD-L1) and (inhibit*)).mp. | 68254 |
| 10 | or/6-9 | 73495 |
| 11 | exp overall survival/ or exp survival rate/ or exp life expectancy/ or exp survival analysis/ or (surviv* or OS or mortality or death* or die* or life expectancy).mp. | 4656980 |
| 12 | Epidemiologic studies/ | 9353 |
| 13 | Exp case control studies/ | 1428358 |
| 14 | Exp cohort studies/ | 2498788 |
| 15 | Case control.tw. | 154046 |
| 16 | (cohort adj (study or studies)).tw. | 316754 |
| 17 | Cohort analy$.tw. | 11810 |
| 18 | (Follow up adj (study or studies)).tw. | 56299 |
| 19 | (observational adj (study or studies)).tw. | 161394 |
| 20 | Longitudinal.tw. | 322757 |
| 21 | Retrospective.tw. | 745567 |
| 22 | Cross sectional.tw. | 513409 |
| 23 | Cross-sectional studies/ | 471331 |
| 24 | or/12-23 | 3796175 |
| 25 | case reports/ or case report.tw. | 2404681 |
| 26 | editorial/ | 656410 |
| 27 | historical article/ | 369352 |
| 28 | letter/ | 1222167 |
| 29 | review/ or systematic review/ or meta-analysis/ | 3343956 |
| 30 | or/25-29 | 7556164 |
| 31 | 24 not 30 | 3427154 |
| 32 | 5 and 10 and 11 and 31 | 1097 |
| 33 | limit 32 to yr=2014 - current | 1070 |
| 34 | limit 33 to english | 1066 |

## Study Selection

**Figure S1: PRISMA flow diagram: advanced/metastatic melanoma studies in the pre-approval era**


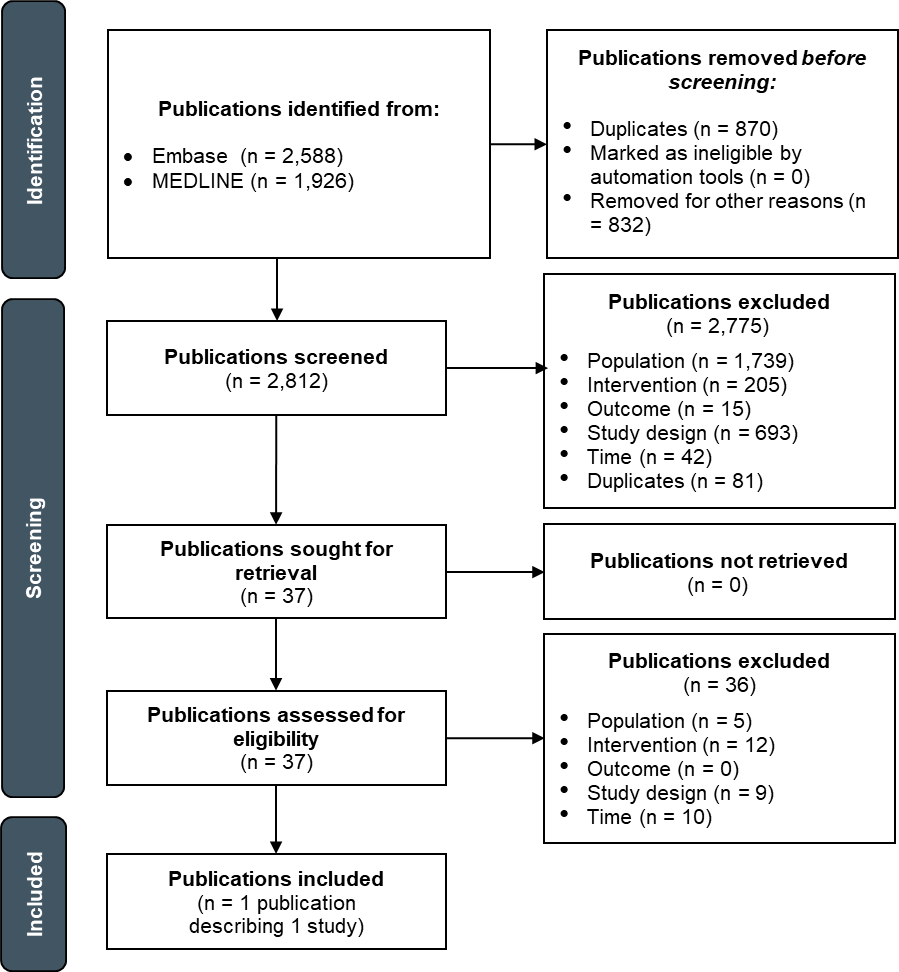


***Note:*** *“Removed for other reasons” refers to publications with irrelevant article types (e.g., conference abstracts, commentaries, review articles) according to metadata in the database export files.*

**Figure S2: PRISMA flow diagram: advanced/metastatic melanoma studies in the post-approval era**


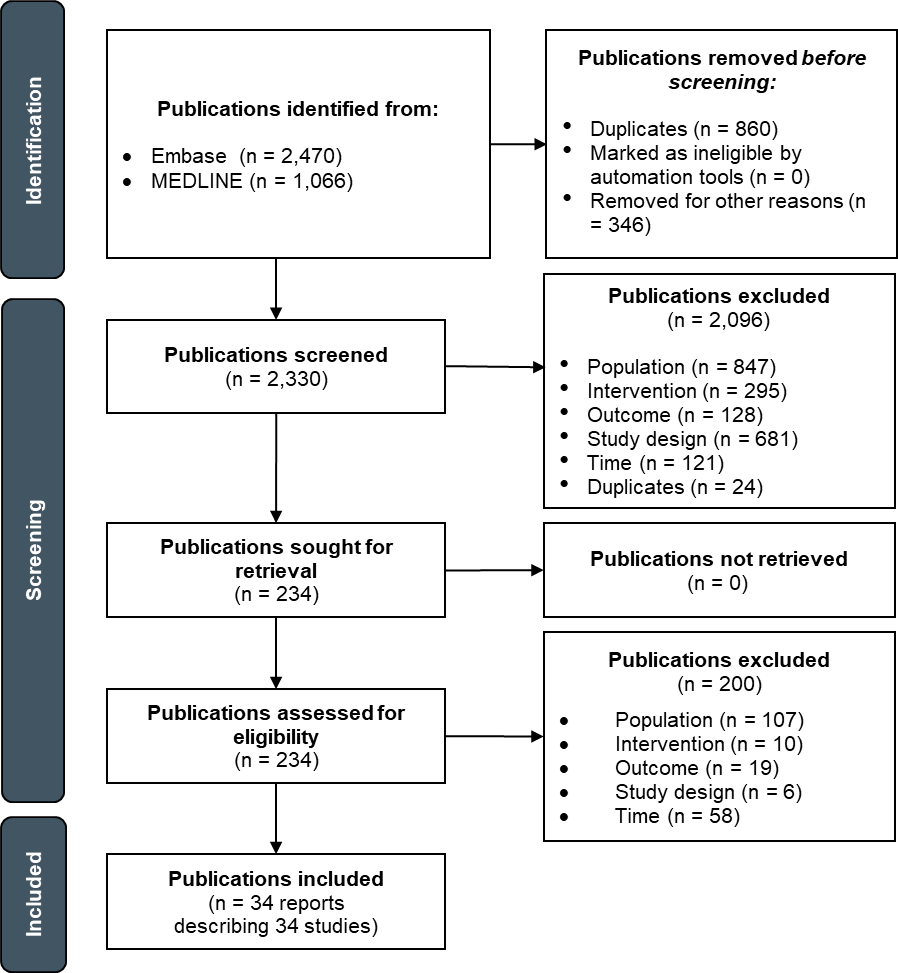


***Note:*** *“Removed for other reasons” refers to publications with irrelevant article types (e.g., conference abstracts, commentaries, review articles) according to metadata in the database export files.*

## Study Characteristics

Table S5: Characteristics of advanced/metastatic melanoma studies

| **Study ID** | **Study design** | **Country/**  **region** | **Treatment(s) evaluated** | **N** |
| --- | --- | --- | --- | --- |
|  |  |  |  |  |
| Pre-approval era | | | | |
| Fennira 2014 | Retrospective cohort | France | Vemurafenib | 50 |
| Post-approval era | | | | |
| Ab Rahman 2023 | Retrospective cohort | New Zealand | Pembrolizumab or nivolumab | 597 |
| Afrasanie 2023 | Retrospective cohort | Romania | Nivolumab | 51 |
| Afzal and Shirai 2018 | Retrospective cohort | US | Ipilimumab, pembrolizumab, or nivolumab; ipilimumab, pembrolizumab, or nivolumab + denosumab | 37 |
| Amaral 2020 | Retrospective cohort | Germany | Pembrolizumab or nivolumab, nivolumab + ipilimumab | 319 |
| Board 2021 | Retrospective cohort | England | Ipilimumab, pembrolizumab, ipilimumab + nivolumab, nivolumab | 2322 |
| Casarotto 2021 | Prospective cohort | France | Pembrolizumab | 223 |
| Cowey 2020 | Retrospective cohort | US | Pembrolizumab or nivolumab | 224 |
| Cowey 2018 | Retrospective cohort | US | Pembrolizumab | 168 |
| Cowey 2021 | Retrospective cohort | US | Pembrolizumab | 303 |
| Czarnecka 2019 | Ambispective cohort | Poland | Pembrolizumab or nivolumab | 253 |
| Hribernik 2020 | Retrospective cohort | Slovenia | Pembrolizumab | 138 |
| Hurkmans 2020 | Prospective cohort | Netherlands | Pembrolizumab, nivolumab | 107 |
| Inozume 2023 | Retrospective cohort | Japan | Anti-PD-1 agent ± anti-CTLA-4 agent | 146 |
| Iravani 2023 | Retrospective cohort | Australia | Nivolumab + ipilimumab | 122 |
| Kartolo 2022 | Retrospective cohort | Canada | Nivolumab or pembrolizumab, ipilimumab + nivolumab | 67 |
| Kuzmanovszki 2022 | Retrospective cohort | Hungary | Pembrolizumab, nivolumab | 119 |
| Liu 2019 | Retrospective cohort | US | Pembrolizumab | 532 |
| Mason 2020 | Retrospective cohort | Australia | Nivolumab + ipilimumab | 152 |
| Mohr 2022 | Retrospective cohort | Germany | Pembrolizumab | 664 |
| Monestier 2021 | Retrospective cohort | France | Nivolumab | 400 |
| O'Sullivan 2023 | Retrospective cohort | Canada | Anti-PD-1 agent | 434 |
| Parakh 2019 | Retrospective cohort | Australia | Nivolumab + ipilimumab + nivolumab maintenance | 45 |
| Pavlick 2021 | Retrospective cohort | US | Nivolumab + ipilimumab, BRAF + MEK inhibitors, other treatment | 557 |
| Peisen 2022 | Retrospective cohort | Germany | Nivolumab or pembrolizumab, nivolumab + ipilimumab | 262 |
| Rivas 2022 | Retrospective cohort | France | Nivolumab or pembrolizumab, nivolumab + ipilimumab | 29 |
| Stein 2020 | Retrospective cohort | France | Pembrolizumab or nivolumab | 239 |
| Suo 2020 | Retrospective cohort | Canada | Pembrolizumab, nivolumab | 186 |
| Tarhini 2019 | Retrospective cohort | US | Nivolumab, nivolumab + ipilimumab, pembrolizumab | 487 |
| van Breeschoten 2021 | Prospective cohort | Netherlands | Pembrolizumab or nivolumab | 584 |
| Van Zeijl 2020 | Prospective cohort | Netherlands | Pembrolizumab or nivolumab | 1394 |
| Van Zeijl 2023 | Prospective cohort | Netherlands | Nivolumab + ipilimumab ± nivolumab maintenance | 709 |
| Wei 2019 | Retrospective cohort | Scotland | Pembrolizumab monotherapy | 51 |
| Wilson 2021 | Retrospective cohort | UK | Nivolumab + ipilimumab + nivolumab maintenance | 58 |
| Zaremba 2023 | Prospective cohort | Germany | Pembrolizumab, nivolumab, nivolumab + ipilimumab | 637 |

**Abbreviations:** BRAF, B-Raf proto-oncogene; MEK, mitogen-activated protein kinase kinase; PD-(L)1, programmed death (ligand)-1; UK, United Kingdom; US, United States.

## Patient Characteristics

Table S6: Patient characteristics in advanced/metastatic melanoma studies

| **Study ID** | **Treatment** | **Overall/**  **subgroup** | **N** | **Age, years, median (range)** | **Male, %** | **ECOG PS**  **0-1, %** | **White race, %** | **BRAF status** | | | |
| --- | --- | --- | --- | --- | --- | --- | --- | --- | --- | --- | --- |
|  |  |  |  |  |  |  |  | **V600 mutation (any type), %** | **V600E mutation, %** | **V600K mutation, %** | **Unspecified mutation, %** |
| Pre-approval era | | | | | | | | | | | |
| Fennira 2014 | Vemurafenib | Overall | 50 | 58 (51-69)^a^ | 58 | 86 | -- | -- | 100 | -- | -- |
| Post-approval era | | | | | | | | | | | |
| Ab Rahman 2023 | Pembrolizumab or nivolumab | Overall | 597 | -- | 67 | -- | 92 | -- | -- | -- | -- |
| Afrasanie 2023 | Nivolumab | Overall | 51 | 61.5  (18-88) | 62.7 | 86.2 | -- | -- | 9.8 | -- | -- |
| Afzal and Shirai 2018 | Ipilimumab, pembrolizumab, or nivolumab ± denosumab | Overall | 37 | 66.67 (11.76)^b^ | 64.86 | 100 | -- | -- | -- | -- | -- |
|  | Ipilimumab, pembrolizumab, or nivolumab | Cohort A | 26 | 64.65 (10.93)^b^ | 61.53 | 100 | -- | -- | -- | -- | -- |
|  | Ipilimumab, pembrolizumab, or nivolumab + denosumab | Cohort B | 11 | 62.45 (10.68)^b^ | 72.72 | 100 | -- | -- | -- | -- | -- |
| Amaral 2020 | Pembrolizumab or nivolumab | Overall | 174 | 70 (59-79)^a^ | 62.6 | -- | -- | -- | -- | -- | -- |
|  | Nivolumab + ipilimumab | Overall | 145 | 67 (52-75)^a^ | 57.2 | -- | -- | -- | -- | -- | -- |
| Board 2021 | Ipilimumab, pembrolizumab, ipilimumab + nivolumab, or nivolumab | Overall | 2322 | 66 (17-97) | -- | -- | -- | -- | -- | -- | -- |
|  | Ipilimumab | Subgroup | 724 | -- | 65 | 68 | -- | -- | -- | -- | -- |
|  | Pembrolizumab | Subgroup | 1174 | -- | 63 | 78 | -- | -- | -- | -- | -- |
|  | Ipilimumab + Nivolumab | Subgroup | 372 | -- | 66 | 83 | -- | -- | -- | -- | -- |
|  | Nivolumab | Subgroup | 52 | -- | 71 | 75 | -- | -- | -- | -- | -- |
| Casarotto 2021 | Pembrolizumab | 1L | 134 | 72 (24-90) | 49 | 94 | -- | -- | -- | -- | 6 |
|  |  | BRAF WT | 130 | 73 (24-90) | 49 | -- | -- | -- | -- | -- | 0 |
|  |  | BRAF mutated | 93 | 56 (20-88) | 54 | -- | -- | -- | -- | -- | 100 |
| Cowey 2020 | Pembrolizumab or nivolumab | Overall | 81 | 62 (26-90+) | 61.7 | 75.3 | 85.2 | -- | -- | -- | 100 |
| Cowey 2018 | Pembrolizumab | Overall | 168 | 66 (26-90+) | 65 | 64 | 99 | -- | -- | -- | 35 |
| Cowey 2021 | Pembrolizumab | 1L | 119 | 70 (26-90+) | 65.5 | 74 | 92.4 | -- | -- | -- | 25.2 |
| Czarnecka 2019 | Pembrolizumab or nivolumab | Overall | 153 | -- | 55.56 | 98.7 | -- | -- | -- | -- | 22.22 |
| Hribernik 2020 | Pembrolizumab | Overall | 138 | 65.4 (25-87) | 60.9 | 89.8 | 100 | 18.1 | 15.9 | -- | -- |
| Hurkmans 2020 | Pembrolizumab or nivolumab | Overall | 107 | 66 (55-73)^a^ | 58 | 92 | -- | -- | -- | -- | -- |
| Inozume 2023 | Anti-PD-1 | Overall | 113 | 71 (26-95) | 54 | -- | -- | -- | -- | -- | 0 |
|  | Anti-PD-1 + anti-CTLA-4 | Overall | 33 | 62 (32-87) | 61 | -- | -- | -- | -- | -- | 0 |
| Iravani 2023 | Nivolumab + ipilimumab | Overall | 122 | 61 (18-79) | 73 | 97 | -- | -- | -- | -- | -- |
| Kartolo 2022 | Nivolumab or pembrolizumab monotherapy | Overall | 52 | -- | 67 | 77 | -- | 0 | -- | -- | -- |
| Kuzmanovszki 2022 | Pembrolizumab or nivolumab | Overall | 119 | 69 (57-75)^a^ | 57.1 | 98.28 | -- | 26.1 | 18.5 | 6.7 | -- |
| Liu 2019 | Pembrolizumab | Overall | 315 | 71 (18-84) | 67.9 | 79.6 | 92 | -- | -- | -- | 31.7 |
| Mason 2020 | Nivolumab + ipilimumab + nivolumab maintenance | Treatment-naïve, all patients | 60 | 60 (33-78) | 68 | 93 | -- | -- | -- | -- | -- |
|  |  | Treatment-naïve, BRAF mutated | 5 | 58 (33-69) | 100 | 100 | -- | 100 | -- | -- | -- |
| Mohr 2022 | Pembrolizumab | Overall | 664 | 70 (22-96) | 59.9 | 62.2 | -- | -- | -- | -- | 32.4 |
| Monestier 2021 | Nivolumab | Overall | 400 | 66 (--) | 61.3 | 85.7 | -- | -- | -- | -- | 34 |
| O'Sullivan 2023 | Anti-PD-1 therapies, targeted therapy, ipilimumab, or ipilimumab + nivolumab | Overall | 434 | -- | -- | -- | -- | -- | -- | -- | 5.1 |
| Parakh 2019 | Nivolumab + ipilimumab + nivolumab maintenance | Overall | 45 | 63 (20-82) | 67 | 87 | -- | 38 | -- | -- | -- |
| Pavlick 2021 | Nivolumab + ipilimumab | Overall | 107 | 58 (14)^b^ | 65 | 79 | 87 | -- | -- | -- | 100 |
| Peisen 2022 | Nivolumab, pembrolizumab, or nivolumab + ipilimumab | Overall | 262 | 70 (--) | 58^h^ | -- | -- | 28 | -- | -- | -- |
| Rivas 2022 | Nivolumab, pembrolizumab, or nivolumab + ipilimumab | Overall | 29 | 64 (56-79)^a^ | 52 | 93.1 | -- | -- | -- | -- | 93.1 |
| Stein 2020 | Pembrolizumab or nivolumab | Overall | 239 | 68 (58.5-77)^a^ | 55 | -- | -- | -- | -- | -- | -- |
| Suo 2020 | Pembrolizumab or nivolumab | Overall | 186 | 63.5 (55-74) | 58.6 | 83.3 | -- | 27.4 | -- | -- | -- |
| Tarhini 2019 | Nivolumab | Overall | 69 | 68.3 (12.2)^b^ | 53.6 | 88.4 | 97.1 | -- | -- | -- | 10.1 |
|  | Nivolumab + ipilimumab | Overall | 60 | 57.1 (11.3)^b^ | 66.7 | 93.4 | 96.7 | -- | -- | -- | 16.9 |
|  | Pembrolizumab | Overall | 95 | 62.6 (13.5)^b^ | 53.6 | 86.3 | 91.6 | -- | -- | -- | 20 |
| van Breeschoten 2021 | Pembrolizumab or nivolumab | Original sample | 254 | 62 (22-87) | 57.1 | 91.3 | -- | -- | 83.5 | 13.8 | -- |
| Van Zeijl 2020 | Pembrolizumab or nivolumab | Overall | 550 | 65 (21-94) | 61.5 | 95.4 | -- | -- | -- | -- | 39.5 |
|  | Pembrolizumab or nivolumab and others | BRAF WT | 323 | 68 (21-94) | 63.5 | 94.4 | -- | -- | -- | -- | 0 |
|  | Pembrolizumab or nivolumab and others | BRAF mutated | 217 | 62 (22-85) | 59 | 97.1 | -- | -- | -- | -- | 100 |
| Van Zeijl 2023 | Nivolumab + ipilimumab ± nivolumab maintenance | Overall | 709 | 61 (21-85) | 62.1 | 92.4 | -- | -- | -- | -- | 41.5 |
| Wei 2019 | Pembrolizumab | Overall | 33 | 64.33 (--) | 75.76 | -- | -- | -- | -- | -- | -- |
| Wilson 2021 | Anti-PD-1 and anti-CTLA-4 immunotherapy | Overall | 58 | 61.5  (33-80) | 62.1 | -- | -- | 37.9 | -- | -- | -- |
| Zaremba 2023 | Pembrolizumab, nivolumab, or nivolumab + ipilimumab | Overall | 637 | 67.7  (13.8-92.6) | 64.5 | 58.1 | -- | -- | -- | -- | 0 |
|  |  | PD-L1-positive | 37 | -- | 62.2 | 81.1 | -- | -- | -- | -- | 0 |
|  |  | PD-L1-negative | 45 | -- | 64.4 | 75.6 | -- | -- | -- | -- | 0 |

**Notes: a)** Median (interquartile range); **b)** Median (standard deviation); **Abbreviations:** BRAF, B-RAF proto-oncogene; CTLA4, cytotoxic T-lymphocyte-associated protein 4; ECOG, Eastern Cooperative Oncology Group; L, line; PD-(L)1, programmed death (ligand)-1; PS, performance score; WT, wild-type.

## Outcomes

**Table S7: Summary of mOS in advanced/metastatic melanoma studies**

|  | **Pre-approval era** | | | **Post-approval era** | | |
| --- | --- | --- | --- | --- | --- | --- |
|  | **No. treatment groups** | **mOS in months, range** | **Median follow up in months, range** | **No. treatment groups** | **mOS in months, range** | **Median follow up in months, range** |
| **BRAF mutation status** | | | | | | |
| Any mutation^a^ | 1 | 14.2 | 19.9 | 9 | 15.9-NR | 11.3-47.18 |
| No mutation^b^ | 0 | -- | -- | 13 | 15.7-NR | 12.1-36 |
| All-comer^c^ | 0 | -- | -- | 8 | 17.4-57 | 8.7-42 |
| Unspecified^d^ | 0 | -- | -- | 4 | 14.2-NR | 7-25 |

***Notes: a)*** *Treatment groups including ≥80% patients with any type of BRAF mutation.* ***b)*** *Treatment groups including <20% of patients with any type of BRAF mutation.* ***c)*** *Treatment groups including ≥20% but <80% patients with any type of BRAF mutation.* ***d)*** *Treatment groups in studies in which BRAF mutation status was neither an eligibility criterion nor reported as a patient characteristic.* ***Abbreviations:*** *BRAF, B-Raf proto-oncogene; mOS, median overall survival; no., number; NR, not reached.*

Table S8: Overall survival by treatment group in advanced/metastatic melanoma studies

| **Study ID** | **Treatment** | **Overall/**  **subgroup** | **Follow-up duration, months (range)** | **Median OS, months (95% CI)** | **Landmark OS** | | | | | |
| --- | --- | --- | --- | --- | --- | --- | --- | --- | --- | --- |
|  |  |  |  |  | **3-mo** | **6-mo** | **12-mo** | **18-mo** | **2-y** | **3-y** |
| **Pre-approval era** | | | | | | | | | | |
| Fennira 2014 | Vemurafenib | 1L | 19.9 (9.4-35.5) | 14.2 (9.1-NR) | -- | -- | -- | -- | -- | -- |
| **Post-approval era** | | | | | | | | | | |
| Ab Rahman 2023 | Pembrolizumab or nivolumab | Overall | 25 (18-32)^a^ | NR (--) | -- | -- | 72 | -- | 60 | -- |
| Afrasanie 2023 | Nivolumab | Overall | 36 (--)^b^ | 31 (20.1-41.8) | -- | -- | 70 | -- | 62.5 | -- |
| Afzal and Shirai 2018 | Ipilimumab, pembrolizumab, or nivolumab ± denosumab | Overall | -- | 22.8 (--) | -- | -- | -- | -- | -- | -- |
|  | Ipilimumab, pembrolizumab, or nivolumab | Cohort A | -- | 22.8 (16-36) | -- | 96.15 | 84.44 | 71.31 | 47.54 | 28.52 |
|  | Ipilimumab, pembrolizumab, or nivolumab + denosumab | Cohort B | -- | 57 (7.6-NA) | -- | 90.91 | 81.82 | 63.64 | 53.03 | 53.03 |
| Amaral 2020 | Pembrolizumab or nivolumab | Overall | 22 (--) | 26 (19.7-32.3) | -- | -- | 71.1 | -- | 53.3 | 41.3 |
|  | Nivolumab + ipilimumab | Overall | 22 (--) | 31 (17.2-44.8) | -- | -- | 72.8 | -- | 54.5 | 42.5 |
| Board 2021 | Ipilimumab | Overall | -- | -- | -- | -- | -- | -- | -- | 32 |
|  | Pembrolizumab | Overall | -- | -- | -- | -- | -- | -- | -- | 40 |
|  | Ipilimumab + nivolumab | Overall | -- | -- | -- | -- | -- | -- | -- | 56 |
|  | Nivolumab | Overall | -- | -- | -- | -- | -- | -- | -- | 51 |
| Casarotto 2021 | Pembrolizumab | 1L | 25.3 (2.3-30.9) | 32.6 (20.3-NR) | -- | 84.2 | 70.3 | 61.5 | 54.2 | 47 |
|  |  | 1L, BRAF WT | -- | NR (18.8-NR) | -- | -- | -- | -- | 51.8 | -- |
|  |  | 1L, BRAF mutated | -- | 32.6 (25.2-NR) | -- | -- | -- | -- | 69.3 | -- |
| Cowey 2020 | Pembrolizumab or nivolumab | Overall | 11.3 (0.4-41.9) | NR (20.3-NR) | -- | -- | 73.8 | -- | 57.7 | -- |
| Cowey 2018 | Pembrolizumab | 1L | -- | -- | -- | -- | 68 | -- | -- | -- |
| Cowey 2021 | Pembrolizumab | 1L | 18.2 (0.1-63.1) | 42.8 (24.8-NR) | -- | -- | -- | -- | -- | -- |
| Czarnecka 2019 | Pembrolizumab | Overall | 23.2 (0.5-38.8) | 18.1 (11.1-NR) | -- | -- | -- | -- | -- | -- |
|  | Nivolumab | Overall | 23.2 (0.5-38.8) | 26.4 (14.7-NR) | -- | -- | -- | -- | -- | -- |
|  | Pembrolizumab or nivolumab | BRAF mutated | 23.2 (0.5-38.8) | NR (14.7-NR) | -- | -- | 70 | -- | -- | -- |
|  |  | BRAF WT | 23.2 (0.5-38.8) | 18.1 (15-NR) | -- | -- | 59 | -- | -- | -- |
| Hribernik 2020 | Pembrolizumab | Overall | -- | 25.1 (14.7-35.6) | -- | -- | -- | -- | -- | -- |
|  |  | Cutaneous | -- | 32.7 (NR-NR) | -- | -- | -- | -- | -- | -- |
| Hurkmans 2020 | Pembrolizumab or nivolumab | Overall | 1.8 (10)^c, d^ | 36.2 (--) | -- | -- | -- | -- | -- | -- |
| Inozume 2023 | Anti-PD-1 | Overall | -- | 28.1 (14.5-32.2) | -- | -- | -- | -- | -- | -- |
|  | Anti-PD-1 + anti-CTL-A4 | Overall | -- | NR (8.9-NR) | -- | -- | -- | -- | -- | -- |
| Iravani 2023 | Nivolumab + ipilimumab | 1L | 42 (--) | -- | -- | -- | 71 | -- | 69 | -- |
| Kartolo 2022 | Nivolumab or pembrolizumab monotherapy | Overall | 15.1 (--) | 15.7 (--) | -- | -- | -- | -- | -- | -- |
| Kuzmanovszki 2022 | Pembrolizumab or nivolumab | 1L | -- | 94.14 (33.01-155.27)^e^ | -- | -- | -- | -- | -- | -- |
|  |  | BRAF WT | -- | 103.14 (41.07-165.21)^e^ | -- | -- | -- | -- | -- | -- |
| Liu 2019 | Pembrolizumab | 1L | 13.6 (0.03-38.6) | NR (21.2-NR) | -- | -- | 65.6 | -- | 52.8 | -- |
|  |  | 1L, BRAF WT | -- | NR (32.3-NR) | -- | -- | 65.61 | -- | 54.96 | -- |
|  |  | 1L, BRAF mutated | -- | NR (16.8-NR) | -- | -- | 73.57 | -- | 51.88 | -- |
| Mason 2020 | Nivolumab + ipilimumab + nivolumab maintenance | Treatment-naïve, all patients | 6.1 (5.8-6.7)^f^ | 14.2 (8-NR) | -- | -- | 65 | -- | -- | -- |
|  |  | Treatment-naïve, BRAF mutant | 6.1 (5.8-6.7)^f^ | 14.2 (NR-NR) | -- | -- | -- | -- | -- | -- |
| Mohr 2022 | Pembrolizumab | 1L | 32.8 (29.4-38.3)^f^ | 30.9 (23.8-41.6) | -- | 85.2 | 72.9 | 62.8 | 55.3 | -- |
| Monestier 2021 | Nivolumab | 1L | -- | 22.5 (11.5-NR) | -- | -- | 59.7 | -- | 47.5 | -- |
| O'Sullivan 2023 | Anti-PD-1 | 1L | -- | -- | -- | -- | -- | -- | 54 | -- |
| Parakh 2019 | Nivolumab + ipilimumab + nivolumab maintenance | Treatment-naïve | 8.7 (0.33-25.9) | 17.4 (--) | -- | -- | -- | -- | -- | -- |
| Pavlick 2021 | Nivolumab + ipilimumab | Overall | 11.3 (0.1-48.4) | 48.4 (21.4-48.4) | -- | -- | 69 | -- | 60 | 58 |
| Peisen 2022 | Nivolumab, pembrolizumab, or nivolumab + ipilimumab | Overall | -- | 22.1 (16.6-29.6) | -- | 69 | 44 | -- | -- | -- |
| Rivas 2022 | Nivolumab, pembrolizumab, or nivolumab + ipilimumab | Overall | 47.18 (--) | 51.2 (13.6-NR) | -- | -- | -- | -- | 58.6 | -- |
| Stein 2020 | Pembrolizumab or nivolumab | 1L | 352 (--)^g^ | -- | -- | -- | -- | -- | -- | -- |
| Suo 2020 | Pembrolizumab or nivolumab | 1L | 24 (--) | -- | -- | -- | 27 | -- | -- | -- |
| Tarhini 2019 | Nivolumab | Overall | 12.1 (10.6-14.5)^a^ | NR (--) | -- | 87 | 77.9 | 77.9 | -- | -- |
|  | Nivolumab + ipilimumab | Overall | 14.5 (5.2- --)^c^ | NR (--) | -- | 93.3 | 86.6 | 81.8 | -- | -- |
|  | Pembrolizumab | Overall | 16.5 (7.5- --)^c^ | NR (--) | -- | 92.6 | 81.4 | 74 | -- | -- |
| van Breeschoten 2021 | Pembrolizumab or nivolumab | Original sample | 28.3 (--) | 42.3 (34.8-NR) | 91.3 | -- | 81.7 | -- | -- | -- |
| Van Zeijl 2020 | Pembrolizumab or nivolumab | Overall | 32 (--) | 23.6 (20.5-30) | -- | -- | 67 | -- | 49 | -- |
|  |  | BRAF WT | 32 (--) | 18.2 (13.9-22.8) | -- | -- | -- | -- | -- | -- |
|  |  | BRAF mutated | 32 (--) | 42.2 (27.5-NR) | -- | -- | -- | -- | -- | -- |
| Van Zeijl 2023 | Nivolumab + ipilimumab ± nivolumab maintenance | Overall | 26.3 (24.8-28.8)^f^ | 28.7 (20.7-42.2) | -- | -- | -- | -- | 51 | -- |
|  |  | BRAF WT | -- | -- | -- | -- | -- | -- | -- | -- |
|  |  | BRAF mutated | -- | -- | -- | -- | -- | -- | -- | -- |
| Wei 2019 | Pembrolizumab | Overall | 7 (1-55) | 21.6 (16.4-26.7) | -- | -- | -- | -- | -- | -- |
| Wilson 2021 | Anti-PD-1 | 1L | -- | 30.9 (--) | -- | -- | -- | -- | -- | -- |
|  | Nivolumab + ipilimumab + nivolumab maintenance | 1L | -- | 22.6 (--) | -- | -- | -- | -- | -- | -- |
| Zaremba 2023 | Pembrolizumab, nivolumab, or nivolumab + ipilimumab | Overall | 35.9 (18-54.6)^a^ | 33.4 (27.7-40.1) | -- | -- | 74 | -- | 55.6 | -- |
|  |  | PD-L1-positive | -- | -- | -- | -- | -- | -- | -- | -- |
|  |  | PD-L1-negative | -- | -- | -- | -- | -- | -- | -- | -- |

**Notes: a)** Median (interquartile range); **b)** Discrepancy between median follow-up reported in abstract versus text; **c)** Mean (standard deviation); **d)** Reported in years; **e)** Reported in weeks; **f)** Median (95% CI); **g)** Reported in days**. Abbreviations:** BRAF, B-RAF proto-oncogene; CI, confidence interval; CTLA4, cytotoxic T lymphocyte-associated protein-4; NR, not reached; OS, overall survival; y, year; L, line; PD-(L)1, programmed death (ligand)-1; WT, wild-type.

## Quality Assessment

Table S9: Quality assessment of advanced/metastatic melanoma studies

| **Study ID** | **Selection** | | | | **Comparability** | **Outcome** | | | **Total score** |
| --- | --- | --- | --- | --- | --- | --- | --- | --- | --- |
|  | **1** | **2** | **3** | **4** | **1** | **1** | **2** | **3** |  |
| **Pre-approval era** | | | | | | | | | |
| Fennira 2014 | 0 | NA | 1 | 1 | NA | 1 | 1 | 1 | 5 |
| **Post-approval era** | | | | | | | | | |
| Ab Rahman 2023 | 1 | NA | 1 | 1 | NA | 1 | 1 | 1 | 6 |
| Afrasanie 2023 | 0 | NA | 1 | 1 | NA | 1 | 1 | 1 | 5 |
| Afzal and Shirai 2018 | 0 | NA | 1 | 1 | NA | 1 | 0 | 1 | 4 |
| Amaral 2020 | 0 | NA | 1 | 1 | NA | 1 | 1 | 1 | 5 |
| Board 2021 | 1 | NA | 1 | 1 | NA | 1 | 0 | 1 | 5 |
| Casarotto 2021 | 1 | NA | 1 | 1 | NA | 1 | 1 | 1 | 6 |
| Cowey 2020 | 1 | NA | 1 | 1 | NA | 1 | 1 | 1 | 6 |
| Cowey 2018 | 1 | NA | 1 | 1 | NA | 1 | 1 | 1 | 6 |
| Cowey 2021 | 1 | NA | 1 | 1 | NA | 1 | 1 | 1 | 6 |
| Czarnecka 2019 | 0 | NA | 1 | 1 | NA | 1 | 1 | 1 | 5 |
| Hribernik 2020 | 0 | NA | 1 | 1 | NA | 1 | 0 | 1 | 4 |
| Hurkmans 2020 | 0 | NA | 1 | 1 | NA | 1 | 1 | 1 | 5 |
| Inozume 2023 | 1 | NA | 1 | 1 | NA | 1 | 0 | 1 | 5 |
| Iravani 2023 | 0 | NA | 1 | 1 | NA | 1 | 1 | 1 | 5 |
| Kartolo 2022 | 0 | NA | 1 | 1 | NA | 1 | 1 | 1 | 5 |
| Kuzmanovszki 2022 | 0 | NA | 1 | 1 | NA | 1 | 1 | 1 | 5 |
| Liu 2019 | 1 | NA | 1 | 1 | NA | 1 | 1 | 1 | 6 |
| Mason 2020 | 1 | NA | 1 | 1 | NA | 1 | 1 | 1 | 6 |
| Mohr 2022 | 1 | NA | 1 | 1 | NA | 1 | 1 | 1 | 6 |
| Monestier 2021 | 1 | NA | 1 | 1 | NA | 1 | 0 | 1 | 5 |
| O'Sullivan 2023 | 1 | NA | 1 | 1 | NA | 1 | 0 | 1 | 5 |
| Parakh 2019 | 1 | NA | 1 | 1 | NA | 1 | 1 | 1 | 6 |
| Pavlick 2021 | 1 | NA | 1 | 1 | NA | 1 | 1 | 1 | 6 |
| Peisen 2022 | 0 | NA | 1 | 1 | NA | 1 | 0 | 1 | 4 |
| Rivas 2022 | 0 | NA | 1 | 1 | NA | 1 | 1 | 1 | 5 |
| Stein 2020 | 0 | NA | 1 | 1 | NA | 1 | 1 | 1 | 5 |
| Suo 2020 | 1 | NA | 1 | 1 | NA | 1 | 1 | 1 | 6 |
| Tarhini 2019 | 1 | NA | 1 | 1 | NA | 1 | 1 | 1 | 6 |
| van Breeschoten 2021 | 1 | NA | 1 | 1 | NA | 1 | 1 | 1 | 6 |
| Van Zeijl 2020 | 1 | NA | 1 | 1 | NA | 1 | 1 | 1 | 6 |
| Van Zeijl 2023 | 1 | NA | 1 | 1 | NA | 1 | 1 | 1 | 6 |
| Wei 2019 | 0 | NA | 1 | 1 | NA | 1 | 1 | 1 | 5 |
| Wilson 2021 | 0 | NA | 1 | 1 | NA | 1 | 0 | 1 | 4 |
| Zaremba 2023 | 1 | NA | 1 | 1 | NA | 1 | 1 | 1 | 6 |

*Abbreviations: NA, not applicable.*

# Advanced/Metastatic NSCLC

## Literature Search Strategies

Table S10: Advanced/metastatic NSCLC, pre-approval era: Embase search strategy

*Database: Embase <1974 to 2023 July 7>; Search date: July 10, 2023*

| **Line** | **Search terms** | **Hits** |
| --- | --- | --- |
| 1 | exp carcinoma, non-small-cell-lung/ | 155927 |
| 2 | (((lung cancer* or lung carcinoma* or lung neoplasm* or lung tumo?r* or lung tumour* or pulmonary cancer* or pulmonary carcinoma*) adj5 (non small cell or nonsmall cell)) or nsclc or lung adenocarcinoma* or pulmonary adenocarcinoma*).mp. | 218820 |
| 3 | or/1-2 | 223625 |
| 4 | exp metastasis/ or exp neoplasm metastasis/ or (advance$ or metasta$ or recurr$ or unresect$ or non-resect$ or disseminated or stage 3 or stage III* or stage 4 or stage IV* or spread$ or migration$ or progress$ or invasive or aggressive or "not operable" or untreatable or "not treatable" or secondary or incurable or "not curable").mp. | 7409869 |
| 5 | 3 and 4 | 152086 |
| 6 | exp overall survival/ or exp survival rate/ or exp life expectancy/ or exp survival analysis/ or (surviv* or OS or mortality or death* or die* or life expectancy).mp. | 6478513 |
| 7 | Clinical study/ | 163725 |
| 8 | Case control study/ | 207756 |
| 9 | Family study/ | 25786 |
| 10 | Longitudinal study/ | 196031 |
| 11 | Retrospective study/ | 1490609 |
| 12 | Prospective study/ | 886615 |
| 13 | Randomized controlled trials/ | 264163 |
| 14 | 12 not 13 | 875476 |
| 15 | Prospective study/ | 886615 |
| 16 | Cohort analysis/ | 1056494 |
| 17 | (Cohort adj (study or studies)).mp. | 485180 |
| 18 | (Case control adj (study or studies)).tw. | 170347 |
| 19 | (follow up adj (study or studies)).tw. | 74216 |
| 20 | (observational adj (study or studies)).tw. | 257702 |
| 21 | (epidemiologic$ adj (study or studies)).tw. | 123233 |
| 22 | (cross sectional adj (study or studies)).tw. | 343578 |
| 23 | or/7-11,14-22 | 3974056 |
| 24 | case study/ | 99618 |
| 25 | case report/ or case report.tw. | 2963471 |
| 26 | conference abstract/ or conference paper/ or conference review/ | 2711315 |
| 27 | editorial/ | 751065 |
| 28 | letter/ | 1234492 |
| 29 | note/ | 886897 |
| 30 | review/ or short survey/ | 3514301 |
| 31 | or/24-30 | 11437910 |
| 32 | 23 not 31 | 3058593 |
| 33 | 5 and 6 and 32 | 18159 |
| 34 | limit 33 to yr=2010-2017 | 6907 |
| 35 | limit 34 to english | 6659 |

Table S11: Advanced/metastatic NSCLC, pre-approval era: MEDLINE search strategy

*Database: Ovid MEDLINE(R) and Epub Ahead of Print, In-Process, In-Data-Review & Other Non-Indexed Citations and Daily 1946 to July 06, 2023; Search date: July 10, 2023*

| **Line** | **Search terms** | **Hits** |
| --- | --- | --- |
| 1 | exp carcinoma, non-small-cell-lung/ | 70465 |
| 2 | (((lung cancer* or lung carcinoma* or lung neoplasm* or lung tumo?r* or lung tumour* or pulmonary cancer* or pulmonary carcinoma*) adj5 (non small cell or nonsmall cell)) or nsclc or lung adenocarcinoma* or pulmonary adenocarcinoma*).mp. | 111937 |
| 3 | or/1-2 | 122062 |
| 4 | exp metastasis/ or exp neoplasm metastasis/ or (advance$ or metasta$ or recurr$ or unresect$ or non-resect$ or disseminated or stage 3 or stage III* or stage 4 or stage IV* or spread$ or migration$ or progress$ or invasive or aggressive or "not operable" or untreatable or "not treatable" or secondary or incurable or "not curable").mp. | 5285610 |
| 5 | 3 and 4 | 75719 |
| 6 | exp overall survival/ or exp survival rate/ or exp life expectancy/ or exp survival analysis/ or (surviv* or OS or mortality or death* or die* or life expectancy).mp. | 4656980 |
| 7 | Epidemiologic studies/ | 9353 |
| 8 | exp case control studies/ | 1428358 |
| 9 | exp cohort studies/ | 2498788 |
| 10 | Case control.tw. | 154046 |
| 11 | (cohort adj (study or studies)).tw. | 316754 |
| 12 | Cohort analy$.tw. | 11810 |
| 13 | (Follow up adj (study or studies)).tw. | 56299 |
| 14 | (observational adj (study or studies)).tw. | 161394 |
| 15 | Longitudinal.tw. | 322757 |
| 16 | Retrospective.tw. | 745567 |
| 17 | Cross sectional.tw. | 513409 |
| 18 | Cross-sectional studies/ | 471331 |
| 19 | or/7-18 | 3796175 |
| 20 | case reports/ or case report.tw. | 2404681 |
| 21 | editorial/ | 656410 |
| 22 | historical article/ | 369352 |
| 23 | letter/ | 1222167 |
| 24 | review/ or systematic review/ or meta-analysis/ | 3343956 |
| 25 | or/20-24 | 7556164 |
| 26 | 19 not 25 | 3427154 |
| 27 | 5 and 6 and 26 | 13020 |
| 28 | limit 27 to yr=2010-2017 | 4398 |
| 29 | limit 28 to english | 4138 |

Table S12: Advanced/metastatic NSCLC, post-approval era: Embase search strategy

*Database: Embase <1974 to 2023 July 7>; Search date: July 10, 2023*

| **Line** | **Search terms** | **Hits** |
| --- | --- | --- |
| 1 | exp carcinoma, non-small-cell-lung/ | 155927 |
| 2 | (((lung cancer* or lung carcinoma* or lung neoplasm* or lung tumo?r* or lung tumour* or pulmonary cancer* or pulmonary carcinoma*) adj5 (non small cell or nonsmall cell)) or nsclc or lung adenocarcinoma* or pulmonary adenocarcinoma*).mp. | 218820 |
| 3 | or/1-2 | 223625 |
| 4 | exp metastasis/ or exp neoplasm metastasis/ or (advance$ or metasta$ or recurr$ or unresect$ or non-resect$ or disseminated or stage 3 or stage III* or stage 4 or stage IV* or spread$ or migration$ or progress$ or invasive or aggressive or "not operable" or untreatable or "not treatable" or secondary or incurable or "not curable").mp. | 7409869 |
| 5 | 3 and 4 | 152086 |
| 6 | exp atezolizumab/ or (atezolizumab or MPDL-3280A or MPDL3280A or RG7446 or RG-7446 or tecentriq).mp. | 15638 |
| 7 | exp cemiplimab/ or (cemiplimab or REGN2810 or REGN-2810).mp. | 1820 |
| 8 | exp durvalumab/ or (durvalumab or MEDI-4736 or MEDI4736 or imfinzi).mp. | 10333 |
| 9 | exp nivolumab/ or (nivolumab or opdivo or ONO-4538 or BMS-936558 or MDX1106).mp. | 38765 |
| 10 | exp pembrolizumab/ or (pembrolizumab or MK-3475 or SCH-900475 or lambrolizumab or keytruda).mp. | 37947 |
| 11 | exp immune checkpoint inhibitors/ or ((checkpoint or ICI* or (PD1 or PD-1 or PDL1 or PD-L1)) and inhibit*).mp. | 141080 |
| 12 | or/6-11 | 176003 |
| 13 | exp overall survival/ or exp survival rate/ or exp life expectancy/ or exp survival analysis/ or (surviv* or OS or mortality or death* or die* or life expectancy).mp. | 6478513 |
| 14 | Clinical study/ | 163725 |
| 15 | Case control study/ | 207756 |
| 16 | Family study/ | 25786 |
| 17 | Longitudinal study/ | 196031 |
| 18 | Retrospective study/ | 1490609 |
| 19 | Prospective study/ | 886615 |
| 20 | Randomized controlled trials/ | 264163 |
| 21 | 19 not 20 | 875476 |
| 22 | Prospective study/ | 886615 |
| 23 | Cohort analysis/ | 1056494 |
| 24 | (Cohort adj (study or studies)).mp. | 485180 |
| 25 | (Case control adj (study or studies)).tw. | 170347 |
| 26 | (follow up adj (study or studies)).tw. | 74216 |
| 27 | (observational adj (study or studies)).tw. | 257702 |
| 28 | (epidemiologic$ adj (study or studies)).tw. | 123233 |
| 29 | (cross sectional adj (study or studies)).tw. | 343578 |
| 30 | or/14-18,21-29 | 3974056 |
| 31 | case study/ | 99618 |
| 32 | case report/ or case report.tw. | 2963471 |
| 33 | conference abstract/ or conference paper/ or conference review/ | 2711315 |
| 34 | editorial/ | 751065 |
| 35 | letter/ | 1234492 |
| 36 | note/ | 886897 |
| 37 | review/ or short survey/ | 3514301 |
| 38 | or/31-37 | 11437910 |
| 39 | 30 not 38 | 3058593 |
| 40 | 5 and 12 and 13 and 39 | 3236 |
| 41 | limit 40 to yr=2015 - current | 3214 |
| 42 | limit 41 to english | 3174 |

Table S13: Advanced/metastatic NSCLC, post-approval era: MEDLINE search strategy

*Database: Embase <1974 to 2023 July 7>; Search date: July 10, 2023*

| **Line** | **Search terms** | **Hits** |
| --- | --- | --- |
| 1 | exp carcinoma, non-small-cell-lung/ | 70465 |
| 2 | (((lung cancer* or lung carcinoma* or lung neoplasm* or lung tumo?r* or lung tumour* or pulmonary cancer* or pulmonary carcinoma*) adj5 (non small cell or nonsmall cell)) or nsclc or lung adenocarcinoma* or pulmonary adenocarcinoma*).mp. | 111937 |
| 3 | or/1-2 | 122062 |
| 4 | exp metastasis/ or exp neoplasm metastasis/ or (advance$ or metasta$ or recurr$ or unresect$ or non-resect$ or disseminated or stage 3 or stage III* or stage 4 or stage IV* or spread$ or migration$ or progress$ or invasive or aggressive or "not operable" or untreatable or "not treatable" or secondary or incurable or "not curable").mp. | 5285610 |
| 5 | 3 and 4 | 75719 |
| 6 | exp atezolizumab/ or (atezolizumab or MPDL-3280A or MPDL3280A or RG7446 or RG-7446 or tecentriq).mp. | 3055 |
| 7 | exp cemiplimab/ or (cemiplimab or REGN2810 or REGN-2810).mp. | 356 |
| 8 | exp durvalumab/ or (durvalumab or MEDI-4736 or MEDI4736 or imfinzi).mp. | 1496 |
| 9 | exp nivolumab/ or (nivolumab or opdivo or ONO-4538 or BMS-936558 or MDX1106).mp. | 9609 |
| 10 | exp pembrolizumab/ or (pembrolizumab or MK-3475 or SCH-900475 or lambrolizumab or keytruda).mp. | 8916 |
| 11 | exp immune checkpoint inhibitors/ or ((checkpoint or ICI* or (PD1 or PD-1 or PDL1 or PD-L1)) and inhibit*).mp. | 68254 |
| 12 | or/6-11 | 74088 |
| 13 | exp overall survival/ or exp survival rate/ or exp life expectancy/ or exp survival analysis/ or (surviv* or OS or mortality or death* or die* or life expectancy).mp. | 4656980 |
| 14 | Epidemiologic studies/ | 9353 |
| 15 | exp case control studies/ | 1428358 |
| 16 | exp cohort studies/ | 2498788 |
| 17 | Case control.tw. | 154046 |
| 18 | (cohort adj (study or studies)).tw. | 316754 |
| 19 | Cohort analy$.tw. | 11810 |
| 20 | (Follow up adj (study or studies)).tw. | 56299 |
| 21 | (observational adj (study or studies)).tw. | 161394 |
| 22 | Longitudinal.tw. | 322757 |
| 23 | Retrospective.tw. | 745567 |
| 24 | Cross sectional.tw. | 513409 |
| 25 | Cross-sectional studies/ | 471331 |
| 26 | or/14-25 | 3796175 |
| 27 | case reports/ or case report.tw. | 2404681 |
| 28 | editorial/ | 656410 |
| 29 | historical article/ | 369352 |
| 30 | letter/ | 1222167 |
| 31 | review/ or systematic review/ or meta-analysis/ | 3343956 |
| 32 | or/27-31 | 7556164 |
| 33 | 26 not 32 | 3427154 |
| 34 | 5 and 12 and 13 and 33 | 1546 |
| 35 | limit 34 to yr=2015 - current | 1545 |
| 36 | limit 35 to english | 1527 |

## Study Selection

**Figure S3: PRISMA flow diagram: advanced/metastatic NSCLC studies in the pre-approval era**


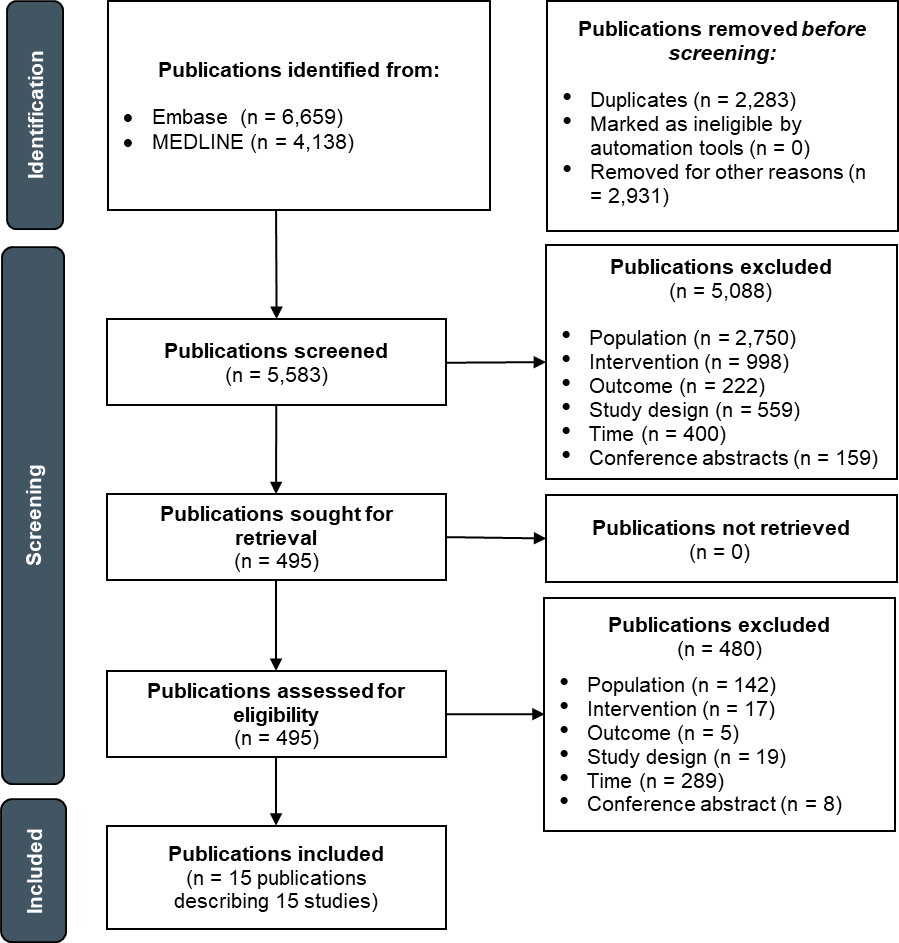


***Note:*** *“Removed for other reasons” refers to publications with irrelevant article types (e.g., conference abstracts, commentaries, review articles) according to metadata in the database export files.*

**Figure S4: PRISMA flow diagram: advanced/metastatic NSCLC studies in the post-approval era**


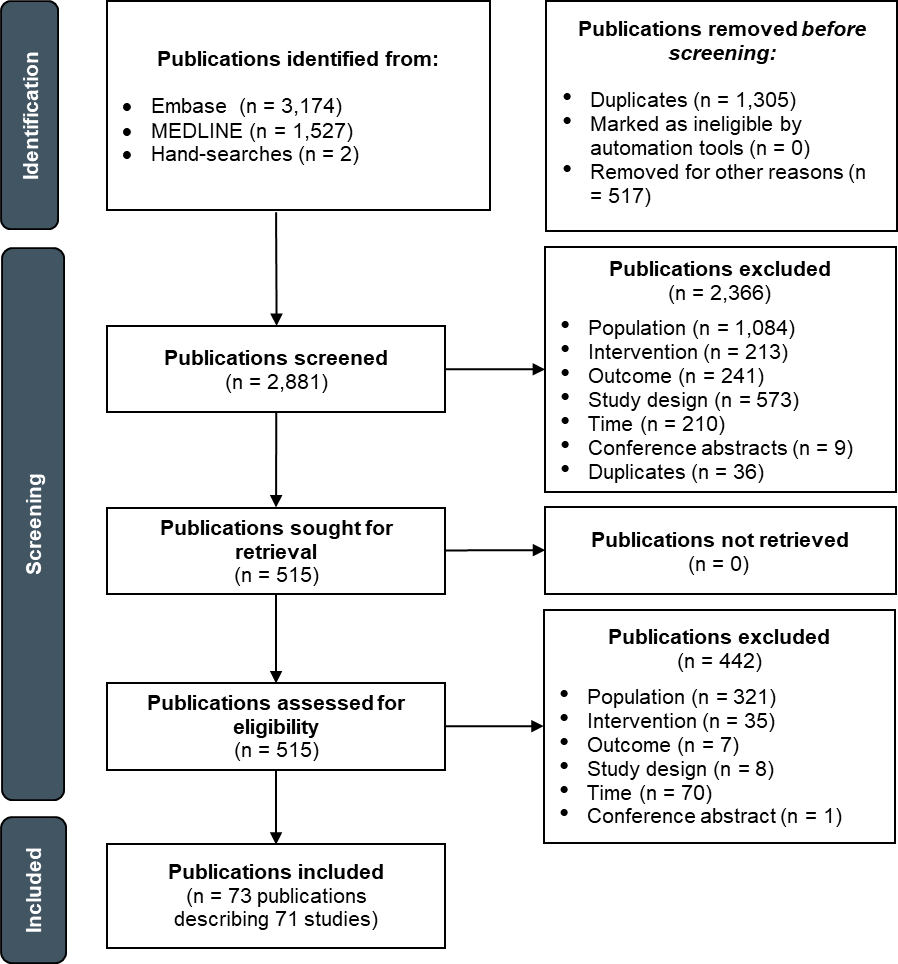


***Note:*** *“Removed for other reasons” refers to publications with irrelevant article types (e.g., conference abstracts, commentaries, review articles) according to metadata in the database export files.*

## Study Characteristics

Table S14: Study characteristics of advanced/metastatic NSCLC studies

| **Study ID** | **Study design** | **Country/region** | **Treatment (s) evaluated** | **N** |
| --- | --- | --- | --- | --- |
|  |  |  |  |  |
| **Pre-approval era** | | | | |
| Abernethy 2017 | Retrospective cohort | US | Platinum-based chemotherapy ± bevacizumab | 2014 |
| Banna 2017 | Prospective cohort | Italy | Cisplatin + pemetrexed, carboplatin + pemetrexed, carboplatin + paclitaxel + bevacizumab, carboplatin + paclitaxel, cisplatin + gemcitabine, carboplatin + gemcitabine | 38 |
| Bonanno 2017 | Retrospective cohort | Italy | Platinum doublet chemotherapy + bevacizumab | 125 |
| Camerini 2016 | Retrospective cohort | Italy | Bevacizumab + carboplatin + paclitaxel, bevacizumab + cisplatin + gemcitabine | 62 |
| Chen 2014 | Retrospective cohort | China | Icotinib | 82 |
| Elsamany 2015 | Retrospective cohort | Saudi Arabia | Erlotinib, non-pemetrexed-containing chemotherapy, pemetrexed-containing chemotherapy | 120 |
| Isobe 2017 | Retrospective cohort | Japan | Chemotherapy, TKI | 175 |
| Kohutek 2016 | Retrospective cohort | Slovakia | Gemcitabine, gemcitabine + carboplatin, gemcitabine + cisplatin | 56 |
| Mudad 2017 | Retrospective cohort | US | Nab-paclitaxel + carboplatin, gemcitabine+ cisplatin | 193 |
| Perez-Moreno 2016 | Retrospective cohort | Spain | Pemetrexed + platinum | 172 |
| de Castro 2017 | Retrospective cohort | Italy, Spain, Germany, Australia, Korea, Taiwan, Brazil | Chemotherapy | 1265 |
| Shen 2017 | Retrospective cohort | China | Pemetrexed + cisplatin, pemetrexed + carboplatin | 220 |
| Thippeswamy 2017 | Retrospective cohort | India | Pemetrexed/platinum, taxane/platinum, TKI/gefitinib, nab-paclitaxel/platinum | 202 |
| von Verschuer 2017 | Prospective cohort | Germany | Chemotherapy monotherapy, chemotherapy combination, TKI | 1239 |
| Xu 2017 | Retrospective cohort | China | Icotinib | 20 |
| **Post-approval era** | | | | |
| Aggarwal 2023 | Retrospective cohort | US | Pembrolizumab + pemetrexed + platinum | 2488 |
| Akazawa 2022 | Retrospective cohort | Japan | Nivolumab, pembrolizumab, atezolizumab ± chemotherapy | 260 |
| Attili 2022 | Retrospective cohort | Italy | Pembrolizumab + pemetrexed + platinum | 105 |
| Banna 2020 | Retrospective cohort | Italy, UK, Switzerland | Pembrolizumab | 132 |
| Bjornhart 2019 | Retrospective cohort | Denmark | Nivolumab, pembrolizumab | 118 |
| Bureau 2022 | Retrospective cohort | France | Pembrolizumab | 188 |
| Cavaille 2020 | Retrospective cohort | France | Pembrolizumab | 41 |
| Chang 2022 | Retrospective cohort | Taiwan | Pembrolizumab | 407 |
| Chen 2021 | Retrospective cohort | China | Pembrolizumab ± chemotherapy | 206 |
| Cortellini 2020 | Retrospective cohort | Italy | Pembrolizumab | 1026 |
| Descourt 2023 | Retrospective cohort | France | Pembrolizumab | 845 |
| Di Noia 2021 | Prospective cohort | Italy | Pembrolizumab | 42 |
| Dube-Pelletier 2023 | Retrospective cohort | Canada | Pembrolizumab | 160 |
| Dudnik 2018 | Retrospective cohort | Israel | Nivolumab monotherapy | 260 |
| Dudnik 2021 | Retrospective cohort | Israel | Pembrolizumab ± chemotherapy | 256 |
| Eklund 2021 | Retrospective cohort | Sweden | Pembrolizumab | 597 |
| Faoro 2023 | Retrospective cohort | Italy | Pembrolizumab | 98 |
| Frost 2021a | Retrospective cohort | Germany | Pembrolizumab | 153 |
| Frost 2021b | Retrospective cohort | Germany | Pembrolizumab | 119 |
| Fujimoto 2021 | Retrospective cohort | Japan | Pembrolizumab + chemotherapy | 299 |
| Geiger-Gritsch 2021 | Retrospective cohort | Austria | Pembrolizumab | 89 |
| Genova 2023 | Retrospective cohort | Italy | Pembrolizumab | 182 |
| Goto 2022 | Retrospective cohort | Japan | Pembrolizumab | 441 |
| Hasegawa 2020 | Retrospective cohort | Japan | Pembrolizumab | 30 |
| Holtzman 2022 | Retrospective cohort | Israel | Pembrolizumab, pembrolizumab + platinum-based chemotherapy | 423 |
| Hu 2022 | Retrospective cohort | China | Anti-PD-(L)1 monotherapy, anti-PD-(L)1 combination therapy | 201 |
| Ikeuchi 2023 | Retrospective cohort | Japan | Atezolizumab + bevacizumab + carboplatin + paclitaxel | 30 |
| Imai 2021 | Retrospective cohort | Japan | Pembrolizumab | 142 |
| Isono 2021 | Retrospective cohort | Japan | Pembrolizumab + chemotherapy, pembrolizumab | 71 |
| Ivanovic 2021 | Prospective cohort | Slovenia | Pembrolizumab | 66 |
| Kaira 2023 | Retrospective cohort | Japan | Pembrolizumab | 107 |
| Lang 2019 | Retrospective cohort | Austria | Nivolumab, pembrolizumab, or atezolizumab monotherapy | 153 |
| Lenci 2021 | Retrospective cohort | Europe | Pembrolizumab | 222 |
| Lester 2021 | Retrospective cohort | UK, Switzerland | Pembrolizumab, nivolumab, or atezolizumab | 1003 |
| Li 2022 | Retrospective cohort | China | Pembrolizumab + carboplatin + paclitaxel/pemetrexed | 80 |
| Liao 2021 | Retrospective cohort | China | Pembrolizumab + pemetrexed + cisplatin/carboplatin | 102 |
| Liu 2022 | Retrospective cohort | US | Pembrolizumab + pemetrexed + carboplatin | 377 |
| Liu 2023 | Retrospective cohort | US | Pembrolizumab + carboplatin + paclitaxel/nab-paclitaxel | 364 |
| Low 2021 | Retrospective cohort | Singapore | Pembrolizumab ± chemotherapy | 114 |
| Matsumoto 2022 | Retrospective cohort | Japan | Pembrolizumab, pembrolizumab + chemotherapy | 96 |
| Nindra 2023 | Retrospective cohort | Australia | Durvalumab | 145 |
| Nokihara 2022 | Retrospective cohort | Japan | Pembrolizumab, nivolumab, atezolizumab | 1208 |
| Noordhof 2021 | Retrospective cohort | Netherlands | Pembrolizumab | 595 |
| Amrane 2020 | Retrospective cohort | France | Pembrolizumab | 108 |
| Perol 2022 | Retrospective cohort | US | PD-(L)1 inhibitor monotherapy, PD-(L)1 inhibitor + platinum-doublet chemotherapy | 520 |
| Raez 2022 | Retrospective cohort | US, Mexico, Central America, Colombia | Durvalumab | 125 |
| Renaud 2023 | Retrospective cohort | France | Pembrolizumab + pemetrexed + carboplatin | 121 |
| Sanchez-Gastaldo 2021 | Retrospective cohort | Spain | Pembrolizumab | 51 |
| Seban 2020 | Retrospective cohort | France | Pembrolizumab | 63 |
| Seban 2021 | Retrospective cohort | France | Pembrolizumab | 90 |
| Shah 2022a | Retrospective cohort | US | PD-(L)1 inhibitor monotherapy | 166 |
| Shah 2022b | Retrospective cohort | US | Pembrolizumab | 1952 |
| Shah 2023 | Retrospective cohort | US | Pembrolizumab + carboplatin-based chemotherapy, pembrolizumab | 3086 |
| Shalata 2023 | Retrospective cohort | Israel | Pembrolizumab combination therapy, pembrolizumab monotherapy | 36 |
| Anpalakhan 2023a, Anpalakhan 2023b, Banna 2022 | Retrospective cohort | UK, Switzerland | Pembrolizumab + chemotherapy | 308 |
| Tamayo-Bermejo 2023 | Retrospective cohort | Spain | Pembrolizumab | 62 |
| Tamiya 2019 | Retrospective cohort | Japan | Pembrolizumab | 213 |
| Tang 2022 | Prospective cohort | Australia | ICI ± chemotherapy | 454 |
| Tibaldi 2022 | Retrospective cohort | Italy | Pembrolizumab | 205 |
| Tsai 2022 | Retrospective cohort | Taiwan | Pembrolizumab | 59 |
| Veccia 2021 | Retrospective cohort | Italy | Pembrolizumab | 117 |
| Velcheti 2019 | Retrospective cohort | US | Pembrolizumab | 611 |
| Velcheti 2021 | Retrospective cohort | US | Pembrolizumab + chemotherapy | 738 |
| Velcheti 2022 | Retrospective cohort | US | Pembrolizumab | 794 |
| Wang 2021 | Retrospective cohort | China | Anti-PD-(L)1 monotherapy | 325 |
| Waterhouse 2021 | Retrospective cohort | US | Atezolizumab/pembrolizumab combination therapy, atezolizumab/pembrolizumab monotherapy | 7312 |
| Xu 2022 | Retrospective cohort | China | Atezolizumab + carboplatin + nab-paclitaxel | 171 |
| Yoneda 2022 | Retrospective cohort | Japan | Nivolumab, pembrolizumab, atezolizumab | 435 |
| Yuasa 2023 | Retrospective cohort | Japan | ICI ± chemotherapy | 87 |
| Zayas-Soriano 2020 | Retrospective cohort | Spain | Pembrolizumab monotherapy | 80 |
| Zhang 2021 | Retrospective cohort | China | Pembrolizumab + chemotherapy | 66 |

**Abbreviations:** ICI, immune checkpoint inhibitor; PD-(L)1, programmed-death lignad-1; TKI, tyrosine kinase inhibitor; UK, United Kingdom; US, United States.

## Patient Characteristics

Table S15: Patient characteristics in advanced/metastatic NSCLC studies: age, sex, performance status, race, smoking status, and tumor histology

| **Study ID** | **Treatment** | **Overall/**  **subgroup** | **N** | **Age, years, median (range)** | **Male, %** | **ECOG PS**  **0-1, %** | **White race, %** | **Current/**  **former smoker, %** | **Histology** | |
| --- | --- | --- | --- | --- | --- | --- | --- | --- | --- | --- |
|  |  |  |  |  |  |  |  |  | **Squamous, %** | **Non-squamous, %^f^** |
| **Pre-approval era** | | | | | | | | |  |  |
| Abernethy 2017 | Chemotherapy | Overall | 2014 | 67.4 (10.1)^a^ | 55.1 | -- | 62.6 | -- | 21.6 | 78.4 |
| Banna 2017 | Doublet platinum-based chemotherapy | Overall | 38 | 67 (33-82) | 71 | 90 | -- | 85 | 16 | 66 |
| Bonanno 2017 | Platinum-based chemotherapy ± bevacizumab | Overall | 125 | 64 (58-71)^b^ | 68 | 95.2 | -- | 84 | 12 | 88 |
| Camerini 2016 | Doublet platinum-based chemotherapy + bevacizumab | Overall | 62 | 63.5 (30-77) | 62.9 | 90.3 | -- | 90.32 | 0 | 100 |
| Chen 2014 | Icotinib | 1L | 24 | 64 (37-79) | 37.5 | 29.1 | -- | 41.6 | 4.1 | 95.8 |
| Elsamany 2015 | Chemotherapy or erlotinib | Overall | 120 | -- | 76.7 | -- | -- | -- | 0 | 100 |
| Isobe 2017 | Chemotherapy | Squamous^e^ | 43 | 70 (57-86) | 86 | 85 | -- | 97 | 100 | 0 |
|  |  | Non-squamous, EGFR/ALK- positive^e^ | 46 | 71 (51-86) | 44 | 82 | -- | 54 | 0 | 100 |
|  |  | Overall^e^ | 175 | 70 (47-86) | 70 | 85 | -- | 81 | 24.5 | 73.7 |
|  |  | Non-squamous, EGFR/ALK- negative or unknown^e^ | 83 | 67 (47-81) | 77 | 54.2 | -- | 88 | 0 | 100 |
| Kohutek 2016 | Gemcitabine ± platinum | Overall | 56 | 65.54 (49-82) | 66 | 62.5 | -- | -- | 0 | 100 |
| Mudad 2017 | Nab-paclitaxel + carboplatin | Overall | 61 | 68.7 (--)^a^ | 66 | 28 | -- | -- | 100 | 0 |
|  | Gemcitabine + carboplatin | Overall | 132 | 67.9 (--)^a^ | 61 | 39 | -- | -- | 100 | 0 |
| Perez-Moreno 2016 | Pemetrexed + platinum | Overall | 172 | 63 (34-83) | 73.33 | 77.9 | -- | 78.49 | 1.74 | 98.26 |
| de Castro 2017 | Chemotherapy | Italy^e^ | 174 | 66.2 (--)^a^ | 70.7 | 50 | 99.4 | 67.9 | -- | -- |
|  |  | Spain^e^ | 202 | 62.9 (--)^a^ | 76.7 | 45 | 98.5 | 85.2 | -- | -- |
|  |  | Germany^e^ | 139 | 61.9 (--)^a^ | 53.2 | 71.2 | 100 | 68.3 | -- | -- |
|  |  | Australia^e^ | 208 | 63.9 (--)^a^ | 60.1 | 66.3 | 71.6 | 86.1 | -- | -- |
|  |  | Korea^e^ | 150 | 62.9 (--)^a^ | 69.3 | 40.7 | 0 | 63.3 | -- | -- |
|  |  | Taiwan^e^ | 217 | 65.1 (--)^a^ | 47.5 | 73.7 | 0 | 32.7 | -- | -- |
|  |  | Brazil^e^ | 175 | 63.3 (--)^a^ | 65.7 | 54.9 | 44 | 77.8 | -- | -- |
| Shen 2017 | Pemetrexed + cisplatin/carboplatin | Overall | 220 | 61 (30-78) | 52.7 | 100 | -- | 36.8 | 0 | 100 |
| Thippeswamy 2017 | Nab-paclitaxel/platinum doublet | Overall | 202 | 60.2 (29-92)^c^ | 66.33 | -- | -- | -- | 11.88 | 83.1 |
| von Verschuer 2017 | Chemotherapy combination or monotherapy or TKI | EGFR-negative | 430 | -- | -- | -- | -- | -- | -- | -- |
| Xu 2017a | Icotinib | Overall | 20^e^ | 62 (45-79) | 90 | 85 | -- | -- | 100 | 0 |
| **Post-approval era** | | | | | | | | |  |  |
| Aggarwal 2023 | Pembrolizumab + pemetrexed + platinum | No maintenance therapy | 276 | 66.75 (38.19-84.87) | 51.81 | 64.21 | 62.32 | 87.32 | 0 | 100 |
|  |  | Pembrolizumab ± pemetrexed maintenance therapy | 1091 | 68.53 (27.08-84.87) | 53.25 | 66 | 67.83 | 89.71 | 0 | 100 |
|  |  | Overall | 2488 | 68.09 (22.91-85.33) | 54.5 | 61.3 | 68.09 | 89.27 | 0 | 100 |
| Akazawa 2022 | Atezolizumab or pembrolizumab | 1L | 114 | 71.7 (70.1-73.3)^d^ | 79 | 83 | -- | 92 | -- | 53 |
| Attili 2022 | Pembrolizumab + pemetrexed + platinum | Overall | 49 | 68 (44-79) | 63.3 | 95.9 | -- | 89.8 | 0 | 100 |
| Banna 2020 | Pembrolizumab | Overall | 132 | 68 (31-85) | 66 | 83.3 | -- | 89 | 27 | 65 |
| Bjornhart 2019 | Pembrolizumab or nivolumab | Overall^e^ | 118 | 66 (59-71)^b^ | 47 | 90 | -- | 95 | 23 | 77 |
| Bureau 2022 | Pembrolizumab | Overall | 96 | 63 (38-89) | 67.7 | 77.9 | -- | 95.6 | 19.8 | 80.2 |
| Cavaille 2020 | Pembrolizumab | Overall | 41 | 64 (33-79) | 48.8 | 73.1 | -- | -- | 12.2 | 87.8 |
| Chang 2022 | Pembrolizumab | Overall | 242^e^ | 62 (56-72) | 63.2 | 86.8 | -- | -- | 24.4 | 75.6 |
| Chen 2021 | Pembrolizumab | Overall | 91 | 67 (29-87) | 87.9 | -- | -- | 79.1 | 46.2 | 53.8 |
|  | Pembrolizumab + chemotherapy | Overall | 115 | 65 (37-76) | 88.7 | -- | -- | 74.8 | 35.7 | 64.3 |
| Cortellini 2020 | Pembrolizumab | Overall | 1026 | 70.2 (28-92) | 65.6 | 82.6 | -- | 89.7 | 24.2 | 75.8 |
| Descourt 2023 | Pembrolizumab | Overall | 845 | 65 (59-72) | -- | 78.1 | -- | 93.4 | -- | 70.2 |
| Di Noia 2021 | Pembrolizumab | Overall | 42 | 70.5 (35-86) | 71 | 95.2 | -- | 88 | 14 | 86 |
| Dube-Pelletier 2023 | Pembrolizumab Q6W | Overall | 80 | 70 (7.7)^a^ | 44 | 76 | -- | 99 | 15 | 77 |
|  | Pembrolizumab Q3W | Overall | 80 | 66 (7.9)^a^ | 40 | 77 | -- | 95 | 12 | 77 |
| Dudnik 2018 | Nivolumab monotherapy | Overall^e^ | 260 | 67 (41-99) | 68 | 46 | -- | -- | 23 | 70 |
| Dudnik 2021 | Pembrolizumab | Overall | 203 | 68.4 (10.6)^a^ | 68 | 68 | -- | 91 | 16 | 78 |
|  | Pembrolizumab + chemotherapy | Overall | 53 | 64.3 (9.8)^a^ | 58 | 85 | -- | 89 | 19 | 72 |
| Eklund 2021 | Pembrolizumab | KRAS WT | 17 | 70 (57-83) | 35.3 | 64.7 | -- | 94.1 | 5.9 | 82.4 |
|  | Pembrolizumab | KRAS mutation | 20 | 69 (46-78) | 60 | 85 | -- | 95 | 0 | 95 |
| Faoro 2023 | Pembrolizumab | Overall | 98 | 73 (44-89) | 64.3 | 90.8 | -- | -- | -- | -- |
| Frost 2021a | Pembrolizumab | Overall | 153 | 69 (40-86) | 58.8 | 75.2 | -- | 92.6 | 20.9 | 70.0 |
| Frost 2021b | Pembrolizumab | Overall | 119 | 68 (40-86) | 57.8 | 77.3 | -- | 91.6 | -- | 89.9 |
| Fujimoto 2021 | Pembrolizumab + chemotherapy | Overall | 299 | 65.7 (9.5)^a^ | 74 | 95 | -- | 84 | -- | 100 |
| Geiger-Gritsch 2021 | Pembrolizumab | 1L | 42 | 67.5 (49-85) | 61.9 | 90.5 | -- | 95.2 | 19.1 | 80.9 |
| Genova 2023 | Pembrolizumab | 1L | 56 | 70.1 (50.5-88.8) | 76.8 | 82.1 | -- | 92.9 | 25 | 75 |
| Goto 2022 | Pembrolizumab | Overall | 441 | 70 (30-89) | 78.5 | 54.4 | -- | 89 | 29.3 | 70.7 |
| Hasegawa 2020 | Pembrolizumab | Overall | 30 | 71 (51-82) | 76.7 | 93.3 | -- | 96.7 | 30 | 70 |
| Holtzman 2022 | Pembrolizumab | Overall | 302 | 70 (36-97) | 66 | 66 | -- | 89 | 17 | 75 |
|  | Pembrolizumab + platinum-based chemotherapy | Overall | 121 | 66 (35-87) | 61 | 80 | -- | 89 | 24 | 64 |
| Hu 2022 | Anti-PD-(L)1 monotherapy or combination therapy | Overall^e^ | 201 | -- | -- | -- | -- | -- | 0 | 100 |
| Ikeuchi 2023 | Atezolizumab + bevacizumab + carboplatin + paclitaxel | Overall | 30 | 69 (45-75) | 80 | 96.6 | -- | 76.7 | 0 | 100 |
| Imai 2021 | Pembrolizumab | Overall | 142 | 70 (47-86) | 82.4 | 77.5 | -- | 91.5 | 28.2 | 71.8 |
| Isono 2021 | Pembrolizumab + chemotherapy | Overall | 33 | 66 (44-77) | 93.9 | 100 | -- | 90.9 | 21.2 | 63.6 |
|  | Pembrolizumab | Overall | 38 | 72 (57-82) | 73.7 | 84.2 | -- | 86.8 | 21.1 | 57.9 |
| Ivanovic 2021 | Pembrolizumab | 1L | 26 | 65.5 (39-78) | 62 | 89 | -- | 77 | 8 | 81 |
| Kaira 2023 | Pembrolizumab | Overall | 107 | 71 (--) | 85 | 76.6 | -- | 89.7 | 42.1 | 54.9 |
| Lang 2019 | Nivolumab, pembrolizumab, or atezolizumab | 1L | 45 | 72 (47-90)^c^ | 58 | 96 | -- | 89 | 58 | 42 |
| Lenci 2021 | Pembrolizumab | Overall | 135 | 71 (44-91) | 62 | 76 | -- | 86 | -- | 84 |
| Lester 2021 | Pembrolizumab, nivolumab, or atezolizumab | Overall | 179 | 67 (48-90) | 52.5 | 87.7 | -- | -- | 21.2 | 74.3 |
| Li 2022 | Pembrolizumab + carboplatin + paclitaxel/pemetrexed | Overall | 80 | 63 (28-85) | 88.8 | 100 | -- | 72.6 | 31.3 | 68.8 |
| Liao 2021 | Pembrolizumab + pemetrexed + cisplatin/carboplatin | Overall | 49 | 64 (59-67)^b^ | 79.6 | 100 | -- | 75.5 | 0 | 100 |
| Liu 2022 | Pembrolizumab + pemetrexed + carboplatin | Overall | 377 | 66 (29-83) | 60.2 | 100 | 76.4 | 91.5 | 0 | 100 |
| Liu 2023 | Pembrolizumab + carboplatin + paclitaxel/nab-paclitaxel | Overall | 364 | 70 (43-84) | 66.8 | 100 | 78.5 | 95.3 | 100 | 0 |
| Low 2021 | Pembrolizumab 200mg ± chemotherapy | Overall | 49 | 60.5 (28.4-80) | 84 | 86 | -- | 65 | 14 | 76 |
|  | Pembrolizumab 100mg ± chemotherapy | Overall | 65 | 69.9 (42.8-92.2) | 69 | 71 | -- | 75 | 9 | 74 |
| Matusomoto 2022 | Pembrolizumab | Overall | 47 | 71 (36-87) | 78.7 | 100 | -- | 83 | 38.3 | 61.7 |
|  | Pembrolizumab combination therapy | Overall | 49 | 68 (52-81) | 83.6 | 100 | -- | 85.7 | 24.5 | 75.5 |
| Nindra 2023 | Durvalumab monotherapy | EGFR WT | 130 | 66 (46-84)^c^ | 66 | 96 | -- | 92 | 34 | 65 |
| Nokihara 2022 | Pembrolizumab, nivolumab, or atezolizumab | Overall | 463 | 70 (30-89) | 78.2 | 52.3 | -- | 89.3 | 30 | 70 |
| Noordhof 2021 | Pembrolizumab | Overall | 595 | 65 (59-72)^b^ | 49.7 | 77.3 | -- | -- | 0 | 100 |
| Amrane 2020 | Pembrolizumab | Overall | 108 | 67 (37-87) | 64.8 | 64.8 | -- | 88.9 | 25.9 | 74.1 |
| Perol 2022 | PD-(L)1 inhibitor + platinum-doublet chemotherapy | Overall | 169 | 67 (59-74)^b^ | 56 | 100 | 69 | 91 | 0 | 100 |
|  | PD-(L)1 inhibitor | Overall | 351 | 72 (64-79)^b^ | 48 | 100 | 70 | 90 | 0 | 100 |
| Raez 2022 | Durvalumab | Overall | 125 | 66 (41-90) | 47.2 | 100 | 36 | 83.2 | 20 | 80 |
| Renaud 2023 | Pembrolizumab + pemetrexed + carboplatin | Overall | 121 | 59.8 (--) | -- | 91.7 | -- | 95 | 0 | 100 |
| Sanchez-Gastaldo 2021 | Pembrolizumab | Overall | 51 | 66 (46-85) | 72.55 | 76.47 | -- | 70.59 | -- | 62.74 |
| Seban 2020 | Pembrolizumab | Overall | 63 | 65 (37-86) | 60.3 | -- | -- | 95.2 | 3 | 14 |
| Seban 2021 | Pembrolizumab | Overall | 51 | 65 (37-86) | 61 | -- | -- | 98 | 24 | 76 |
| Shah 2022a | PD-(L)1 inhibitor | Very high PD-L1 expression | 62 | 68 (62-75)^b^ | 48 | 73 | 76 | 92 | 21 | 73 |
|  |  | High PD-L1 expression | 104 | 67 (61-76)^b^ | 45 | 72 | 66 | 95 | 20 | 69 |
| Shah 2022b | Pembrolizumab | Very high PD-L1 expression | 891 | 73 (65-79)^b^ | 49 | 57 | 70 | 94 | 20 | 75 |
|  |  | High PD-L1 expression | 1061 | 74 (66-80)^b^ | 51 | 58 | 69 | 94 | 29 | 67 |
| Shah 2023 | Pembrolizumab + carboplatin-based chemotherapy | Overall | 998 | 68 (61-75) | 56 | 62 | 68 | 93 | 17 | 78 |
|  | Pembrolizumab monotherapy | Overall | 2088 | 73 (65-80) | 50 | 55 | 70 | 94 | 25 | 71 |
| Shalata 2023 | Pembrolizumab combination therapy or monotherapy | Overall | 36 | 65.1 (46-83) | 63.9 | 100 | -- | 83.2 | 31.6 | 68.3 |
| Anpalakhan 2023a, Anpalakhan 2023b, Banna 2022 | Pembrolizumab + chemotherapy | Overall | 308 | 65 (37-84) | 56 | 100 | -- | 92 | 17 | 83 |
| Tamayo 2023 | Pembrolizumab | 1L | 33 | 64 (40-75) | 78.79 | 87.88 | -- | 87.88 | 39.39 | 60.61 |
| Tamiya 2019 | Pembrolizumab | Overall | 213 | 71 (39-91) | 82.6 | 80.7 | -- | 90.6 | 25.8 | 74.2 |
| Tang 2022 | ICI ± chemotherapy | Overall^e^ | 454 | 71 (61-79)^b^ | 57.9 | 78.6 | -- | 76.7 | 11.9 | 82.2 |
| Tibaldi 2022 | Pembrolizumab | Overall | 205 | 70 (38-86) | 65.8 | 77 | 100 | 85.9 | -- | 78.5 |
| Tsai 2022 | Pembrolizumab | Overall | 28 | 73 (66-80) | 75 | 85.7 | -- | 50 | 21.4 | 78.6 |
| Veccia 2021 | Pembrolizumab | Overall | 117 | 69 (61-74) | 66.7 | 85.5 | -- | 92.3 | 19.7 | 83.3 |
| Velcheti 2019 | Pembrolizumab | Spotlight cohort | 188 | 72 (46-84) | 47.9 | 100 | 77 | 91 | 24.5 | 68.6 |
|  | Pembrolizumab | EHR cohort | 423 | 72 (45-84) | 53.9 | 100 | 78.3 | 92.2 | 23.4 | 71.4 |
| Velcheti 2021 | Pembrolizumab + carboplatin + pemetrexed | Overall | 283 | 66 (33-84) | 59.4 | 100 | 74 | 91.9 | -- | -- |
| Velcheti 2022 | Pembrolizumab | Spotlight cohort | 228 | 71 (46-82) | 46.1 | 100 | 78.9 | 91.7 | 26.3 | 68.4 |
|  | Pembrolizumab | EHR cohort | 566 | 71 (38-84) | 52.7 | 100 | 75.8 | 92.6 | 23.5 | 71.6 |
| Wang 2021 | Anti-PD-(L)1 monotherapy | Overall | 178 | 63.3 (8.5)^a^ | 82.6 | 100 | -- | 69.4 | 33.7 | 61.3 |
| Waterhouse 2021 | Atezolizumab or pembrolizumab monotherapy or combination therapy | Overall | 4271 | 69 (62-75)^b^ | 57 | 63 | 67 | 91 | 19 | 81 |
|  | Atezolizumab or pembrolizumab combination therapy | Squamous | 814 | 70 (64-76)^b^ | 55 | 64 | 69 | 97 | 100 | 0 |
|  |  | Non-squamous | 3457 | 69 (61-75)^b^ | 68 | 63 | 67 | 90 | 0 | 100 |
|  | Atezolizumab or pembrolizumab monotherapy | Squamous | 875 | 74 (67-80)^b^ | 61 | 54 | 72 | 95 | 100 | 0 |
|  |  | Non-squamous | 2166 | 73 (65-80)^b^ | 46 | 52 | 70 | 92 | 0 | 100 |
| Xu 2022 | Atezolizumab + carboplatin + nab-paclitaxel | Overall | 60 | 64 (46-75) | 68.3 | 100 | -- | 90 | -- | -- |
| Yoneda 2022 | Nivolumab, pembrolizumab, or atezolizumab monotherapy | Overall^e^ | 435 | 69 (30-90) | 79 | 82.2 | -- | -- | -- | 59.8 |
| Yuasa 2023 | ICI ± chemotherapy | 1L | 35 | 72 (67-77)^b^ | 85.7 | 97.1 | -- | 85.7 | 28.6 | 60 |
| Zayas-Soriano 2020 | Pembrolizumab monotherapy | 1L | 32 | 67.2 (1.4)^a^ | 71.9 | 81.3 | -- | 93.7 | 28.1 | 62.6 |
| Zhang 2021 | Pembrolizumab + cisplatin + pemetrexed | Overall | 34 | 62 (47-72) | 76.5 | 100 | -- | 55.9 | 0 | 100 |

**Notes: a)** Mean (standard deviation). **b)** Median (interquartile range). **c)** Mean (range). **d)** Mean (95% confidence interval). **e)** Patient characteristics only reported for first-line and subsequent therapy combined. f**)** The “non-squamous” category consisted of adenocarcinoma, large cell carcinoma, and/or “other” tumor histology. Patients reported as having “not otherwise specified” tumor histology were not included in the “squamous” or “non-squamous” categories. **Abbreviations:** ALK, anaplastic lymphoma kinase; ECOG, Eastern Cooperative Oncology Group; EGFR, epithelial growth factor receptor; EHR, electronic health record; ICI, immune checkpoint inhibitor; KRAS, Kirsten rat sarcoma virus; L, line; PD-(L)1, programmed death ligand-1; PS, performance status; Q3W, every 3 weeks; Q6W, every 6 weeks; TKI, tyrosine kinase inhibitor; WHO, World Health Organization; WT, wild-type.

Table S16: Patient characteristics in advanced/metastatic NSCLC studies: EGFR/ALK/ROS status and PD-L1 expression

| **Study ID** | **Treatment** | **Overall/**  **subgroup** | **N** | **EGFR/ALK/ROS1 status** | | | | **PD-L1 expression (TPS)** | | | | |
| --- | --- | --- | --- | --- | --- | --- | --- | --- | --- | --- | --- | --- |
|  |  |  |  | **EGFR alteration, %** | **ALK alteration, %** | **EGFR/**  **ALK alteration, %** | **ROS1 alteration, %** | **<1%, %** | | **≥1%, %** | **1-49%, %** | **≥50%, %** |
| **Pre-approval era** | | | | | | | | | | | | |
| Abernethy 2017 | Chemotherapy | Overall | 2014 | -- | -- | -- | -- | | -- | -- | -- | -- |
| Banna 2017 | Doublet platinum-based chemotherapy | Overall | 38 | 16 | -- | -- | -- | | -- | -- | -- | -- |
| Bonanno 2017 | Platinum-based chemotherapy ± bevacizumab | Overall | 125 | 4 | 4.8 | -- | -- | | -- | -- | -- | -- |
| Camerini 2016 | Doublet platinum-based chemotherapy + bevacizumab | Overall | 62 | 7 | 5 | -- | -- | | -- | -- | -- | -- |
| Chen 2014 | Icotinib | 1L | 24 | 33.3 | -- | -- | -- | | -- | -- | -- | -- |
| Elsamany 2015 | Chemotherapy or erlotinib | Overall | 120 | 17.5 | -- | -- | -- | | -- | -- | -- | -- |
| Isobe 2017 | Chemotherapy | Squamous^a^ | 43 | 6 | 0 | -- | -- | | -- | -- | -- | -- |
|  |  | Non-squamous, EGFR/ALK-positive^a^ | 46 | -- | -- | 100 | -- | | -- | -- | -- | -- |
|  |  | Overall^a^ | 175 | 38 | 7 | -- | -- | | -- | -- | -- | -- |
|  |  | Non-squamous, EGFR/ALK-negative or unknown^a^ | 83 | -- | -- | 0 | -- | | -- | -- | -- | -- |
| Kohutek 2016 | Gemcitabine ± platinum | Overall | 56 | 0 | -- | -- | -- | | -- | -- | -- | -- |
| Mudad 2017 | Nab-paclitaxel + carboplatin | Overall | 61 | -- | -- | -- | -- | | -- | -- | -- | -- |
|  | Gemcitabine + carboplatin | Overall | 132 | -- | -- | -- | -- | | -- | -- | -- | -- |
| Perez-Moreno 2016 | Pemetrexed + platinum | Overall | 172 | 5.81 | -- | -- | -- | | -- | -- | -- | -- |
| de Castro 2017 | Chemotherapy | Italy^a^ | 174 | 25.6 | 3 | 28.6 | -- | | -- | -- | -- | -- |
|  |  | Spain^a^ | 202 | 15.6 | 6.7 | 22.3 | -- | | -- | -- | -- | -- |
|  |  | Germany^a^ | 139 | 26.8 | 4.5 | 31.3 | -- | | -- | -- | -- | -- |
|  |  | Australia^a^ | 208 | 22 | 14.8 | 36.8 | -- | | -- | -- | -- | -- |
|  |  | Korea^a^ | 150 | 40.7 | 15.8 | 56.5 | -- | | -- | -- | -- | -- |
|  |  | Taiwan^a^ | 217 | 68 | 60 | 128 | -- | | -- | -- | -- | -- |
|  |  | Brazil^a^ | 175 | 17.8 | 0 | 17.8 | -- | | -- | -- | -- | -- |
| Shen 2017 | Pemetrexed + cisplatin/carboplatin | Overall | 220 | 11.4 | 4.1 | 15.5 | -- | | -- | -- | -- | -- |
| Thippeswamy 2017 | Nab-paclitaxel/platinum doublet | Overall | 202 | 19.3 | 0.99 | -- | -- | | -- | -- | -- | -- |
| von Verschuer 2017 | Chemotherapy combination or monotherapy or TKI | EGFR negative | 430 | -- | -- | -- | -- | | -- | -- | -- | -- |
| Xu 2017 | Icotinib | Overall^a^ | 20 | 10 | -- | -- | -- | | -- | -- | -- | -- |
| **Post-approval era** | | | | | | | | | | | | |
| Aggarwal 2023 | Pembrolizumab + pemetrexed + platinum | No maintenance therapy | 276 | 4.35 | 0.36 | 4.71 | 0 | | 34.42 | 42.39 | 25 | 17.39 |
|  | Pembrolizumab + pemetrexed + platinum | Pembrolizumab ± pemetrexed maintenance therapy | 1091 | 3.02 | 0.64 | 3.66 | 0.17 | | 32.63 | 42.53 | 22.18 | 20.35 |
|  | Pembrolizumab + pemetrexed + platinum | Overall | 2488 | 3.46 | 0.52 | 3.98 | 0.24 | | 33.48 | 40.71 | 21.86 | 18.85 |
| Akazawa 2022 | Atezolizumab or pembrolizumab | 1L | 114 | 7.9 | -- | -- | -- | | -- | -- | -- | -- |
| Attili 2022 | Pembrolizumab + pemetrexed + platinum | Overall | 49 | -- | -- | 0 | -- | | 32.7 | 67.3 | 67.3 | -- |
| Banna 2020 | Pembrolizumab | Overall | 132 | 4 | 0 | 4 | 0 | | -- | -- | -- | 100 |
| Bjornhart 2019 | Pembrolizumab or nivolumab | Overall^a^ | 118 | 3 | 0 | -- | -- | | 3 | 82 | 14 | 68 |
| Bureau 2022 | Pembrolizumab | Overall | 96 | 0 | -- | -- | -- | | -- | -- | -- | 100 |
| Cavaille 2020 | Pembrolizumab | Overall | 41 | 0 | -- | -- | -- | | -- | -- | -- | 100 |
| Chang 2022 | Pembrolizumab | Overall^a^ | 242 | -- | -- | -- | -- | | 11.6 | -- | 21.9 | 47.1 |
| Chen 2021 | Pembrolizumab | Overall | 91 | 0 | 0 | 0 | -- | | -- | -- | -- | 100 |
|  | Pembrolizumab + chemotherapy | Overall | 115 | 0 | 0 | 0 | -- | | -- | -- | -- | 100 |
| Cortellini 2020 | Pembrolizumab | Overall | 1026 | 0.9 | -- | -- | -- | | -- | -- | -- | 100 |
| Descourt 2023 | Pembrolizumab | Overall | 845 | 0 | 0 | 0 | -- | | -- | -- | -- | 100 |
| Di Noia 2021 | Pembrolizumab | Overall | 42 | 0 | 0 | 0 | -- | | -- | -- | -- | -- |
| Dube-Pelletier 2023 | Pembrolizumab Q6W | Overall | 80 | 0 | 0 | 0 | 0 | | -- | -- | -- | 100 |
|  | Pembrolizumab Q3W | Overall | 80 | 1 | 0 | 1 | 0 | | -- | 100 | 9 | 91 |
| Dudnik 2018 | Nivolumab monotherapy | Overall^a^ | 260 | 5 | 0 | 5 | -- | | -- | -- | -- | -- |
| Dudnik 2021 | Pembrolizumab | Overall | 203 | -- | -- | -- | -- | | -- | -- | -- | 100 |
|  | Pembrolizumab + chemotherapy | Overall | 53 | -- | -- | -- | -- | | -- | -- | -- | 100 |
| Eklund 2021 | Pembrolizumab | KRAS WT | 17 | 5.9 | 0 | 5.9 | 0 | | -- | 94.1 | 17.6 | 76.5 |
|  | Pembrolizumab | KRAS mutation | 20 | -- | -- | -- | -- | | -- | 100 | 0 | 100 |
| Faoro 2023 | Pembrolizumab | Overall | 98 | 0 | 0 | 0 | -- | | -- | -- | -- | 100 |
| Frost 2021a | Pembrolizumab | Overall | 153 | 0 | 0 | 0 | 0 | | -- | -- | -- | 100 |
| Frost 2021b | Pembrolizumab | Overall | 119 | 0 | 0 | 0 | 0 | | -- | -- | -- | 100 |
| Fujimoto 2021 | Pembrolizumab + chemotherapy | Overall | 299 | 0 | 0 | 0 | 0 | | 37 | 56 | 35 | 21 |
| Geiger-Gritsch 2021 | Pembrolizumab | 1L | 42 | 0 | 0 | 0 | 0 | | -- | 100 | 2.4 | 97.6 |
| Genova 2023 | Pembrolizumab | 1L | 56 | -- | -- | -- | -- | | -- | -- | -- | -- |
| Goto 2022 | Pembrolizumab | Overall | 441 | 0 | 0 | 0 | -- | | -- | -- | -- | -- |
| Hasegawa 2020 | Pembrolizumab | Overall | 30 | -- | -- | -- | -- | | -- | -- | -- | -- |
| Holtzman 2022 | Pembrolizumab | Overall | 302 | 0 | 0 | 0 | 0 | | -- | -- | -- | 100 |
|  | Pembrolizumab + platinum-based chemotherapy | Overall | 121 | 0 | 0 | 0 | 0 | | -- | -- | -- | 100 |
| Hu 2022 | Anti-PD-(L)1 monotherapy or combination therapy | Overall^a^ | 201 | 13.9 | -- | -- | -- | | -- | -- | -- | -- |
| Ikeuchi 2023 | Atezolizumab + bevacizumab + carboplatin + paclitaxel | Overall | 30 | 23.3 | -- | -- | -- | | 20 | 66.7 | 26.7 | 40 |
| Imai 2021 | Pembrolizumab | Overall | 142 | 0 | 0 | 0 | -- | | -- | -- | -- | 100 |
| Isono 2021 | Pembrolizumab + chemotherapy | Overall | 33 | 0 | 0 | 0 | 0 | | 30.3 | 54.5 | 21.2 | 33.3 |
|  | Pembrolizumab | Overall | 38 | 0 | 0 | 0 | 0 | | -- | 100 | 2.6 | 97.4 |
| Ivanovic 2021 | Pembrolizumab | 1L | 26 | 0 | 0 | 0 | 0 | | -- | -- | -- | 100 |
| Kaira 2023 | Pembrolizumab | Overall | 107 | -- | -- | -- | -- | | -- | 100 | 24.3 | 75.7 |
| Lang 2019 | Nivolumab, pembrolizumab, or atezolizumab | 1L | 45 | -- | -- | -- | -- | | 29 | 65 | 29 | 36 |
| Lenci 2021 | Pembrolizumab | Overall | 135 | 0 | 0 | 0 | -- | | -- | -- | -- | 100 |
| Lester 2021 | Pembrolizumab, nivolumab, or atezolizumab | Overall | 179 | 0 | 0 | 0 | -- | | -- | 100 | -- | -- |
| Li 2022 | Pembrolizumab + carboplatin + paclitaxel/pemetrexed | Overall | 80 | 0 | 0 | 0 | -- | | 32.5 | 67.6 | 53.8 | 13.8 |
| Liao 2021 | Pembrolizumab + pemetrexed + cisplatin/carboplatin | Overall | 49 | 0 | 0 | 0 | -- | | 18.4 | 44.9 | 18.4 | 26.5 |
| Liu 2022 | Pembrolizumab + pemetrexed + carboplatin | Overall | 377 | 0 | 0 | 0 | -- | | 27.3 | 55.5 | 27.6 | 27.9 |
| Liu 2023 | Pembrolizumab + carboplatin + paclitaxel/nab-paclitaxel | Overall | 364 | -- | -- | -- | -- | | 25.8 | 47.3 | -- | -- |
| Low 2021 | Pembrolizumab 200mg ± chemotherapy | Overall | 49 | 4 | 0 | 4 | -- | | 22 | 76 | 37 | 39 |
|  | Pembrolizumab 100mg ± chemotherapy | Overall | 65 | 8 | 2 | 10 | -- | | 8 | 88 | 20 | 68 |
| Matusomoto 2022 | Pembrolizumab | Overall | 47 | 0 | 0 | 0 | -- | | 0 | 100 | 2.1 | 97.9 |
|  | Pembrolizumab combination therapy | Overall | 49 | 0 | 0 | 0 | -- | | 20.4 | 61.2 | 20.4 | 40.8 |
| Nindra 2023 | Durvalumab monotherapy | EGFR WT | 130 | 0 | 0 | 0 | 0 | | 45 | 55 | -- | -- |
| Nokihara 2022 | Pembrolizumab, nivolumab, or atezolizumab | Overall | 463 | 0 | 0 | 0 | 0 | | 0.2 | 99.5 | 1.7 | 97.8 |
| Noordhof 2021 | Pembrolizumab | Overall | 595 | 0 | 0 | 0 | 0 | | -- | -- | -- | 100 |
| Amrane 2020 | Pembrolizumab | Overall | 108 | 0 | 0 | 0 | 0.9 | | -- | -- | -- | 100 |
| Perol 2022 | PD-(L)1 inhibitor + platinum-doublet chemotherapy | Overall | 169 | 0 | 0 | 0 | 0 | | -- | -- | -- | 100 |
|  | PD-(L)1 inhibitor | Overall | 351 | 0 | 0 | 0 | 0 | | -- | -- | -- | 100 |
| Raez 2022 | Durvalumab | Overall | 125 | 4.8 | -- | -- | -- | | 28 | 67.2 | 52 | 15.2 |
| Renaud 2023 | Pembrolizumab + pemetrexed + carboplatin | Overall | 121 | 3.3 | -- | -- | 0.8 | | 44.6 | 49.6 | 28.1 | 21.5 |
| Sanchez-Gastaldo 2021 | Pembrolizumab | Overall | 51 | 0 | 0 | 0 | -- | | -- | -- | -- | 100 |
| Seban 2020 | Pembrolizumab | Overall | 63 | 0 | 0 | 0 | -- | | -- | -- | -- | 100 |
| Seban 2021 | Pembrolizumab | Overall | 51 | 0 | 0 | 0 | -- | | -- | -- | -- | 100 |
| Shah 2022a | PD-(L)1 inhibitor | Very high PD-L1 expression | 62 | 0 | 0 | 0 | -- | | -- | -- | -- | 100 |
|  |  | High PD-L1 expression | 104 | 0 | 0 | 0 | -- | | -- | -- | -- | 100 |
| Shah 2022b | Pembrolizumab | Very high PD-L1 expression | 891 | 0 | 0 | 0 | -- | | -- | -- | -- | 100 |
|  |  | High PD-L1 expression | 1061 | 0 | 0 | 0 | -- | | -- | -- | -- | 100 |
| Shah 2023 | Pembrolizumab + carboplatin-based chemotherapy | Overall | 998 | 0 | 0 | 0 | -- | | -- | -- | -- | 100 |
|  | Pembrolizumab monotherapy | Overall | 2088 | 0 | 0 | 0 | -- | | -- | -- | -- | 100 |
| Shalata 2023 | Pembrolizumab combination therapy or monotherapy | Overall | 36 | 0 | 0 | 0 | 0 | | 11.1 | 66.7 | 16.7 | 50 |
| Anpalakhan 2023a, Anpalakhan 2023b, Banna 2022 | Pembrolizumab + chemotherapy | Overall | 308 | 0 | 0 | 0 | 0 | | -- | -- | -- | -- |
| Tamayo 2023 | Pembrolizumab | 1L | 33 | 0 | 0 | 0 | -- | | -- | 100 | -- | -- |
| Tamiya 2019 | Pembrolizumab | Overall | 213 | 2.8 | -- | -- | -- | | -- | -- | -- | 100 |
| Tang 2022 | ICI ± chemotherapy | Overall^a^ | 454 | 18.3 | 4.8 | -- | -- | | -- | -- | -- | -- |
| Tibaldi 2022 | Pembrolizumab | Overall | 205 | 0 | 0 | 0 | 0 | | -- | -- | -- | 100 |
| Tsai 2022 | Pembrolizumab | Overall | 28 | 0 | 0 | 0 | 0 | | -- | -- | -- | -- |
| Veccia 2021 | Pembrolizumab | Overall | 117 | 0 | 0 | 0 | 0 | | -- | -- | -- | 100 |
| Velcheti 2019 | Pembrolizumab | Spotlight cohort | 188 | 0 | 0 | 0 | 0 | | -- | -- | -- | 100 |
|  | Pembrolizumab | EHR cohort | 423 | 0 | 0 | 0 | 0 | | -- | -- | -- | 100 |
| Velcheti 2021 | Pembrolizumab + carboplatin + pemetrexed | Overall | 283 | 0 | 0 | 0 | -- | | -- | -- | -- | -- |
| Velcheti 2022 | Pembrolizumab | Spotlight cohort | 228 | 0 | 0 | 0 | -- | | -- | -- | -- | 100 |
|  |  | EHR cohort | 566 | 0 | 0 | 0 | -- | | -- | -- | -- | 100 |
| Wang 2021 | Anti-PD-(L)1 monotherapy | Overall | 178 | 12.9 | -- | -- | -- | | -- | -- | -- | -- |
| Waterhouse 2021 | Atezolizumab or pembrolizumab monotherapy or combination therapy | Overall | 4271 | 0 | 0 | 0 | -- | | 30 | 48 | 29 | 19 |
|  | Atezolizumab or pembrolizumab combination therapy | Squamous | 814 | 0 | 0 | 0 | -- | | 26 | 46 | 31 | 15 |
|  |  | Non-squamous | 3457 | 0 | 0 | 0 | -- | | 31 | 48 | 28 | 20 |
|  | Atezolizumab or pembrolizumab monotherapy | Squamous | 875 | 0 | 0 | 0 | -- | | 6 | 79 | 18 | 61 |
|  |  | Non-squamous | 2166 | 0 | 0 | 0 | -- | | 5 | 84 | 11 | 73 |
| Xu 2022 | Atezolizumab + carboplatin + nab-paclitaxel | Overall | 60 | 0 | 0 | 0 | -- | | -- | 100 | -- | -- |
| Yoneda 2022 | Nivolumab, pembrolizumab, or atezolizumab monotherapy | Overall^a^ | 435 | 5.3 | 0.9 | -- | -- | | -- | -- | -- | 34 |
| Yuasa 2023 | ICI ± chemotherapy | 1L | 35 | 0 | 0 | -- | 2.9 | | -- | 71.4 | 45.7 | 25.7 |
| Zayas-Soriano 2020 | Pembrolizumab monotherapy | 1L | 32 | 0 | 0 | 0 | 0 | | -- | -- | -- | 75 |
| Zhang 2021 | Pembrolizumab + cisplatin + pemetrexed | Overall | 34 | 8.8 | 0 | -- | -- | | 2.9 | 20.6 | 8.8 | 11.8 |

**Notes: a)** Patient characteristics reported only for first-line and subsequent therapy combined. **Abbreviations:** ALK, anaplastic lymphoma kinase; EGFR, epithelial growth factor receptor; EHR, electronic health record; ICI, immune checkpoint inhibitor; KRAS, Kirsten rat sarcoma virus; L, line; PD-(L)1, programmed death (ligand)-1; Q3W, every 3 weeks; Q6W, every 6 weeks; ROS1, ROS proto-oncogene 1; TKI, tyrosine kinase inhibitor; TPS, tumor proportion score; WT, wild-type.

## Outcomes

Table S17: Summary of mOS in advanced/metastatic NSCLC studies

|  | **Pre-approval era** | | | **Post-approval era** | | | | | |
| --- | --- | --- | --- | --- | --- | --- | --- | --- | --- |
|  |  |  |  | **Monotherapy** | | | **Combination therapy** | | |
|  | **No. treatment groups** | **mOS in months, range** | **Median follow up in months, range** | **No. treatment groups** | **mOS in months, range** | **Median follow up in months, range** | **No. treatment groups** | **mOS in months, range** | **Median follow up in months, range** |
| **Tumor PD-L1 expression** | | | | | | | | | |
| TPS ≥50%^a,b^ | 18 | 6.9-18.4 | 9.7-35.4 | 36 | 10.64-NR | 6.93-28.6 | -- | | |
| TPS ≥1%^a,c^ |  |  |  | 9 | 14-NR | 3-23 |  |  |  |
| **Tumor histology** | | | | | | | | | |
| 100% NSQ^a^ | 11 | 6.9-18.4 | 22-34 | -- | | | 12 | 11.8-NR | 4.5-27 |
| 80-99% NSQ^a^ | 3 | 9-11.7 | 9.7-35.4 |  |  |  | 5 | 12.7-NR | 9.3-18 |
| 1-79% NSQ^a^ | 1 | 9.67 | Not reported |  |  |  | 5 | 16.6-NR | 9.7-17.13 |
| 100% SQ | 5 | 9-12.8 | Not reported |  |  |  | 2 | 10.6-15.3 | 5.7-26.2 |
| Unspecified^a^ | 2 | 9.3-12.3 | Not reported |  |  |  | 0 | -- | -- |

***Notes: a)*** *Treatment groups including ≥80% patients with no tumor EGFR/ALK aberrations.* ***b)*** *Treatment groups including ≥80% patients with PD-L1 CPS ≥50%.* ***c)*** *Treatment groups including ≥80% patients with PD-L1 CPS ≥1%.* ***Abbreviations:*** *ALK, anaplastic lymphoma kinase; EGFR, epidermal growth factor receptor; mOS, median overall survival; no., number; NR, not reached; NSQ, non-squamous; PD-L1, programmed death ligand-1; SQ, squamous; TPS, tumor proportion score.*

Table S18: Overall survival by treatment group in advanced/metastatic NSCLC studies

| **Study ID** | **Treatment** | **Overall/**  **subgroup** | **Median follow-up duration, months (range)** | **Median OS, months (95% CI)** | **Landmark OS** | | | | | | |
| --- | --- | --- | --- | --- | --- | --- | --- | --- | --- | --- | --- |
|  |  |  |  |  | **3-mo** | **6-mo** | **9-mo** | **12-mo** | **18-mo** | **2-y** | **3-y** |
| **Pre-approval era** | | | | | | | | | | | |
| Abernethy 2017 | Chemotherapy | Overall | -- | 294 (--)^b^ | -- | -- | -- | -- | -- | -- | -- |
| Banna 2017 | Doublet platinum-based chemotherapy | Overall | 9.7 (2-30) | 10.6 (9.8-11.6) | -- | -- | -- | -- | -- | -- | -- |
| Bonanno 2017 | Platinum-based chemotherapy ± bevacizumab | Overall | 35.4 (6-64.9)^a^ | 11.7 (9.1-15.3) | -- | -- | -- | -- | -- | -- | -- |
| Camerini 2016 | Doublet platinum-based chemotherapy + bevacizumab | Overall | -- | 10.5 (--) | -- | -- | -- | -- | -- | -- | -- |
| Chen 2014 | Icotinib | 1L | -- | 13 (10.31-15.70) | -- | -- | -- | -- | -- | -- | -- |
| Elsamany 2015 | Pemetrexed-containing chemotherapy | Overall | 22 (15-31) | 17.0 (12.87-21.13) | -- | -- | -- | -- | -- | -- | -- |
|  | Non-pemetrexed-containing chemotherapy | Overall |  | 11.0 (5.90-16.10) | -- | -- | -- | -- | -- | -- | -- |
|  | Chemotherapy or erlotinib | EGFR WT |  | 11.70 (8.24-15.16) | -- | -- | -- | -- | -- | -- | -- |
| Isobe 2017 | Chemotherapy | 1L, non-squamous, EGFR/ALK-negative or unknown | -- | 6.9 (5.6-10) | -- | -- | -- | -- | -- | -- | -- |
| Kohutek 2016 | Gemcitabine ± platinum chemotherapy | Overall | 34 (--) | 10.8 (6.72-14.52) | -- | -- | -- | -- | -- | -- | -- |
| Mudad 2017 | Nab-paclitaxel + carboplatin | Overall | -- | 12.8 (8.5-21.9) | -- | -- | -- | -- | -- | -- | -- |
|  | Gemcitabine + carboplatin | Overall | -- | 9 (6.9-11.5) | -- | -- | -- | -- | -- | -- | -- |
| Perez-Moreno 2016 | Pemetrexed + platinum | Overall | -- | 9 (4.08-13.92) | -- | -- | -- | -- | -- | -- | -- |
|  | Pemetrexed + platinum | No maintenance treatment | -- | 7 (5.09-8.91) | -- | -- | -- | -- | -- | -- | -- |
| de Castro 2017 | Chemotherapy | 1L, Italy | -- | 16.7 (12.6-26.2) | -- | -- | -- | -- | -- | -- | -- |
|  |  | 1L, Spain | -- | 10.7 (8.8-12.9) | -- | -- | -- | -- | -- | -- | -- |
|  |  | 1L, Germany | -- | -- (--) | -- | -- | -- | -- | -- | -- | -- |
|  |  | 1L, Australia | -- | 11.6 (9.2-14.6) | -- | -- | -- | -- | -- | -- | -- |
|  |  | 1L, Korea | -- | 12 (9.4-14.1) | -- | -- | -- | -- | -- | -- | -- |
|  |  | 1L, Taiwan | -- | 25.5 (22.6-31.3) | -- | -- | -- | -- | -- | -- | -- |
|  |  | 1L, Brazil | -- | 9.3 (7.6-11.5) | -- | -- | -- | -- | -- | -- | -- |
| Shen 2017 | Pemetrexed + cisplatin | Overall | -- | 18.4 (16.4-20.4) | -- | -- | -- | -- | -- | -- | -- |
|  | Pemetrexed + carboplatin | Overall | -- | 18.1 (17.1-19.1) | -- | -- | -- | -- | -- | -- | -- |
|  | Pemetrexed + cisplatin | No actionable mutations or gene status unknown | -- | 17.6 (15.4-19.8) | -- | -- | -- | -- | -- | -- | -- |
|  | Pemetrexed + carboplatin | No actionable mutations or gene status unknown | -- | 17 (16.3-17.7) | -- | -- | -- | -- | -- | -- | -- |
| Thippeswamy 2017 | Pemetrexed/platinum doublet | Non-squamous | -- | 11.2 (--) | -- | -- | -- | -- | -- | -- | -- |
|  | Taxane/platinum doublet | Non-squamous | -- | 9.4 (--) | -- | -- | -- | -- | -- | -- | -- |
|  | Nab-paclitaxel/platinum doublet | Non-squamous | -- | 10.2 (--) | -- | -- | -- | -- | -- | -- | -- |
|  | TKI/gefinib | Non-squamous | -- | 12.3 (--) | -- | -- | -- | -- | -- | -- | -- |
|  | Taxane/platinum doublet | Squamous | -- | 10.3 (--) | -- | -- | -- | -- | -- | -- | -- |
|  | Nab-paclitaxel/platinum doublet | Squamous | -- | 10.8 (--) | -- | -- | -- | -- | -- | -- | -- |
| von Verschuer 2017 | Chemotherapy combination or monotherapy or TKI | EGFR-negative | -- | 12.3 (10.6-14.3) | -- | -- | -- | 52 | -- | -- | -- |
| Xu 2017 | Icotinib | 1L | -- | 9.93 (0-23.49) | -- | -- | -- | -- | -- | -- | -- |
| **Post-approval era** | | | | | | | | | | | |
| Aggarwal 2023 | Pembrolizumab + pemetrexed + platinum | Overall | 6.09 (2.55-11.78)^d^ | 11.8 (10.82-12.76) | -- | -- | -- | -- | -- | -- | -- |
|  |  | Pembrolizumab ± pemetrexed maintenance therapy | 10.39 (6.84-16.18)^d^ | 21 (19.31-25.16) | -- | -- | -- | -- | -- | -- | -- |
|  |  | No maintenance therapy | 4.98 (3.11-9.39)^d^ | 9.1 (7.04-12.7) | -- | -- | -- | -- | -- | -- | -- |
| Akazawa 2022 | Atezolizumab or pembrolizumab | 1L, ≥90% PD-L1 | -- | 727 (509-843)^b^ | -- | -- | -- | -- | -- | -- | -- |
|  |  | 1L, <90% PD-L1 | -- | 508 (381-710)^b^ | -- | -- | -- | -- | -- | -- | -- |
| Attili 2022 | Pembrolizumab + pemetrexed + platinum | Overall | 12.4 (--)^a^ | 14.9 (11.3-NR) | -- | -- | -- | 58 | -- | -- | -- |
| Banna 2020 | Pembrolizumab | Overall | 16.3 (15-17.7) | -- | -- | -- | -- | -- | -- | 52 | -- |
| Bjornhart 2019 | Nivolumab or pembrolizumab | 1L, PD-L1 ≥50% | -- | NR (--) | -- | -- | -- | -- | -- | -- | -- |
| Bureau 2022 | Pembrolizumab | Overall | 26.9 (0.13-37.91) | 27.2 (21.7-NR) | -- | -- | -- | -- | -- | -- | -- |
| Cavaille 2020 | Pembrolizumab | Overall | 7.6 (--) | 11.08 (5.98-NR) | -- | 58.5 | -- | -- | -- | -- | -- |
| Chang 2022 | Pembrolizumab | 1L | -- | 25.6 (--) | -- | -- | -- | -- | -- | -- | -- |
| Chen 2021 | Pembrolizumab | Overall | 17.13 (--) | 28.91 (27.59-30.23) | -- | -- | -- | 76.1 | -- | -- | -- |
|  | Pembrolizumab + chemotherapy | Overall |  | NR (--) | -- | -- | -- | 89.3 | -- | -- | -- |
| Cortellini 2020 | Pembrolizumab | Overall | 14.6 (13.5-15.6)^a^ | 17.2 (15.3-22.3) | -- | -- | -- | -- | -- | -- | -- |
| Descourt 2023 | Pembrolizumab | Overall | 25.8 (24.8-26.7) ^a^ | 22.6 (18.5-27.4) | -- | 76.8 | -- | 64.8 | 54.3 | -- | -- |
| Di Noia 2021 | Pembrolizumab | Overall | 18.5 (--) | NR (--) | -- | 92.5 | -- | -- | -- | -- | -- |
| Dube-Pelletier 2023 | Pembrolizumab Q3W | Overall | 14.5 (--) | NR (--) | -- | -- | -- | -- | -- | -- | -- |
|  | Pembrolizumab Q6W | Overall | 18.3 (--) | 20.5 (13.7-29.8) | -- | -- | -- | -- | -- | -- | -- |
| Dudnik 2018 | Nivolumab monotherapy | 1L | -- | 4.6 (2.2-8.6) | -- | -- | -- | -- | -- | -- | -- |
| Dudnik 2021 | Pembrolizumab | Overall | 22.3 (14.5-28.9)^d^ | 12.5 (9.8-16.4) | -- | -- | -- | -- | -- | -- | -- |
|  | Pembrolizumab + chemotherapy | Overall | 9.1 (5.6-15.8)^d^ | 20.4 (10.8-NR) | -- | -- | -- | -- | -- | -- | -- |
| Eklund 2021 | Pembrolizumab | Overall | 7 (-----) | 13 (--) | -- | -- | -- | -- | -- | -- | -- |
| Faoro 2023 | Pembrolizumab | Overall | 13 (-----) | 13.6 (11.7-NR) | -- | -- | -- | 57.9 | -- | -- | -- |
| Frost 2021a | Pembrolizumab | Overall | 26.9 (23.8-29.9)^a^ | 22 (15.4-28.6) | -- | -- | -- | -- | -- | -- | -- |
| Frost 2021b | Pembrolizumab | Overall | 26.4 (24.3-28.5)^a^ | 23.6 (15-32.2) | -- | -- | -- | -- | -- | -- | -- |
| Fujimoto 2021 | Pembrolizumab + chemotherapy | Overall | 5.5 (3.8-7.1)^d^ | NR (--) | -- | -- | -- | -- | -- | -- | -- |
| Geiger-Gritsch 2021 | Nivolumab | 1L | 16.9 (0.2-28.2) | 17 (11.7-21.5) | -- | -- | -- | -- | -- | -- | -- |
| Genova 2023 | Pembrolizumab | 1L | 12.7 (0.27-43.4) | 15.5 (10.4-22.8) | -- | -- | -- | -- | -- | -- | -- |
| Goto 2022 | Pembrolizumab | Overall | 13.5 (<0.1-26.9) | NR (--) | -- | -- | -- | 72.2 | -- | 57.9 | -- |
| Hasegawa 2020 | Pembrolizumab | Overall | 11.3 (0.9-30.8) | 11.3 (5.2-NR) | -- | -- | -- | -- | -- | -- | -- |
| Holtzman 2022 | Pembrolizumab | Overall | 28.6 (14.6-38.6)^d^ | 13.8 (11-18.2) | -- | -- | -- | -- | -- | -- | -- |
|  | Pembrolizumab + platinum-based chemotherapy | Overall | 15.5 (9.3-19.1)^d^ | 21.3 (14.8-NR) | -- | -- | -- | -- | -- | -- | -- |
| Hu 2022 | Anti-PD-(L)1 monotherapy or combination therapy | 1L, PD-L1 positive | 10.9 (--) | NR (--) | -- | -- | -- | -- | -- | -- | -- |
|  |  | 1L, PD-L1 negative |  | 13.2 (--) | -- | -- | -- | -- | -- | -- | -- |
| Ikeuchi 2023 | Atezolizumab + bevacizumab + carboplatin + paclitaxel | EGFR-WT | 10.3 (--) | NR (10.3-NR) | -- | -- | -- | 66.3 | -- | 59.7 | -- |
| Imai 2021 | Pembrolizumab | Overall | 15.7 (0.1-39.6) | 17.4 (12.4-31.3) | -- | -- | -- | -- | -- | -- | -- |
| Isono 2021 | Pembrolizumab + chemotherapy | Overall | 9.7 (1.8-18.9) | 16.6 (10.1-NR) | -- | -- | -- | -- | -- | -- | -- |
|  | Pembrolizumab | Overall | 12 (0.4-40.3) | 27 (15.9-NR) | -- | -- | -- | -- | -- | -- | -- |
| Ivanovic 2021 | Pembrolizumab | 1L | 19.9 (--) | NR (7.1-NR) | -- | -- | -- | 62 | -- | -- | -- |
| Kaira 2023 | Pembrolizumab | Overall | 364 (--)^b^ | 521 (--)^b^ | -- | -- | -- | -- | -- | -- | -- |
| Lang 2019 | Nivolumab, pembrolizumab, or atezolizumab monotherapy | 1L | -- | 17 (9-NR) | -- | -- | -- | -- | -- | -- | -- |
| Lenci 2021 | Pembrolizumab | Overall | 24 (16.6-32.6)^a^ | 15.6 (12-NR) | -- | -- | -- | -- | -- | -- | -- |
| Lester 2021 | Pembrolizumab, nivolumab, or atezolizumab | Overall | 12.7 (0.1-37.3) | 14 (10.7-20.6) | -- | -- | -- | -- | -- | -- | -- |
| Li 2022 | Pembrolizumab + carboplatin + paclitaxel/pemetrexed | Overall | -- | 17.6 (14.4-20.8) | -- | 92.5 | -- | 72.5 | -- | 43.8 | -- |
| Liao 2021 | Pembrolizumab + pemetrexed + cisplatin/carboplatin | Overall | 8 (5.61-12.06)^d^ | NR (--) | -- | -- | -- | -- | -- | -- | -- |
| Liu 2022 | Pembrolizumab + pemetrexed + carboplatin | Overall | 18.9 (1 day-39.5) | 17.2 (13.6-19.9) | -- | -- | -- | 59.4 | -- | 39.2 | -- |
| Liu 2023 | Pembrolizumab + carboplatin + paclitaxel/nab-paclitaxel | Overall | 26.2 (17.1-36) | 15.3 (11.7-18.6) | -- | -- | -- | 54.9 | -- | 37.3 | -- |
| Low 2021 | Pembrolizumab 200mg | Overall | 14.8 (--) | 19.8 (--) | -- | -- | -- | -- | -- | -- | -- |
|  | Pembrolizumab 100mg | Overall | 14.8 (--) | 14.3 (--) | -- | -- | -- | -- | -- | -- | -- |
|  | Pembrolizumab <2 mg/kg | Overall | 14.8 ---) | 14.7 (--) | -- | -- | -- | -- | -- | -- | -- |
|  | Pembrolizumab ≥2 mg/kg | Overall | 14.8 ---) | 13.5 (--) | -- | -- | -- | -- | -- | -- | -- |
|  | Pembrolizumab 200mg + chemotherapy | Overall | 14.8 (--) | NR (--) | -- | -- | 58 | -- | -- | -- | -- |
|  | Pembrolizumab 100mg + chemotherapy | Overall | 14.8 (--) | NR (--) | -- | -- | 85 | -- | -- | -- | -- |
| Matusomoto 2022 | Pembrolizumab monotherapy | Overall | 379 (58-1169)^b^ | NR (889-NR)^b^ | -- | -- | -- | -- | -- | -- | -- |
|  | Pembrolizumab combination therapy | Overall | 271 (44-552)^b^ | NR (NR-NR)^b^ | -- | -- | -- | -- | -- | -- | -- |
| Nindra 2023 | Durvalumab | EGFR WT | 15.1 (-----) | NR (--) | -- | -- | -- | -- | -- | -- | -- |
| Nokihara 2022 | Pembrolizumab, nivolumab, or atezolizumab | Overall | 11.3 (<0.1-26.9) | NR (--) | -- | 83.5 | -- | 72.1 | -- | 57.8 | -- |
| Noordhof 2021 | Pembrolizumab | Overall | 19.1 (-----) | 17.2 (--) | 79 | -- | -- | 57 | -- | 44 | -- |
| Amrane 2020 | Pembrolizumab | Overall | 8.2 (0.9-20.9) | 15.2 (13.9-NR) | -- | 86.2 | -- | -- | -- | -- | -- |
| Perol 2022 | PD-(L)1 inhibitor monotherapy | Overall | 23.5 (15.7-28.7)^d^ | 22.1 (18.3-30.3) | -- | -- | -- | -- | -- | -- | -- |
|  | PD-(L)1 inhibitor + platinum-doublet chemotherapy | Overall | 19.9 (14.9-26.3)^d^ | 21 (15.3-NR) | -- | -- | -- | -- | -- | -- | -- |
| Raez 2022 | Durvalumab | Overall | 19.6 (8.1-39.2)^a^ | 26.3 (23.9-28.6) | -- | -- | -- | -- | -- | -- | -- |
| Renaud 2023 | Pembrolizumab + pemetrexed + carboplatin | Overall | 4.5 (0-22) | 20.6 (17-NR) | -- | -- | -- | -- | -- | -- | -- |
| Sanchez-Gastaldo 2021 | Pembrolizumab | Overall | 6.93 (0.2-26.19) | 10.64 (4.5-17.05) | -- | -- | -- | -- | -- | -- | -- |
| Seban 2020 | Pembrolizumab | Overall | 13.4 (9-17.9)^a^ | 12.1 (8.6-15.6) | -- | -- | -- | -- | -- | -- | -- |
| Seban 2021 | Pembrolizumab | Overall | 26.5 (18.8-36.2)^a^ | NR (21.8-NR) | -- | -- | -- | -- | -- | 61 | -- |
| Shah 2022a | PD-(L)1 inhibitor monotherapy | Very high PD-L1 expression | 1.38 (0.6-2.37)^d,e^ | 3.85 (0.86-NR)^d,e^ | -- | -- | -- | -- | -- | -- | -- |
|  |  | High PD-L1 expression | 1.03 (0.32-2.32)^d,e^ | 1.49 (0.31-3.96)^d,e^ | -- | -- | -- | -- | -- | -- | -- |
| Shah 2022b | Pembrolizumab | Very high PD-L1 expression, IPW | 9.33 (2.74-22.3)^d^ | 15.84 (3.7-NR)^d^ | -- | -- | -- | 55 | -- | 42 | 35 |
|  |  | High PD-L1 expression, IPW |  | 12.72 (3.24-33.24)^d^ | -- | -- | -- | 52 | -- | 34 | 23 |
| Shah 2023 | Pembrolizumab + carboplatin-based chemotherapy | Overall | 9.3 (2.9-21.6)^d^ | 16.6 (--) | -- | 74 | -- | -- | -- | -- | -- |
| Shah 2023 | Pembrolizumab | Overall |  | 15.2 (--) | -- | 68 | -- | -- | -- | -- | -- |
| Shalata 2023 | Pembrolizumab (combination or monotherapy) | Overall | 36 (28-65) | 36 (23-55)^c^ | -- | -- | -- | -- | -- | -- | -- |
| Anpalakhan 2023a, Anpalakhan 2023b, Banna 2022 | Pembrolizumab + chemotherapy | Overall | 18 (15.9-20.1)^a^ | 12.7 (10.2-15.2) | -- | -- | -- | 52.5 | -- | 27.5 | -- |
| Tamayo-Bermejo 2023 | Pembrolizumab | 1L | 3 (1-38) | 19 (13.36-24.63) | -- | -- | -- | -- | -- | -- | -- |
| Tamiya 2019 | Pembrolizumab | Overall | 11 (--) | 17.8 (17.8-NR) | -- | -- | -- | -- | -- | -- | -- |
| Tang 2022 | ICI ± chemotherapy | 1L | -- | 10.2 (--) | -- | -- | -- | 44.6 | -- | -- | -- |
|  | ICI monotherapy | 1L | -- | 10.4 (--) | -- | -- | -- | 47.8 | -- | -- | -- |
|  | ICI + chemotherapy | 1L | -- | 9.8 (--) | -- | -- | -- | 43.2 | -- | -- | -- |
| Tibaldi 2022 | Pembrolizumab | Overall | 15.2 (--) | 15.9 (NR-NR) | -- | -- | -- | -- | -- | -- | -- |
| Tsai 2022 | Pembrolizumab | Overall | -- | 17.4 (2.7-NR)^d^ | -- | -- | -- | -- | -- | -- | -- |
| Veccia 2021 | Pembrolizumab | Overall | 11.7 (0.4-36.1) | 17.4 (12-24.4) | -- | -- | -- | -- | -- | -- | -- |
| Velcheti 2019 | Pembrolizumab | EHR cohort | 11.3 (0.1-28.7) | 18.9 (14.9-25.5) | -- | 74 | -- | 59.1 | -- | -- | -- |
|  |  | Spotlight cohort | 11.8 (0.1-22.1) | 19.1 (12.6-NR) | -- | 74.3 | -- | 60.4 | -- | -- | -- |
| Velcheti 2021 | Pembrolizumab + carboplatin + pemetrexed | Overall | 20.3 (12-28) | 16.5 (13.2-20.6) | -- | 75.4 | -- | 59.5 | 48.6 | -- | -- |
| Velcheti 2022 | Pembrolizumab | EHR cohort | 16.5 (<0.1-52.6) | 19.6 (16.6-24.3) | -- | -- | -- | 59.8 | -- | 45.7 | 36.2 |
|  |  | Spotlight cohort | 25.7 (1 day-44.3) | 21.1 (16.2-28.9) | -- | -- | -- | 64.2 | -- | 49.4 | 38.3 |
| Wang 2021 | Anti-PD-(L)1 monotherapy | 1L | -- | 17 (11.4-22.6) | -- | -- | -- | -- | -- | -- | -- |
| Waterhouse 2021 | Atezolizumab or pembrolizumab combination therapy | Squamous | 5.7 (--) | 10.6 (9.3-11.8) | -- | -- | -- | 45.1 | -- | -- | -- |
|  |  | Non-squamous | 6.3 (--) | 12 (11.3-12.8) | -- | -- | -- | 49.9 | -- | 32.5 | -- |
|  | Atezolizumab or pembrolizumab monotherapy | Squamous | 6.6 (--) | 11.3 (9.8-12.8) | -- | -- | -- | 48.7 | -- | 27.8 | 18.2 |
|  |  | Non-squamous | 7.2 (--) | 14.1 (12.4-15.8) | -- | -- | -- | 52.9 | -- | 38.5 | 29.9 |
| Xu 2022 | Atezolizumab + carboplatin + nab-paclitaxel | Overall | 27 (1-37) | 19.9 (16.3-22.5) | 98.3 | -- | -- | 91.4 | -- | 42.2 | -- |
| Yoneda 2022 | Nivolumab, pembrolizumab, or atezolizumab monotherapy | 1L | -- | 18.5 (11.1-27.5) | -- | -- | -- | -- | -- | -- | -- |
|  |  | 1L, PD-L1 TPS ≥50% | -- | 19.1 (--) | -- | -- | -- | -- | -- | -- | -- |
| Yuasa 2023 | ICI ± chemotherapy | 1L | 14.6 (4.5-30.1)^d^ | 25 (12.7-41.2) | -- | -- | -- | -- | -- | -- | -- |
| Zayas-Soriano 2020 | Pembrolizumab monotherapy | 1L | 23 (--) | -- | -- | -- | -- | -- | -- | 100 | -- |
| Zhang 2021 | Pembrolizumab + cisplatin + pemetrexed | Overall | 24.2 (12.8-36.6) | 23.1 (16.6-32.8) | -- | -- | -- | -- | -- | -- | -- |

**Notes: a)** Median (95% CI). **b)** Reported in days. **c)** Median (range). **d)** Median (interquartile range). **e)** Reported in years. **Abbreviations:** ALK, anaplastic lymphoma kinase; CI, confidence interval; EGFR, epidermal growth factor receptor; HER, electronic health record; ICI, immune checkpoint inhibitor; IPW, inverse probability-weighted; L, line, mo., month; NR, not reached; OS, overall survival; PD-(L)1, programmed death (ligand)-1; Q3W, every 3 weeks; Q6W, every 6 weeks; TKI, tyrosine kinase inhibitor; TPS, tumor proportion score; WT, wild-type; y, year.

## Quality Assessment

Table S19: Quality assessment of advanced/metastatic NSCLC studies

| **Study ID** | **Selection** | | | | **Comparability** | **Outcome** | | | **Total score** |
| --- | --- | --- | --- | --- | --- | --- | --- | --- | --- |
|  | **1** | **2** | **3** | **4** | **1** | **1** | **2** | **3** |  |
| **Pre-approval era** | | | | | | | | | |
| Abernethy 2017 | 1 | NA | 1 | 1 | NA | 1 | 1 | 1 | 6 |
| Banna 2017 | 0 | NA | 0 | 1 | NA | 0 | 1 | 1 | 3 |
| Bonanno 2017 | 1 | NA | 1 | 1 | NA | 1 | 1 | 1 | 6 |
| Camerini 2016 | 1 | NA | 1 | 1 | NA | 1 | 1 | 1 | 6 |
| Chen 2014 | 1 | NA | 1 | 1 | NA | 1 | 1 | 1 | 6 |
| Elsamany 2015 | 1 | NA | 1 | 1 | NA | 1 | 1 | 1 | 6 |
| Isobe 2017 | 1 | NA | 1 | 1 | NA | 1 | 1 | 1 | 6 |
| Kohutek 2016 | 0 | NA | 0 | 1 | NA | 0 | 1 | 1 | 3 |
| Mudad 2017 | 1 | NA | 1 | 1 | NA | 1 | 1 | 1 | 6 |
| Perez-Moreno 2016 | 1 | NA | 1 | 1 | NA | 1 | 1 | 1 | 6 |
| de Castro 2017 | 1 | NA | 1 | 1 | NA | 1 | 1 | 1 | 6 |
| Shen 2017 | 0 | NA | 1 | 1 | NA | 1 | 1 | 1 | 5 |
| Thippeswamy 2017 | 0 | NA | 1 | 1 | NA | 1 | 1 | 1 | 5 |
| von Verschuer 2017 | 1 | NA | 1 | 1 | NA | 1 | 1 | 1 | 6 |
| Xu 2017 | 0 | NA | 1 | 1 | NA | 1 | 1 | 1 | 5 |
| **Post-approval era** | | | | | | | | | |
| Aggarwal 2023 | 1 | NA | 1 | 1 | NA | 1 | 1 | 1 | 6 |
| Akazawa 2022 | 0 | NA | 1 | 1 | NA | 1 | 1 | 1 | 5 |
| Attili 2022 | 0 | NA | 1 | 1 | NA | 1 | 1 | 1 | 5 |
| Banna 2020 | 1 | NA | 1 | 1 | NA | 1 | 1 | 1 | 6 |
| Bjornhart 2019 | 0 | NA | 1 | 1 | NA | 1 | 0 | 1 | 4 |
| Bureau 2022 | 1 | NA | 1 | 1 | NA | 1 | 1 | 1 | 6 |
| Cavaille 2020 | 0 | NA | 1 | 1 | NA | 1 | 1 | 1 | 5 |
| Chang 2022 | 1 | NA | 1 | 1 | NA | 1 | 1 | 1 | 6 |
| Chen 2021 | 0 | NA | 1 | 1 | NA | 1 | 1 | 1 | 5 |
| Cortellini 2020 | 1 | NA | 1 | 1 | NA | 1 | 1 | 1 | 6 |
| Descourt 2023 | 1 | NA | 1 | 1 | NA | 1 | 1 | 1 | 6 |
| Di Noia 2021 | 0 | NA | 1 | 1 | NA | 1 | 1 | 1 | 5 |
| Dube-Pelletier 2023 | 0 | NA | 1 | 1 | NA | 1 | 1 | 1 | 5 |
| Dudnik 2018 | 1 | NA | 1 | 1 | NA | 1 | 1 | 1 | 6 |
| Dudnik 2021 | 1 | NA | 1 | 1 | NA | 1 | 1 | 1 | 6 |
| Eklund 2021 | 1 | NA | 1 | 1 | NA | 1 | 1 | 1 | 6 |
| Faoro 2023 | 0 | NA | 1 | 1 | NA | 1 | 1 | 1 | 5 |
| Frost 2021a | 1 | NA | 1 | 1 | NA | 1 | 1 | 1 | 6 |
| Frost 2021b | 1 | NA | 1 | 1 | NA | 1 | 1 | 1 | 6 |
| Fujimoto 2021 | 1 | NA | 1 | 1 | NA | 1 | 0 | 1 | 5 |
| Geiger-Gritsch 2021 | 1 | NA | 1 | 1 | NA | 1 | 1 | 1 | 6 |
| Genova 2023 | 0 | NA | 1 | 0 | NA | 1 | 1 | 1 | 4 |
| Goto 2022 | 1 | NA | 1 | 1 | NA | 1 | 1 | 1 | 6 |
| Hasegawa 2020 | 0 | NA | 1 | 1 | NA | 1 | 1 | 1 | 5 |
| Holtzman 2022 | 1 | NA | 1 | 1 | NA | 1 | 1 | 1 | 6 |
| Hu 2022 | 0 | NA | 1 | 1 | NA | 1 | 1 | 1 | 5 |
| Ikeuchi 2023 | 0 | NA | 1 | 1 | NA | 1 | 1 | 1 | 5 |
| Imai 2021 | 1 | NA | 1 | 1 | NA | 1 | 1 | 1 | 6 |
| Isono 2021 | 0 | NA | 1 | 1 | NA | 1 | 1 | 1 | 5 |
| Kaira 2023 | 0 | NA | 1 | 1 | NA | 1 | 1 | 1 | 5 |
| Lang 2019 | 1 | NA | 1 | 1 | NA | 1 | 1 | 1 | 6 |
| Lenci 2021 | 1 | NA | 1 | 1 | NA | 1 | 1 | 1 | 6 |
| Lester 2021 | 1 | NA | 1 | 1 | NA | 1 | 1 | 1 | 6 |
| Li 2022 | 0 | NA | 1 | 1 | NA | 1 | 1 | 1 | 5 |
| Liao 2021 | 0 | NA | 1 | 1 | NA | 1 | 0 | 1 | 4 |
| Liu 2022 | 1 | NA | 1 | 1 | Yes | 1 | 1 | 1 | 6 |
| Liu 2023 | 1 | NA | 1 | 1 | Yes | 1 | 1 | 1 | 6 |
| Low 2021 | 0 | NA | 1 | 1 | NA | 1 | 1 | 1 | 5 |
| Matusomoto 2022 | 1 | NA | 1 | 1 | NA | 1 | 1 | 1 | 6 |
| Nindra 2023 | 1 | NA | 1 | 1 | NA | 1 | 1 | 1 | 6 |
| Nokihara 2022 | 1 | NA | 1 | 1 | NA | 1 | 1 | 1 | 6 |
| Noordhof 2021 | 1 | NA | 1 | 1 | NA | 1 | 1 | 1 | 6 |
| Amrane 2020 | 1 | NA | 1 | 1 | NA | 1 | 1 | 1 | 6 |
| Perol 2022 | 1 | NA | 1 | 1 | NA | 1 | 1 | 1 | 6 |
| Raez 2022 | 1 | NA | 0 | 1 | NA | 0 | 1 | 1 | 4 |
| Renaud 2023 | 1 | NA | 1 | 1 | NA | 1 | 1 | 1 | 6 |
| Sanchez-Gastaldo 2021 | 0 | NA | 1 | 1 | NA | 1 | 1 | 1 | 5 |
| Seban 2020 | 1 | NA | 1 | 1 | NA | 1 | 1 | 1 | 6 |
| Seban 2021 | 1 | NA | 1 | 1 | NA | 1 | 1 | 1 | 6 |
| Shah 2022a | 0 | NA | 1 | 1 | NA | 1 | 1 | 1 | 5 |
| Shah 2022b | 1 | NA | 1 | 1 | NA | 1 | 1 | 1 | 6 |
| Shah 2023 | 1 | NA | 1 | 1 | NA | 1 | 1 | 1 | 6 |
| Shalata 2023 | 0 | NA | 1 | 1 | NA | 1 | 1 | 1 | 5 |
| Anpalakhan 2023a, Anpalakhan 2023b, Banna 2022 | 1 | NA | 1 | 1 | NA | 1 | 1 | 1 | 6 |
| Tamayo-Bermejo 2023 | 0 | NA | 1 | 1 | NA | 1 | 1 | 1 | 5 |
| Tamiya 2019 | 1 | NA | 1 | 1 | NA | 1 | 1 | 1 | 6 |
| Tang 2022 | 1 | NA | 1 | 1 | NA | 1 | 1 | 1 | 6 |
| Tibaldi 2022 | 1 | NA | 1 | 1 | NA | 1 | 1 | 1 | 6 |
| Tsai 2022 | 0 | NA | 1 | 1 | NA | 1 | 1 | 1 | 5 |
| Veccia 2021 | 1 | NA | 1 | 1 | 1 | 1 | 1 | 1 | 7 |
| Velcheti 2019 | 1 | NA | 1 | 1 | 1 | 1 | 1 | 1 | 7 |
| Velcheti 2021 | 1 | NA | 1 | 1 | 1 | 1 | 1 | 1 | 7 |
| Velcheti 2022 | 1 | NA | 1 | 1 | 1 | 1 | 1 | 1 | 7 |
| Wang 2021 | 0 | NA | 1 | 1 | NA | 1 | 1 | 1 | 5 |
| Waterhouse 2021 | 1 | NA | 1 | 1 | NA | 1 | 1 | 1 | 6 |
| Xu 2022 | 1 | NA | 1 | 1 | NA | 1 | 1 | 1 | 6 |
| Yoneda 2022 | 1 | NA | 1 | 1 | NA | 1 | 1 | 1 | 6 |
| Yuasa 2023 | 0 | NA | 1 | 1 | NA | 1 | 1 | 1 | 5 |
| Zayas-Soriano 2020 | 0 | NA | 1 | 1 | NA | 1 | 1 | 1 | 5 |
| Zhang 2021 | 0 | NA | 1 | 1 | NA | 1 | 1 | 1 | 5 |

*Abbreviations: NA, not applicable.*

# Advanced RCC

## Literature Search Strategies

Table S20: Advanced RCC, pre-approval era: Embase search strategy

*Database: Embase <1974 to 2023 July 7>; Search date: July 10, 2023*

| **Line** | **Search terms** | **Hits** |
| --- | --- | --- |
| 1 | exp carcinoma, renal cell/ or exp renal cell carcinoma/ | 36721 |
| 2 | (((renal or kidney or nephroid or hypernephroid or collecting-duct) adj3 (carcinoma* or cancer* or malignan* or tumo?r* or neoplas* or adenocarcinoma* or pyelocarcinoma*)) or RCC or hypernephroma* or grawitz tumo?r* or nephrocarcinoma*).mp. | 176292 |
| 3 | or/1-2 | 179879 |
| 4 | exp metastasis/ or exp neoplasm metastasis/ or (advance$ or metasta$ or recurr$ or unresect$ or non-resect$ or disseminated or stage 3 or stage III* or stage 4 or stage IV* or spread$ or migration$ or progress$ or invasive or aggressive or "not operable" or untreatable or "not treatable" or secondary or incurable or "not curable").mp. | 7409869 |
| 5 | 3 and 4 | 99423 |
| 6 | exp overall survival/ or exp survival rate/ or exp life expectancy/ or exp survival analysis/ or (surviv* or OS or mortality or death* or die* or life expectancy).mp. | 6478513 |
| 7 | Clinical study/ | 163725 |
| 8 | Case control study/ | 207756 |
| 9 | Family study/ | 25786 |
| 10 | Longitudinal study/ | 196031 |
| 11 | Retrospective study/ | 1490609 |
| 12 | Prospective study/ | 886615 |
| 13 | Randomized controlled trials/ | 264163 |
| 14 | 12 not 13 | 875476 |
| 15 | Prospective study/ | 886615 |
| 16 | Cohort analysis/ | 1056494 |
| 17 | (Cohort adj (study or studies)).mp. | 485180 |
| 18 | (Case control adj (study or studies)).tw. | 170347 |
| 19 | (follow up adj (study or studies)).tw. | 74216 |
| 20 | (observational adj (study or studies)).tw. | 257702 |
| 21 | (epidemiologic$ adj (study or studies)).tw. | 123233 |
| 22 | (cross sectional adj (study or studies)).tw. | 343578 |
| 23 | or/7-11, 14-22 | 3974056 |
| 24 | case study/ | 99618 |
| 25 | case report/ or case report.tw. | 2963471 |
| 26 | conference abstract/ or conference paper/ or conference review/ | 2711315 |
| 27 | editorial/ | 751065 |
| 28 | letter/ | 1234492 |
| 29 | note/ | 886897 |
| 30 | review/ or short survey/ | 3514301 |
| 31 | or/24-30 | 11437910 |
| 32 | 23 not 31 | 3058593 |
| 33 | 5 and 6 and 32 | 8501 |
| 34 | limit 33 to yr=2010 - 2019 | 4545 |
| 35 | limit 34 to english | 4369 |

Table S21: Advanced RCC, pre-approval era: MEDLINE search strategy

*Database: Ovid MEDLINE(R) and Epub Ahead of Print, In-Process, In-Data-Review & Other Non-Indexed Citations and Daily 1946 to July 06, 2023; Search date: July 10, 2023*

| **Line** | **Search terms** | **Hits** |
| --- | --- | --- |
| 1 | exp carcinoma, renal cell/ or exp renal cell carcinoma/ | 40190 |
| 2 | (((renal or kidney or nephroid or hypernephroid or collecting-duct) adj3 (carcinoma* or cancer* or malignan* or tumo?r* or neoplas* or adenocarcinoma* or pyelocarcinoma*)) or RCC or hypernephroma* or grawitz tumo?r* or nephrocarcinoma*).mp. | 115424 |
| 3 | or/1-2 | 115424 |
| 4 | exp metastasis/ or exp neoplasm metastasis/ or (advance$ or metasta$ or recurr$ or unresect$ or non-resect$ or disseminated or stage 3 or stage III* or stage 4 or stage IV* or spread$ or migration$ or progress$ or invasive or aggressive or "not operable" or untreatable or "not treatable" or secondary or incurable or "not curable").mp. | 5285610 |
| 5 | 3 and 4 | 56103 |
| 6 | exp overall survival/ or exp survival rate/ or exp life expectancy/ or exp survival analysis/ or (surviv* or OS or mortality or death* or die* or life expectancy).mp. | 4656980 |
| 7 | Epidemiologic studies/ | 9353 |
| 8 | Exp case control studies/ | 1428358 |
| 9 | Exp cohort studies/ | 2498788 |
| 10 | Case control.tw. | 154046 |
| 11 | (cohort adj (study or studies)).tw. | 316754 |
| 12 | Cohort analy$.tw. | 11810 |
| 13 | (Follow up adj (study or studies)).tw. | 56299 |
| 14 | (observational adj (study or studies)).tw. | 161394 |
| 15 | Longitudinal.tw. | 322757 |
| 16 | Retrospective.tw. | 745567 |
| 17 | Cross sectional.tw. | 513409 |
| 18 | Cross-sectional studies/ | 471331 |
| 19 | or/7-18 | 3796175 |
| 20 | case reports/ or case report.tw. | 2404681 |
| 21 | editorial/ | 656410 |
| 22 | historical article/ | 369352 |
| 23 | letter/ | 1222167 |
| 24 | review/ or systematic review/ or meta-analysis/ | 3343956 |
| 25 | or/20-24 | 7556164 |
| 26 | 19 not 25 | 3427154 |
| 27 | 5 and 6 and 26 | 7068 |
| 28 | limit 27 to yr=2010 - 2019 | 3327 |
| 29 | limit 28 to english | 3148 |

Table S22: Advanced RCC, post-approval era: Embase search strategy

*Database: Embase <1974 to 2023 July 7>; Search date: July 10, 2023*

| **Line** | **Search terms** | **Hits** |
| --- | --- | --- |
| 1 | exp carcinoma, renal cell/ or exp renal cell carcinoma/ | 36721 |
| 2 | (((renal or kidney or nephroid or hypernephroid or collecting-duct) adj3 (carcinoma* or cancer* or malignan* or tumo?r* or neoplas* or adenocarcinoma* or pyelocarcinoma*)) or RCC or hypernephroma* or grawitz tumo?r* or nephrocarcinoma*).mp. | 176292 |
| 3 | or/1-2 | 179879 |
| 4 | exp metastasis/ or exp neoplasm metastasis/ or (advance$ or metasta$ or recurr$ or unresect$ or non-resect$ or disseminated or stage 3 or stage III* or stage 4 or stage IV* or spread$ or migration$ or progress$ or invasive or aggressive or "not operable" or untreatable or "not treatable" or secondary or incurable or "not curable").mp. | 7409869 |
| 5 | 3 and 4 | 99423 |
| 6 | exp avelumab/ or (avelumab or MSB0010718C or bavencio).mp. | 6320 |
| 7 | exp nivolumab/ or (nivolumab or opdivo or ONO-4538 or BMS-936558 or MDX1106).mp. | 38765 |
| 8 | exp pembrolizumab/ or (pembrolizumab or MK-3475 or SCH-900475 or lambrolizumab or keytruda).mp. | 37947 |
| 9 | exp immune checkpoint inhibitors/ or (checkpoint or ICI* or (PD1 or PD-1 or PDL1 or PD-L1) and (inhibit*)).mp. | 141080 |
| 10 | or/6-9 | 171663 |
| 11 | exp overall survival/ or exp survival rate/ or exp life expectancy/ or exp survival analysis/ or (surviv* or OS or mortality or death* or die* or life expectancy).mp. | 6478513 |
| 12 | Clinical study/ | 163725 |
| 13 | Case control study/ | 207756 |
| 14 | Family study/ | 25786 |
| 15 | Longitudinal study/ | 196031 |
| 16 | Retrospective study/ | 1490609 |
| 17 | Prospective study/ | 886615 |
| 18 | Randomized controlled trials/ | 264163 |
| 19 | 17 not 18 | 875476 |
| 20 | Prospective study/ | 886615 |
| 21 | Cohort analysis/ | 1056494 |
| 22 | (Cohort adj (study or studies)).mp. | 485180 |
| 23 | (Case control adj (study or studies)).tw. | 170347 |
| 24 | (follow up adj (study or studies)).tw. | 74216 |
| 25 | (observational adj (study or studies)).tw. | 257702 |
| 26 | (epidemiologic$ adj (study or studies)).tw. | 123233 |
| 27 | (cross sectional adj (study or studies)).tw. | 343578 |
| 28 | or/12-16, 19-27 | 3974056 |
| 29 | case study/ | 99618 |
| 30 | case report/ or case report.tw. | 2963471 |
| 31 | conference abstract/ or conference paper/ or conference review/ | 2711315 |
| 32 | editorial/ | 751065 |
| 33 | letter/ | 1234492 |
| 34 | note/ | 886897 |
| 35 | review/ or short survey/ | 3514301 |
| 36 | or/29-35 | 11437910 |
| 37 | 28 not 36 | 3058593 |
| 38 | 5 and 10 and 11 and 37 | 1078 |
| 39 | limit 38 to yr=2015 - current | 1063 |
| 40 | limit 39 to english | 1055 |

Table S23: Advanced RCC, post-approval era: MEDLINE search strategy

*Database: Embase <1974 to 2023 July 7>; Search date: July 10, 2023*

| **Line** | **Search terms** | **Hits** |
| --- | --- | --- |
| 1 | exp carcinoma, renal cell/ or exp renal cell carcinoma/ | 40190 |
| 2 | (((renal or kidney or nephroid or hypernephroid or collecting-duct) adj3 (carcinoma* or cancer* or malignan* or tumo?r* or neoplas* or adenocarcinoma* or pyelocarcinoma*)) or RCC or hypernephroma* or grawitz tumo?r* or nephrocarcinoma*).mp. | 115424 |
| 3 | or/1-2 | 115424 |
| 4 | exp metastasis/ or exp neoplasm metastasis/ or (advance$ or metasta$ or recurr$ or unresect$ or non-resect$ or disseminated or stage 3 or stage III* or stage 4 or stage IV* or spread$ or migration$ or progress$ or invasive or aggressive or "not operable" or untreatable or "not treatable" or secondary or incurable or "not curable").mp. | 5285610 |
| 5 | 3 and 4 | 56103 |
| 6 | exp avelumab/ or (avelumab or MSB0010718C or bavencio).mp. | 932 |
| 7 | exp nivolumab/ or (nivolumab or opdivo or ONO-4538 or BMS-936558 or MDX1106).mp. | 9609 |
| 8 | exp pembrolizumab/ or (pembrolizumab or MK-3475 or SCH-900475 or lambrolizumab or keytruda).mp. | 8916 |
| 9 | exp immune checkpoint inhibitors/ or (checkpoint or ICI* or (PD1 or PD-1 or PDL1 or PD-L1) and (inhibit*)).mp. | 68254 |
| 10 | or/6-9 | 72791 |
| 11 | exp overall survival/ or exp survival rate/ or exp life expectancy/ or exp survival analysis/ or (surviv* or OS or mortality or death* or die* or life expectancy).mp. | 4656980 |
| 12 | Epidemiologic studies/ | 9353 |
| 13 | Exp case control studies/ | 1428358 |
| 14 | Exp cohort studies/ | 2498788 |
| 15 | Case control.tw. | 154046 |
| 16 | (cohort adj (study or studies)).tw. | 316754 |
| 17 | Cohort analy$.tw. | 11810 |
| 18 | (Follow up adj (study or studies)).tw. | 56299 |
| 19 | (observational adj (study or studies)).tw. | 161394 |
| 20 | Longitudinal.tw. | 322757 |
| 21 | Retrospective.tw. | 745567 |
| 22 | Cross sectional.tw. | 513409 |
| 23 | Cross-sectional studies/ | 471331 |
| 24 | or/12-23 | 3796175 |
| 25 | case reports/ or case report.tw. | 2404681 |
| 26 | editorial/ | 656410 |
| 27 | historical article/ | 369352 |
| 28 | letter/ | 1222167 |
| 29 | review/ or systematic review/ or meta-analysis/ | 3343956 |
| 30 | or/25-29 | 7556164 |
| 31 | 24 not 30 | 3427154 |
| 32 | 5 and 10 and 11 and 31 | 456 |
| 33 | limit 32 to yr=2015 - current | 454 |
| 34 | limit 33 to english | 450 |

## Study Selection

**Figure S5: PRISMA flow diagram: advanced RCC studies in the pre-approval era**


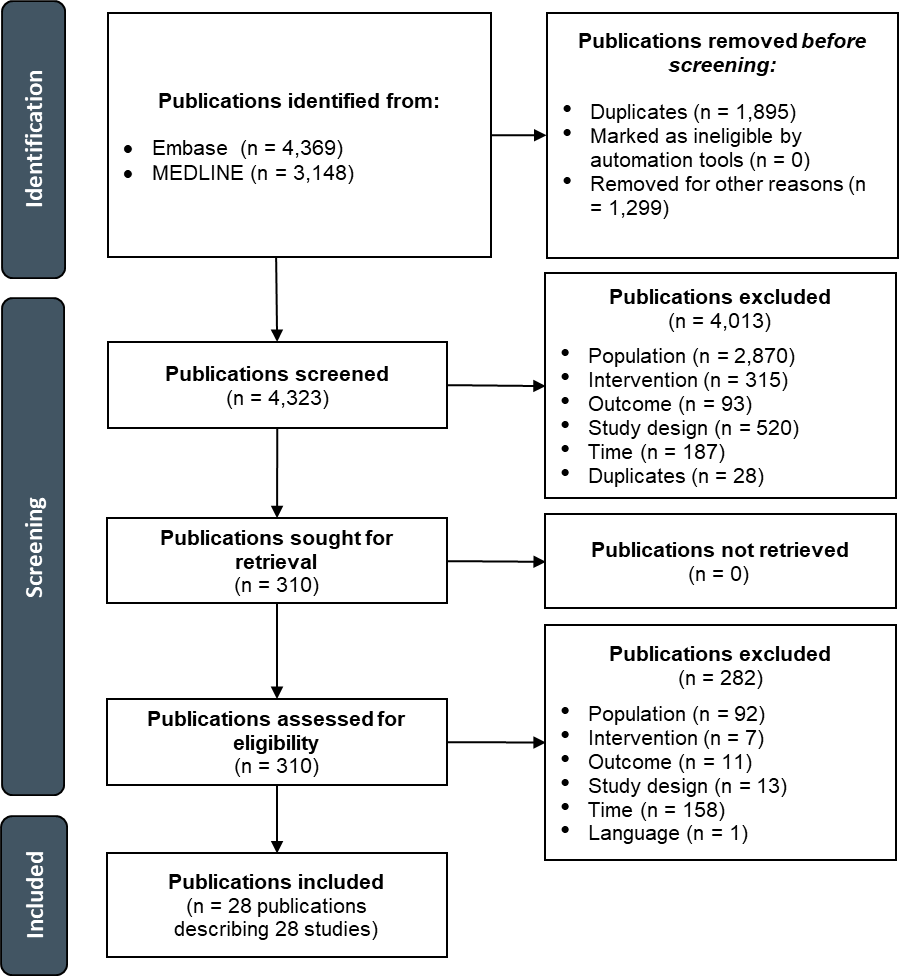


***Note:*** *“Removed for other reasons” refers to publications with irrelevant article types (e.g., conference abstracts, commentaries, review articles) according to metadata in the database export files.*

**Figure S6: PRISMA flow diagram: advanced RCC studies in the post-approval era**


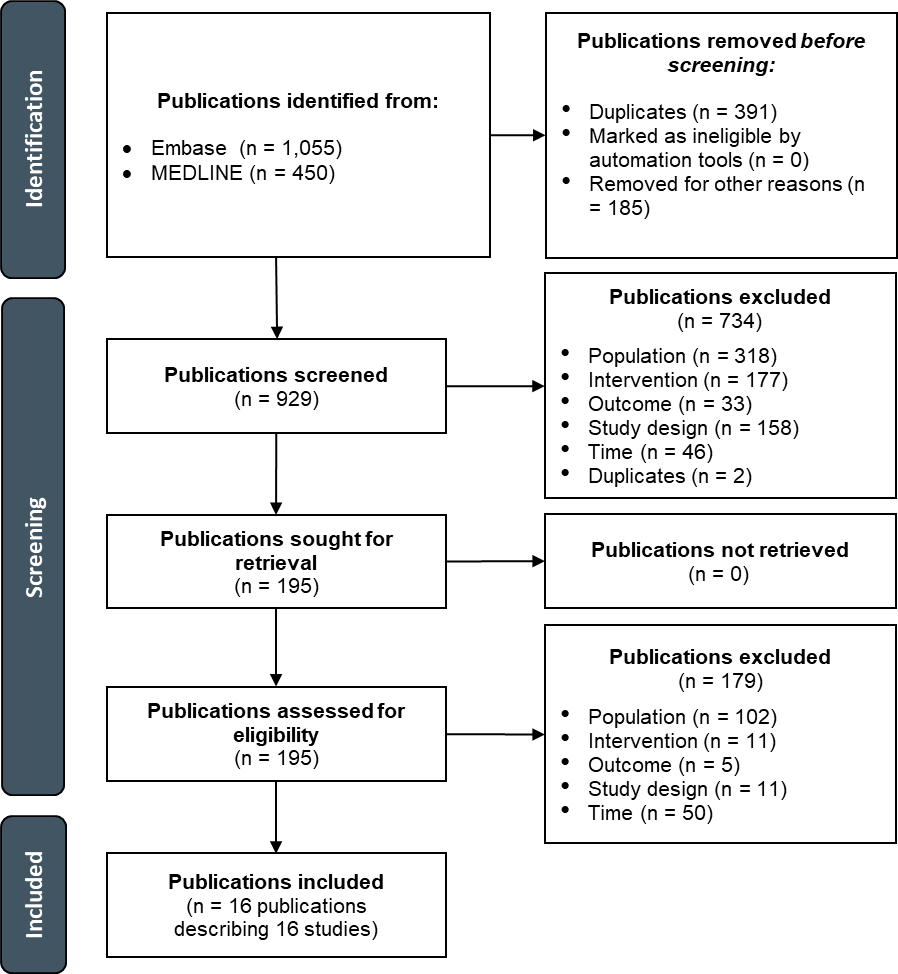


***Note:*** *“Removed for other reasons” refers to publications with irrelevant article types (e.g., conference abstracts, commentaries, review articles) according to metadata in the database export files.*

## Study Characteristics

Table S24: Study characteristics of advanced RCC studies

| **Study ID** | **Study design** | **Country/**  **region** | **Treatment (s) evaluated** | **N** |
| --- | --- | --- | --- | --- |
| **Pre-approval era** | | | | |
| Boegemann 2018 | Prospective cohort | Germany | Sunitinib | 297 |
| Buti 2017 | Retrospective cohort | Italy | Pazopanib | 37 |
| Cecere 2016 | Retrospective cohort | Italy | Pazopanib | 38 |
| Chrom 2016 | Retrospective cohort | Poland | Sunitinib, pazopanib, sorafenib | 266 |
| Coelho 2016 | Retrospective cohort | Brazil | Sunitinib | 58 |
| Din 2016 | Retrospective cohort | Egypt | Sunitinib | 49 |
| Edesa 2015 | Retrospective cohort | Egypt | Sunitinib | 44 |
| Ezz El Din 2017 | Retrospective cohort | Egypt | Sunitinib | 56 |
| Joshi 2015 | Prospective cohort | India | Sunitinib | 39 |
| Joshi 2016 | Prospective cohort | India | Sorafenib | 82 |
| Kim 2016 | Retrospective cohort | South Korea | Pazopanib | 93 |
| Kim 2018 | Retrospective cohort | South Korea | Sunitinib, pazopanib | 554 |
| Kostek 2019 | Retrospective cohort | Turkey | Sunitinib, pazopanib | 36 |
| Kucharz 2019 | Retrospective cohort | Poland | Sunitinib | 126 |
| Liu 2019 | Retrospective cohort | China | Sorafenib | 156 |
| Maraz 2018 | Retrospective cohort | Hungary | Sunitinib | 103 |
| Mir 2016 | Prospective cohort | India | Sunitinib | 50 |
| Miyake 2016 | Retrospective cohort | Japan | Sunitinib, sorafenib | 185 |
| Munarriz 2019 | Retrospective cohort | Spain | Sunitinib | 37 |
| Patel 2016 | Retrospective cohort | India | Sunitinib | 15 |
| Perez-Valderrama 2016 | Retrospective cohort | Spain | Pazopanib | 278 |
| Procopio 2019 | Prospective cohort | Global | Pazopanib | 657 |
| Poprach 2018 | Retrospective cohort | Czech Republic | Pazopanib | 426 |
| Qiu 2019 | Retrospective cohort | China | Sorafenib + interferon, interferon | 127 |
| Queiroz Muniz 2019 | Retrospective cohort | Argentina, Brazil, Chile, Colombia, and Mexico | Pazopanib | 156 |
| Sabanathan 2017 | Prospective cohort | Australia | Sunitinib | 45 |
| Saxena 2018 | Prospective cohort | India | Sorafenib | 60 |
| Yamamoto 2018 | Retrospective cohort | Japan | Sunitinib | 51 |
| **Post-approval era** | | | | |
| Harada 2023 | Retrospective cohort | Japan | Pembrolizumab + axitinib | 47 |
| Iinuma 2021 | Retrospective cohort | Japan | Nivolumab + ipilimumab | 35 |
| Iinuma 2023 | Retrospective cohort | Japan | Avelumab + axitinib, pembrolizumab + axitinib, nivolumab + cabozantinib, or pembrolizumab + lenalidomide | 51 |
| Izumi 2022 | Retrospective cohort | Japan | Nivolumab + ipilimumab | 129 |
| Jo 2022 | Retrospective cohort | Korea | Pembrolizumab + axitinib or nivolumab + ipilimumab | 61 |
| Kato 2021 | Retrospective cohort | Japan | Nivolumab + ipilimumab | 45 |
| Kato 2022 | Retrospective cohort | Japan | Nivolumab + ipilimumab | 72 |
| Kim 2023 | Prospective cohort | Korea | Pembrolizumab + axitinib, nivolumab + ipilimumab, or pembrolizumab + lenvatinib | 69 |
| Kojima 2022 | Retrospective cohort | Japan | Nivolumab + ipilimumab | 45 |
| Rebuzzi 2022 | Prospective cohort | Italy | Nivolumab + ipilimumab | 306 |
| Sang 2022 | Prospective cohort | Korea | Pembrolizumab + axitinib | 58 |
| Shah 2023 | Retrospective cohort | US | Pembrolizumab + axitinib, nivolumab + ipilimumab | 1538 |
| Tomiyama 2023 | Retrospective cohort | Japan | Nivolumab + ipilimumab | 75 |
| Ueda 2023 | Retrospective cohort | Japan | Nivolumab + ipilimumab | 46 |
| Zakharia 2022 | Retrospective cohort | US | Pembrolizumab + axitinib | 355 |
| Zarrabi 2023 | Retrospective cohort | US | Pembrolizumab + axitinib, nivolumab + ipilimumab | 1506 |

**Abbreviations:** US, United States.

## Patient Characteristics

Table S25: Patient characteristics in advanced RCC studies: age, sex, performance status, and race

| **Study ID** | **Treatment** | **Overall/**  **subgroup** | **N** | **Age, years, median (range)** | **Male, %** | **ECOG PS**  **0-1,%** | **White race, %** |
| --- | --- | --- | --- | --- | --- | --- | --- |
| **Pre-approval era** | | | | | | | |
| Boegemann 2018 | Modified sunitinib schedule | Overall | 98 | 69 (62-74)^b^ | -- | -- | -- |
|  | Standard sunitinib schedule | Overall | 199 | 65 (58-74)^b^ | -- | -- | -- |
| Buti 2017 | Pazopanib | Overall | 37 | 65 (44-80) | 70 | 84 | -- |
| Cecere 2016 | Pazopanib | Overall | 38 | 61 (--) | 57.9 | -- | -- |
| Chrom 2016 | TKI (sunitinib, pazopanib, or sorafenib) | Overall | 266 | 61 (22-85) | 67.7 | 97.7 | -- |
| Coelho 2016 | Sunitinib | Overall | 58 | 58 (18-80) | 71 | 64 | -- |
| Din 2016 | Sunitinib | Overall | 49 | 50.5 (21-71) | 61.2 | 77.5 | -- |
| Edesa 2015 | Sunitinib | Overall | 44 | 53 (18-79) | 73 | 93 | -- |
| Ezz El Din 2017 | Sunitinib (4 w on/2 w off) | Overall | 30 | 49 (25-76) | 66.7 | 83.3 | -- |
|  | Sunitinib (2 w on/1 w off) | Overall | 26 | 49.5 (21-71) | 57.5 | 61.5 | -- |
| Joshi 2015 | Sunitinib | Overall | 39 | -- (31-80) | 76.9 | -- | -- |
| Joshi 2016 | Sorafenib | Overall | 82 | 57 (21-75) | 79.5 | -- | -- |
| Kim 2016 | Pazopanib | Overall | 93 | 65 (19-84) | 77 | 88 | -- |
| Kim 2018 | Sunitinib | Overall | 293 | 59 (52-67)^b^ | 83.6 | 89.4 | -- |
|  | Pazopanib | Overall | 261 | 64 (55-72)^b^ | 72.8 | 85.1 | -- |
| Kostek 2019 | Sunitinib | Overall | 18 | 58 (48-62)^b^ | 22.2 | 94.4 | -- |
|  | Pazopanib | Overall | 18 | 61 (50-69)^b^ | 38.9 | 94.5 | -- |
| Kucharz 2019 | Sunitinib | Overall | 126 | 63.1 (10.1)^a^ | 63 | 4 | -- |
| Liu 2019 | Sorafenib | Overall | 89 | 65.35 (9.03)^a^ | 53.9 | 85.4 | -- |
| Maraz 2018 | Sunitinib | Overall | 103 | 62.27 (32-80)^c^ | 77.7 | -- | -- |
| Mir 2016 | Sunitinib | Overall | 50 | 64 (21-83) | 60 | 72 | -- |
| Miyake 2016a | Sunitinib or sorafenib | Overall | 185 | 62 (37-88) | 76.2 | -- | -- |
| Munarriz 2019 | Sunitinib | Overall | 37 | 56 (34-76) | 78.4 | -- | -- |
| Patel 2016 | Sunitinib | Overall | 15 | 51 (23-66) | 60 | 100 | -- |
| Perez-Valderrama 2016 | Pazopanib | Overall | 278 | 67 (26-92) | 68.3 | -- | -- |
| Poprach 2018 | Pazopanib | Overall | 426 | 67 (37-88) | 67.6 | 96.5 | -- |
| Procopio 2019 | Pazopanib | MSKCC intermediate risk | 363 | 67 (22-89) | 70.5 | 91.7 | 95.6 |
|  |  | IMDC intermediate risk | 343 | 67 (22-90) | 70.8 | 92.1 | 94.2 |
| Qiu 2019 | Sorafenib + interferon | Overall | 74 | -- | 55.41 | 100 | -- |
|  | Interferon | Overall | 53 | -- | 54.72 | 100 | -- |
| Queiroz Muniz 2019 | Pazopanib | Overall | 156 | 61.6 (9.1)^a^ | -- | 77.1 | -- |
| Sabanathan 2017 | Sunitinib | Overall | 45 | 62 (36-78) | 80 | 90 | -- |
| Saxena 2018 | Sorafenib | Overall | 60 | 55 (--) | 63.3 | 35 | -- |
| Yamamoto 2018 | Sunitinib | Overall | 51 | 65 (13.2)^a^ | 78.4 | -- | -- |
| **Post-approval era** | | | | | | | |
| Harada 2023 | Pembrolizumab + axitinib | Overall | 47 | 69.5 (45-82) | 80.9 | -- | -- |
| Iinuma 2021 | Nivolumab + ipilimumab | Overall | 35 | 69 (58-76)^b^ | 74.3 | 77.2 | -- |
| Iinuma 2023 | ICI + TKI | Overall | 51 | 71 (61-75)^b^ | 70.6 | 74.5 | -- |
| Izumi 2022 | Nivolumab + ipilimumab | Non-clear cell | 22 | 64 (28-87) | 77 | -- | -- |
|  |  | Clear cell | 107 | 67 (44-87) | 70 | -- | -- |
| Jo 2022 | Pembrolizumab + axitinib or nivolumab + ipilimumab | Overall | 25 | 58 (37-79) | 76 | -- | -- |
| Kato 2021 | Nivolumab + ipilimumab | Overall | 45 | 70 (50-85) | 80 | 77.8 | -- |
| Kato 2022 | Nivolumab + ipilimumab | Overall | 72 | 70 (36-86) | 84.7 | -- | -- |
| Kim 2023 | Pembrolizumab + axitinib, nivolumab + ipilimumab, or pembrolizumab + lenvatinib | Overall | 69 | 59 (19-83) | 71 | 88.4 | -- |
| Kojima 2022 | Nivolumab + ipilimumab | Overall | 45 | 70 (50-85) | 80 | 77.8 | -- |
| Rebuzzi 2022 | Nivolumab + ipilimumab | Overall | 306 | 62.2 (24-87) | 74.5 | -- | -- |
| Sang 2022 | Pembrolizumab + axitinib | Overall | 58 | 60 (39-83) | 72.4 | 87.9 | -- |
| Shah 2023 | Pembrolizumab + axitinib | Overall | 279 | 67.8 (60.6-76)^b^ | 68.8 | 50.9 | 66 |
|  | Nivolumab + ipilimumab | Overall | 641 | 65.8 (57.8-72.1)^b^ | 73.6 | 55.2 | 73.3 |
| Tomiyama 2023 | Nivolumab + ipilimumab | Overall | 75 | 71 (47-86) | 74.7 | -- | -- |
| Ueda 2023 | Nivolumab + ipilimumab | Overall | 46 | 66.5 (42-80) | 84.8 | 84.8 | -- |
| Zakharia 2022 | Pembrolizumab + axitinib | Overall | 355 | 68 (60-75)^b^ | 69.58 | 68.17 | 67.89 |
| Zarrabi 2023 | Pembrolizumab + axitinib | Overall | 547 | 67 (21-85) | 70.2 | -- | 66 |
|  | Nivolumab + ipilimumab | Overall | 959 | 65 (29-84) | 75 | -- | 63.6 |

**Notes: a)** Mean (standard deviation). **b)** Median (interquartile range). **c)** Mean (range). **Abbreviations:** ECOG, Eastern Cooperative Oncology Group; ICI, immune checkpoint inhibitor; IMDC, International mRCC Database Consortium; MSKCC, Memorial Sloan Kettering Cancer Center; TKI, tyrosine kinase inhibitor; w, week.

Table S26: Patient characteristics in advanced RCC studies: IMDC/MSKCC risk classification

| **Study ID** | **Treatment** | **Overall/**  **subgroup** | **N** | **IMDC/MSCKK risk classification** | | | | |
| --- | --- | --- | --- | --- | --- | --- | --- | --- |
|  |  |  |  | **High, %** | **Intermediate/high, %** | **Intermediate, %** | **Intermediate/low, %** | **Low, %** |
| **Pre-approval era** | | | | | | | | |
| Boegemann 2018 | Modified sunitinib schedule | Overall | 98 | 44.9 | -- | 51.4 | -- | 3.7 |
|  | Standard sunitinib schedule | Overall | 199 | 18.9 | -- | 73.6 | -- | 7.5 |
| Buti 2017 | Pazopanib | Overall | 37 | 22 | -- | 49 | -- | 24 |
| Cecere 2016 | Pazopanib | Overall | 38 | 12.5 | -- | 63.1 | -- | 13.1 |
| Chrom 2016 | TKI (sunitinib, pazopanib, or sorafenib) | Overall | 266 | 22.4 | -- | 59.9 | -- | 17.7 |
| Coelho 2016 | Sunitinib | Overall | 58 | 28 | -- | 38 | -- | 33 |
| Din 2016 | Sunitinib | Overall | 49 | 14.3 | -- | 57.1 | -- | 28.6 |
| Edesa 2015 | Sunitinib | Overall | 44 | 9 | -- | 66 | -- | 25 |
| Ezz El Din 2017 | Sunitinib (4 w on/2 w off) | Overall | 30 | 10 | -- | 63.3 | -- | 26.7 |
|  | Sunitinib (2 w on/1 w off) | Overall | 26 | 15.4 | -- | 53.8 | -- | 30.8 |
| Joshi 2015 | Sunitinib | Overall | 39 | -- | -- | -- | -- | -- |
| Joshi 2016 | Sorafenib | Overall | 82 | 34.1 | -- | 34.1 | -- | 23.2 |
| Kim 2016 | Pazopanib | Overall | 93 | 17 | -- | 40 | -- | 43 |
| Kim 2018 | Sunitinib | Overall | 293 | 16.9 | -- | 61.3 | -- | 20.7 |
|  | Pazopanib | Overall | 261 | 15.7 | -- | 63.1 | -- | 20.5 |
| Kostek 2019 | Sunitinib | Overall | 18 | 11.1 | -- | 83.3 | -- | 5.6 |
|  | Pazopanib | Overall | 18 | 22.2 | -- | 72.2 | -- | 5.6 |
| Kucharz 2019 | Sunitinib | Overall | 126 | 3 | -- | 29 | -- | 65 |
| Liu 2019 | Sorafenib | Overall | 89 | -- | -- | -- | -- | -- |
| Maraz 2018 | Sunitinib | Overall | 103 | -- | -- | -- | -- | -- |
| Mir 2016 | Sunitinib | Overall | 50 | 56 | -- | 36 | -- | 8 |
| Miyake 2016a | Sunitinib or sorafenib | Overall | 185 | 18.4 | -- | 57.8 | -- | 23.8 |
| Munarriz 2019 | Sunitinib | Overall | 37 | -- | -- | -- | -- | -- |
| Patel 2016 | Sunitinib | Overall | 15 | -- | 93 | -- | -- | 7 |
| Perez-Valderrama 2016 | Pazopanib | Overall | 278 | 23.4 | -- | 57.2 | -- | 19.4 |
| Poprach 2018 | Pazopanib | Overall | 426 | 4.9 | -- | 31.5 | -- | 15 |
| Procopio 2019 | Pazopanib | MSKCC intermediate risk | 363 | 0 | -- | 100 | -- | 0 |
|  |  | IMDC intermediate risk | 343 | 0 | -- | 100 | -- | 0 |
| Qiu 2019 | Sorafenib + interferon | Overall | 74 | -- | -- | -- | -- | -- |
|  | Interferon | Overall | 53 | -- | -- | -- | -- | -- |
| Queiroz Muniz 2019 | Pazopanib | Overall | 156 | 18.9 | -- | 61.4 | -- | 19.7 |
| Sabanathan 2017 | Sunitinib | Overall | 45 | 15 | -- | 47 | -- | 38 |
| Saxena 2018 | Sorafenib | Overall | 60 | 40 | -- | 48.33 | -- | 11.67 |
| Yamamoto 2018 | Sunitinib | Overall | 51 | 23.5 | -- | 70.6 | -- | 5.9 |
| **Post-approval era** | | | | | | | | |
| Harada 2023 | Pembrolizumab + axitinib | Overall | 47 | 34 | -- | 51 | -- | 14.9 |
| Iinuma 2021 | Nivolumab + ipilimumab | Overall | 35 | 34.3 | -- | 65.7 | -- | -- |
| Iinuma 2023 | ICI + TKI | Overall | 51 | 21.6 | -- | 54.9 | -- | 23.5 |
| Izumi 2022 | Nivolumab + ipilimumab | Non-clear cell | 22 | 41 | -- | 59 | -- | -- |
|  | Nivolumab + ipilimumab | Clear cell | 107 | 39 | -- | 61 | -- | -- |
| Jo 2022 | Pembrolizumab + axitinib or nivolumab + ipilimumab | Overall | 25 | 100 | -- | 0 | -- | 0 |
| Kato 2021 | Nivolumab + ipilimumab | Overall | 45 | 51.1 | -- | 48.9 | -- | -- |
| Kato 2022 | Nivolumab + ipilimumab | Overall | 72 | 30.6 | -- | 69.4 | -- | -- |
| Kim 2023 | Pembrolizumab + axitinib, nivolumab + ipilimumab, or pembrolizumab + lenvatinib | Overall | 69 | 29 | -- | 49.3 | -- | 21.7 |
| Kojima 2022 | Nivolumab + ipilimumab | Overall | 45 | 50 | -- | 50 | -- | -- |
| Rebuzzi 2022 | Nivolumab + ipilimumab | Overall | 306 | 32.7 | -- | 67.3 | -- | -- |
| Sang 2022 | Pembrolizumab + axitinib | Overall | 58 | 31 | -- | 50 | -- | 37.9 |
| Shah 2023 | Pembrolizumab + Axitinib | Overall | 279 | -- | 85.7 | -- | 11.8 | -- |
|  | Nivolumab + ipilimumab | Overall | 641 | -- | 93.6 | -- | 5.3 | -- |
| Tomiyama 2023 | Nivolumab + ipilimumab | Overall | 75 | 42.7 | -- | 57.3 | -- | -- |
| Ueda 2023 | Nivolumab + ipilimumab | Overall | 46 | 50 | -- | 50 | -- | -- |
| Zakharia 2022 | Pembrolizumab + axitinib | Overall | 355 | 21.41 | -- | 35.49 | -- | 7.61 |
| Zarrabi 2023 | Pembrolizumab + axitinib | Overall | 547 | -- | -- | -- | -- | 8.6 |
|  | Nivolumab + ipilimumab | Overall | 959 | -- | -- | -- | -- | 5.1 |

**Abbreviations:** ICI, immune checkpoint inhibitor; IMDC, International mRCC Database Consortium; MSKCC, Memorial Sloan Kettering Cancer Center; TKI, tyrosine kinase inhibitor; w, week.

## Outcomes

Table S27: Summary of mOS in advanced RCC studies

|  | **Pre-approval era** | | | **Post-approval era** | | |
| --- | --- | --- | --- | --- | --- | --- |
|  | **No. treatment groups** | **mOS in months, range** | **Median follow up in months, range** | **No. treatment groups** | **mOS in months, range** | **Median follow up in months, range** |
| **IMDC/MSKCC risk classification** | | | | | | |
| High^a^ | 7 | 2-10.3 | 11.2-46.1 | 4 | 7.8-24.3 | 8.96-24 |
| Intermediate/high^a^ | 3 | 9.7-35.6 | Not reported | 10 | 20.8-NR | 7.2-20 |
| Intermediate^a^ | 9 | 12-35.9 | 11.2-46.1 | 3 | NR | 8.96-24 |
| Intermediate/low^a^ | 1 | 26.5 | 21 | 0 | -- | -- |
| Low^a^ | 6 | 19.6-NR | 11.2-46.1 | 2 | NR | 20-20 |
| Mixed^b^ | 15 | 13.2-40.2 | 12-30 | 3 | 21.9-NR | 7-12.1 |
| Unspecified^c^ | 8 | 12.5-52.97 | 24.37-28.8 | 0 | -- | -- |

***Notes:*** *a) Treatment groups including ≥80% of patients with the given risk classification. b) Treatment groups including <80% of patients with any given risk classification. c) Treatment groups in studies in which risk classification was neither an eligibility criterion nor reported as a patient characteristic.* ***Abbreviations:*** *IMDC, International mRCC Database Consortium; mOS, median overall survival; MSKCC, Memorial Sloan Kettering Cancer Center; no., number; NR, not reached.*

Table S28: Overall survival by treatment group in advanced RCC studies

| **Study ID** | **Treatment** | **Overall/**  **subgroup** | **Follow-up duration, months (range)** | **Median OS, months (95% CI)** | **Landmark OS** | | | | | |  |
| --- | --- | --- | --- | --- | --- | --- | --- | --- | --- | --- | --- |
|  |  |  |  |  | **3-mo** | **6-mo** | **9-mo** | **12-mo** | **18-mo** | **2-y** | **3-y** |
| **Pre-approval era** | | | | | | | | | | | |
| Boegemann 2018 | Modified sunitinib schedule | Overall | -- | 38.1 (28.9-50.5) | -- | -- | -- | -- | -- | -- | -- |
|  | Standard sunitinib schedule | Overall | -- | 13.7 (10.1-20.2) | -- | -- | -- | -- | -- | -- | -- |
|  | Modified sunitinib schedule | Intermediate/poor risk (IMDC) | -- | 35.6 (19.6-NR) | -- | -- | -- | -- | -- | -- | -- |
|  | Standard sunitinib schedule | Intermediate/poor risk (IMDC) | -- | 9.7 (8.5-15.9) | -- | -- | -- | -- | -- | -- | -- |
| Buti 2017 | Pazopanib | Overall | 24.4 (16.6-31.1)^a^ | 17.3 (11.5-23) | -- | -- | -- | 64 | -- | 35 | -- |
| Cecere 2016 | Pazopanib | Overall | -- | 26.2 (12.6-39.9) | -- | -- | -- | -- | -- | -- | -- |
| Chrom 2016 | TKI (sunitinib, pazopanib, or sorafenib) | Overall | 46.1 (41.2-51)^a^ | 24.8 (20.2-29.4) | -- | -- | -- | -- | -- | -- | -- |
|  | Sunitinib | Overall |  | 24.8 (--) | -- | -- | -- | -- | -- | -- | -- |
|  | Pazopanib | Overall |  | 17.0 (--) | -- | -- | -- | -- | -- | -- | -- |
|  | Sorafenib | Overall |  | 38.7 (--) | -- | -- | -- | -- | -- | -- | -- |
|  | TKI (sunitinib, pazopanib, or sorafenib) | Low risk (IMDC) |  | NR | -- | -- | -- | -- | -- | -- | -- |
|  |  | Intermediate risk (IMDC) |  | 35.9 (27.9-43.0) | -- | -- | -- | -- | -- | -- | -- |
|  |  | High risk (IMDC) |  | 8.8 (5.6-12) | -- | -- | -- | -- | -- | -- | -- |
| Coelho 2016 | Sunitinib | Overall | -- | 14.1 (--) | -- | -- | -- | -- | -- | -- | -- |
| Din 2016 | Sunitinib | Overall | 16 (4-34) | 15 (--) | -- | -- | -- | -- | -- | -- | -- |
| Edesa 2015 | Sunitinib | Overall | 19 (4.5-54.5) | 23 (15.2-30.9) | -- | -- | -- | -- | -- | -- | -- |
| Ezz El Din 2017 | Sunitinib | Overall | 30 (29.5-30) | 24 (2-42)^c^ | -- | -- | -- | -- | -- | -- | -- |
|  | Sunitinib | Overall |  | 23 (3-43)^c^ | -- | -- | -- | -- | -- | -- | -- |
| Joshi 2015 | Sunitinib | Overall | -- | 28.5 (9.253-47.7) | -- | -- | -- | -- | -- | -- | -- |
| Joshi 2016 | Sorafenib | Overall | 11.2 (1-55) | 12.18 (9.61-14.76)^c^ | -- | -- | -- | -- | -- | -- | -- |
|  |  | Low risk (IMDC) |  | 19.6 (8.5-30.7 ^c^ | -- | -- | -- | -- | -- | -- | -- |
|  |  | Intermediate risk (IMDC) |  | 16.1 (7.6-24.7)^c^ | -- | -- | -- | -- | -- | -- | -- |
|  |  | High risk (IMDC) |  | 10.3 (9.5-11.1)^c^ | -- | -- | -- | -- | -- | -- | -- |
| Kim 2016 | Pazopanib | Overall | 21 (--) | 21.9 (12.9-30.9) | -- | -- | -- | -- | -- | -- | -- |
|  |  | High risk (MSKCC) |  | 7.2 (1.5-12.9) | -- | -- | -- | -- | -- | -- | -- |
|  |  | Intermediate/low risk (MSKCC) |  | 26.5 (18.9-34.1) | -- | -- | -- | -- | -- | -- | -- |
| Kim 2018 | Sunitinib | Overall | 16.4 (14.7-17.8)^a^ | 36.5 (29-47.9) | -- | -- | -- | -- | -- | -- | -- |
|  | Pazopanib | Overall |  | 40.2 (31.1-51.2) | -- | -- | -- | -- | -- | -- | -- |
| Kostek 2019 | Sunitinib | Overall | -- | 28.6 (24.3-32.9) | -- | -- | -- | -- | -- | -- | -- |
|  | Pazopanib | Overall | -- | 25.5 (18.9-52.7) | -- | -- | -- | -- | -- | -- | -- |
| Kucharz 2019 | Sunitinib | Overall | 23 (3-70) | 31 (17-NR)^d^ | -- | -- | -- | -- | -- | -- | -- |
| Liu 2019 | Sorafenib | Overall | 28.8 (7-67) | 29 (--) | -- | -- | -- | -- | -- | -- | -- |
| Maraz 2018 | Sunitinib | Overall | 24.37 (1.33-93.83) | 25.36 (20.23-30.5) | -- | -- | -- | -- | -- | -- | -- |
| Mir 2016 | Sunitinib | Overall | 12 (1-48) | 13.2 (10.1-16.5) | -- | -- | -- | -- | -- | -- | -- |
| Miyake 2016a | Sunitinib or sorafenib | Overall | -- | 33.6 (--) | -- | -- | -- | -- | -- | -- | -- |
| Munarriz 2019 | Sunitinib | Overall | -- | 52.97 (38.07-67.86) | -- | -- | -- | -- | -- | -- | -- |
| Patel 2016 | Sunitinib | Overall | -- | 12 (3-24)^c^ | -- | -- | -- | -- | -- | -- | -- |
| Perez-Valderrama 2016 | Pazopanib | Overall | 23 (--) | 22.2 (16-29) | -- | -- | -- | -- | -- | 48.1 | -- |
|  |  | Low risk (IMDC) |  | NR (--) | -- | -- | -- | -- | -- | 81.6 | -- |
|  |  | Intermediate risk (IMDC) |  | 21.6 (--) | -- | -- | -- | -- | -- | 48.7 | -- |
|  |  | High risk (IMDC) |  | 7.1 (--) | -- | -- | -- | -- | -- | 18.4 | -- |
| Procopio 2019 | Pazopanib | Intermediate risk: 1 risk factor (IMDC) | 30 (--) | 33.9 (33.9-NR) | -- | -- | -- | -- | -- | -- | -- |
|  |  | Intermediate risk: 2 risk factors (IMDC) |  | 19.4 (14.3-NR) | -- | -- | -- | -- | -- | -- | -- |
|  |  | Intermediate risk: 1 risk factor (MSKCC |  | NR (NR-NR) | -- | -- | -- | -- | -- | -- | -- |
|  |  | Intermediate risk: 2 risk factors (MSKCC) |  | 15.2 (12.3-26.5) | -- | -- | -- | -- | -- | -- | -- |
| Poprach 2018 | Pazopanib | Overall | -- | 33.2 (29.9-36.4) | -- | 92.5 | -- | 83.3 | -- | 63 | -- |
|  |  | Low risk (IMDC) | -- | NR | -- | -- | -- | -- | -- | -- | -- |
|  |  | Intermediate risk (IMDC) | -- | 31.4 (23.4-39.3) | -- | -- | -- | -- | -- | -- | -- |
|  |  | High risk (IMDC) | -- | 9.2 (3.2-15.1) | -- | -- | -- | -- | -- | -- | -- |
|  |  | Low risk (MSKCC) | -- | NR | -- | -- | -- | -- | -- | -- | -- |
|  |  | Intermediate risk (MSKCC) | -- | 28.5 (24-32.9) | -- | -- | -- | -- | -- | -- | -- |
|  |  | High risk (MSKCC) | -- | 15.1 (5-25.3) | -- | -- | -- | -- | -- | -- | -- |
| Qiu 2019 | Sorafenib + interferon | Overall | -- | 15.3 (9-60)^c^ | -- | -- | -- | 97.84 | -- | -- | 68.92 |
|  | Interferon | Overall | -- | 12.5 (8-60)^c^ | -- | -- | -- | 73.56 | -- | -- | 47.17 |
| Queiroz Muniz 2019 | Pazopanib | Overall | -- | 16.9 (--) | -- | -- | -- | -- | -- | -- | -- |
|  |  | Intermediate risk (MSKCC) | -- | 15.6 (--) | -- | -- | -- | -- | -- | -- | -- |
|  |  | Low risk (MSKCC) | -- | NR (--) | -- | -- | -- | -- | -- | -- | -- |
|  |  | High risk (MSKCC) | -- | 8.9 (--) | -- | -- | -- | -- | -- | -- | -- |
| Sabanathan 2017 | Sunitinib | Overall | 30 (--)^b^ | 32 (--) | -- | -- | -- | -- | -- | -- | -- |
| Saxena 2018 | Sorafenib | Overall | -- | 8 (0.5-42)^c^ | -- | -- | -- | 43.33 | -- | -- | -- |
|  |  | Low risk (IMDC) | -- | 36 (--) | -- | -- | -- | -- | -- | -- | -- |
|  |  | Intermediate risk (IMDC) | -- | 12 (--) | -- | -- | -- | -- | -- | -- | -- |
|  |  | High risk (IMDC) | -- | 2 (--) | -- | -- | -- | -- | -- | -- | -- |
|  |  | Low risk (MSKCC) | -- | 36 (--) | -- | -- | -- | -- | -- | -- | -- |
|  |  | Intermediate risk (MSKCC) | -- | 12 (--) | -- | -- | -- | -- | -- | -- | -- |
|  |  | High risk (MSKCC) | -- | 2 (--) | -- | -- | -- | -- | -- | -- | -- |
| Yamamoto 2018 | Sunitinib | Overall | -- | 38 (15.4-NR) | -- | -- | -- | -- | -- | -- | -- |
| **Post-approval era** | | | | | | | | | | | |
| Harada 2023 | Pembrolizumab + axitinib | Overall | 14 (--) | NR (--) | -- | -- | -- | -- | -- | -- | -- |
| Iinuma 2021 | Nivolumab + ipilimumab | Overall | 12 (4.5-16)^d^ | NR (--) | -- | 100 | -- | 95.8 | 87.1 | -- | -- |
| Iinuma 2023 | ICI + TKI | Overall | 7 (4-13)^d^ | NR (--) | -- | 93.1 | -- | 82.5 | 68.8 | -- | -- |
| Izumi 2022 | Nivolumab + ipilimumab | Non-clear cell | 11.1 (2.2-31.7) | 20.8 (5-NR) | -- | -- | -- | -- | -- | 34 | -- |
|  |  | Clear cell | 12.4 (0.1-36.3) | NR (NR-NR) | -- | -- | -- | -- | -- | 62 | -- |
| Jo 2022 | Pembrolizumab + axitinib or nivolumab + ipilimumab | Overall | 23 (22.1-24.4)^a^ | 24.3 (--) | -- | -- | -- | -- | -- | -- | -- |
| Kato 2021 | Nivolumab + ipilimumab | Overall | 13.8 (0.3-16.6) | NR (NR-NR) | -- | -- | -- | 81.4 | -- | -- | -- |
| Kato 2022 | Nivolumab + ipilimumab | Overall | 16.1 (1.4-37.8) | NR (--) | -- | -- | -- | -- | -- | 67.4 | -- |
| Kim 2023 | Pembrolizumab + axitinib, nivolumab + ipilimumab, or pembrolizumab + lenvatinib | Overall | 12.1 (--) | -- | -- | -- | -- | 77.9 | -- | -- | -- |
| Kojima 2022 | Nivolumab + ipilimumab | Overall | 24 (0.3-28.3) | NR (NR-NR) | -- | -- | -- | -- | -- | 59.1 | -- |
|  |  | Intermediate risk (IMDC) |  | NR (NR-NR) | -- | -- | -- | -- | -- | 75.4 | -- |
|  |  | High risk (IMDC) |  | 17.7 (9.8-NR) | -- | -- | -- | -- | -- | 43.1 | -- |
| Rebuzzi 2022 | Nivolumab + ipilimumab | Overall | 12.2 (4.7-17.3)^d^ | NR (--) | -- | -- | -- | 66.8 | -- | -- | -- |
|  |  | Intermediate risk (IMDC) |  | NR (--) | -- | -- | -- | 77.3 | -- | -- | -- |
|  |  | High risk (IMDC) |  | 7.8 (--) | -- | -- | -- | 41.8 | -- | -- | -- |
| Sang 2022 | Pembrolizumab + axitinib | Overall | 12.1 (8-17.4)^d^ | 21.9 (18.5-25.3) | -- | -- | -- | -- | -- | -- | -- |
| Shah 2023 | Pembrolizumab + axitinib | Overall | 7.2 (4.2-11.8)^d^ | NR (--) | -- | -- | -- | 76 | -- | -- | -- |
| Shah 2023 | Nivolumab + ipilimumab | Overall | 8.5 (3.5-15.7)^d^ | 27.6 (21.7-30.9) | -- | -- | -- | 73 | -- | -- | -- |
| Tomiyama 2023 | Nivolumab + ipilimumab | Overall | 8.96 (0.75-29.33) | 29.3 (--) | -- | -- | -- | -- | -- | -- | -- |
|  |  | Intermediate risk (IMDC) |  | NR (--) | -- | -- | -- | -- | -- | -- | -- |
|  |  | High risk (IMDC) |  | 18.3 (--) | -- | -- | -- | -- | -- | -- | -- |
| Ueda 2023 | Nivolumab + ipilimumab | Overall | -- | 26.9 (22.9-NR) | -- | -- | -- | -- | -- | -- | -- |
| Zakharia 2022 | Pembrolizumab + axitinib | Overall | 9.67 (4.37-14.83)^d^ | NR (17.63-NR) | 90.75 | 85.7 | 78.89 | 73.54 | -- | -- | -- |
| Zarrabi 2023 | Pembrolizumab + axitinib | Overall | 20 (0.2-47.6) | 28.9 (23.5-NR) | -- | -- | -- | -- | -- | -- | -- |
|  |  | Low risk (IMDC) |  | NR (NR-NR) | -- | -- | -- | -- | -- | -- | -- |
|  |  | Intermediate/poor risk (IMDC) |  | 23.3 (18.4-30.1) | -- | -- | -- | -- | -- | -- | -- |
|  | Nivolumab + ipilimumab | Overall |  | 24.3 (22-28.5) | -- | -- | -- | -- | -- | -- | -- |
|  |  | Low risk (IMDC) |  | NR (37.7-NR) | -- | -- | -- | -- | -- | -- | -- |
|  |  | Intermediate/poor risk (IMDC) |  | 23.3 (20.4-26) | -- | -- | -- | -- | -- | -- | -- |

**Notes: a)** Median (95% confidence interval). **b)** Reported in weeks. **c)** Median (range). **d)** Median (interquartile range). **Abbreviations:** CI, confidence interval; IMDC, International mRCC Database Consortium; mo, month; MSKCC, Memorial Sloan Kettering Cancer Center; NR, not reached; OS, overall survival; y, year.

## Quality Assessment

Table S29: Quality assessment of advanced RCC studies

| **Study ID** | **Selection** | | | | **Comparability** | **Outcome** | | | **Total score** |
| --- | --- | --- | --- | --- | --- | --- | --- | --- | --- |
|  | **1** | **2** | **3** | **4** | **1** | **1** | **2** | **3** |  |
| **Pre-approval era** | | | | | | | | | |
| Boegemann 2018 | 1 | NA | 1 | 1 | NA | 1 | 1 | 1 | 6 |
| Buti 2017 | 1 | NA | 1 | 1 | NA | 1 | 1 | 1 | 6 |
| Cecere 2016 | 0 | NA | 1 | 1 | NA | 1 | 1 | 1 | 5 |
| Chrom 2016 | 0 | NA | 1 | 1 | NA | 1 | 1 | 1 | 5 |
| Coelho 2016 | 0 | NA | 1 | 1 | NA | 1 | 1 | 1 | 5 |
| Din 2016 | 0 | NA | 1 | 1 | NA | 1 | 1 | 1 | 5 |
| Edesa 2015 | 0 | NA | 1 | 1 | NA | 1 | 1 | 0 | 4 |
| Ezz 2017 | 0 | NA | 1 | 1 | NA | 1 | 1 | 1 | 5 |
| Joshi 2015 | 0 | NA | 1 | 0 | NA | 1 | 1 | 1 | 4 |
| Joshi 2016 | 0 | NA | 1 | 0 | NA | 1 | 1 | 1 | 4 |
| Kim 2016 | 1 | NA | 1 | 1 | NA | 1 | 1 | 1 | 6 |
| Kim 2018 | 1 | NA | 1 | 1 | NA | 1 | 1 | 1 | 6 |
| Kostek 2019 | 0 | NA | 1 | 1 | NA | 1 | 1 | 1 | 5 |
| Kucharz 2019 | 0 | NA | 1 | 1 | NA | 1 | 1 | 1 | 5 |
| Liu 2019 | 0 | NA | 1 | 0 | NA | 1 | 1 | 1 | 4 |
| Maraz 2019 | 1 | NA | 1 | 1 | NA | 1 | 1 | 1 | 6 |
| Mir 2016 | 0 | NA | 1 | 0 | NA | 1 | 1 | 1 | 4 |
| Miyake 2017 | 0 | NA | 1 | 1 | NA | 1 | 1 | 1 | 5 |
| Munarriz 2019 | 1 | NA | 1 | 1 | NA | 1 | 1 | 1 | 6 |
| Patel 2016 | 0 | NA | 1 | 1 | NA | 1 | 1 | 1 | 5 |
| Perez Martinez 2016 | 1 | NA | 1 | 1 | NA | 1 | 1 | 1 | 6 |
| Procopio 2019 | 1 | NA | 0 | 0 | NA | 0 | 1 | 1 | 3 |
| Poprach 2018 | 1 | NA | 1 | 1 | NA | 1 | 1 | 1 | 6 |
| Qiu 2019 | 0 | NA | 1 | 1 | NA | 1 | 1 | 1 | 5 |
| Queiroz Muniz 2019 | 1 | NA | 1 | 1 | NA | 1 | 1 | 1 | 6 |
| Sabanathan 2017 | 0 | NA | 1 | 0 | NA | 1 | 1 | 1 | 4 |
| Saxena 2018 | 0 | NA | 1 | 0 | NA | 1 | 1 | 0 | 3 |
| Yamamoto 2018 | 0 | NA | 1 | 1 | NA | 1 | 1 | 1 | 5 |
| **Post-approval era** | | | | | | | | | |
| Harada 2023 | 0 | NA | 1 | 1 | NA | 1 | 1 | 1 | 5 |
| Iinuma 2021 | 1 | NA | 1 | 1 | NA | 1 | 1 | 1 | 6 |
| Iinuma 2023 | 1 | NA | 1 | 1 | NA | 1 | 1 | 1 | 6 |
| Izumi 2022 | 1 | NA | 1 | 1 | NA | 1 | 1 | 1 | 6 |
| Jo 2022 | 0 | NA | 1 | 1 | NA | 1 | 1 | 1 | 5 |
| Kato 2021 | 1 | NA | 1 | 1 | NA | 1 | 1 | 1 | 6 |
| Kato 2022 | 1 | NA | 1 | 1 | NA | 1 | 0 | 1 | 5 |
| Kim 2023 | 0 | NA | 1 | 0 | NA | 1 | 1 | 1 | 4 |
| Kojima 2022 | 1 | NA | 1 | 1 | NA | 1 | 0 | 1 | 5 |
| Meet-URO 15 | 1 | NA | 1 | 1 | NA | 1 | 1 | 1 | 6 |
| Sang 2022 | 0 | NA | 0 | 0 | NA | 0 | 1 | 1 | 2 |
| Shah 2023 | 1 | NA | 1 | 1 | NA | 1 | 1 | 1 | 6 |
| Tomiyama 2023 | 1 | NA | 1 | 1 | NA | 1 | 1 | 1 | 6 |
| Ueda 2023 | 0 | NA | 1 | 1 | NA | 1 | 0 | 1 | 4 |
| Zakharia 2022 | 1 | NA | 1 | 1 | NA | 1 | 0 | 0 | 4 |
| Zarrabi 2023 | 1 | NA | 1 | 1 | NA | 1 | 1 | 1 | 6 |

***Abbreviations:*** *NA, not applicable.*
